# Repurposing of a library for high-content screening of inhibitors against *Echinococcus granulosus*

**DOI:** 10.1186/s13071-024-06456-6

**Published:** 2024-09-03

**Authors:** Weinan Zheng, Gaofei Lv, Jun Li, Yao Zhang, Wenjing Qi, Mingzhi Yan, Jinzhi Wu, Chikin Chan, Xiaoben Pan, Wenbao Zhang

**Affiliations:** 1 Department of Disease Biology, Global Health Drug Discovery Institute, Beijing, 100000 China; 2 State Key Laboratory Incubation Base of Xinjiang Major Diseases Research, Clinical Medical Research Institute, First Affiliated Hospital of Xinjiang Medical University, Urumqi, 830054 China

**Keywords:** Cystic echinococcosis, *Echinococcus granulosus*, Protoscolex, Cyst, Omaveloxolone

## Abstract

**Background:**

Cystic echinococcosis (CE) is a zoonotic disease caused by the larval stage of the dog tapeworm *Echinococcus granulosus *sensu lato (*E. granulosus*), with a worldwide distribution. The current treatment strategy for CE is insufficient. Limited drug screening models severely hamper the discovery of effective anti-echinococcosis drugs.

**Methods:**

In the present study, using high-content screening technology, we developed a novel high-throughput screening (HTS) assay by counting the ratio of propidium iodide-stained dead protoscoleces (PSCs) to the total number of PSCs. In vitro and ex vivo cyst viability assays were utilized to determine the effect of drugs on cyst viability.

**Results:**

Using the newly established HTS assay, we screened approximately 12,000 clinical-stage or The Food and Drug Administration (FDA)-approved small molecules from the Repurposing, Focused Rescue, and Accelerated Medchem (ReFRAME) library, as well as the LOPAC1280 and SelleckChem libraries, as a strategic approach to facilitate the drug discovery process. Initial screening yielded 173 compounds with anti-echinococcal properties, 52 of which demonstrated dose–response efficacy against *E. granulosus* PSCs in vitro. Notably, two agents, omaveloxolone and niclosamide, showed complete inhibition upon further validation in cyst and microcyst viability assays in vitro after incubation for 3 days, and in an ex vivo cyst viability assay using cysts isolated from the livers of mice infected with *E. granulosus*, as determined by morphological assessment.

**Conclusions:**

Through the development of a novel HTS assay and by repurposing libraries, we identified omaveloxolone and niclosamide as potent inhibitors against *E. granulosus*. These compounds show promise as potential anti-echinococcal drugs, and our strategic approach has the potential to promote drug discovery for parasitic infections.

**Graphical Abstract:**

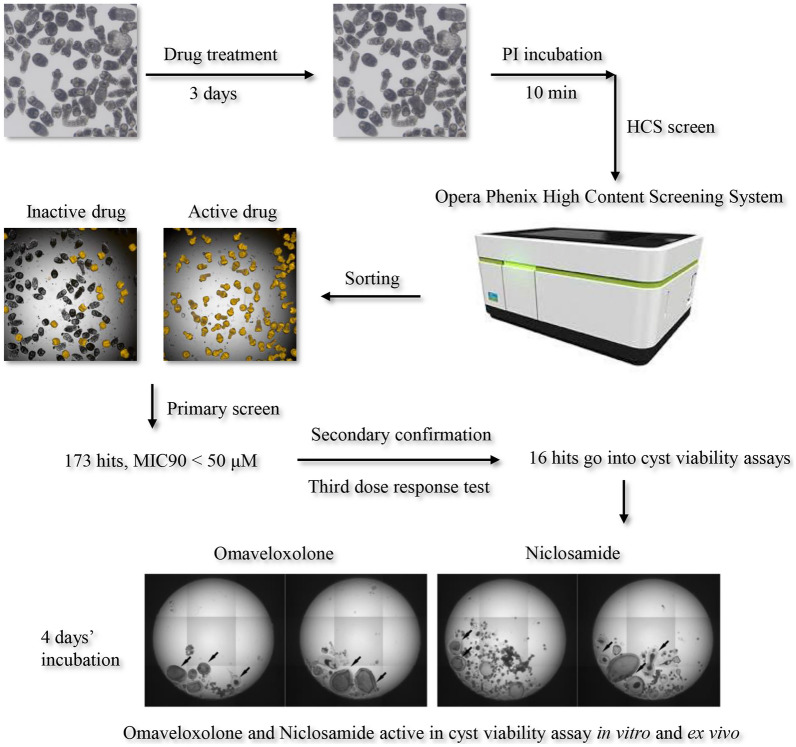

**Supplementary Information:**

The online version contains supplementary material available at 10.1186/s13071-024-06456-6.

## Background

Cystic echinococcosis (CE) is a zoonosis that is caused by infection with the larval stage of the tapeworm *Echinococcus granulosus *sensu lato (*s.l.*). The disease is found worldwide and predominantly affects regions such as the Mediterranean, Central Asia, North and East Africa, and South America [1]. Parasites are acquired via oral uptake of eggs containing infectious oncospheres [2]. Subsequently, these oncospheres invade the liver and other organs, where they establish the disease-causing stage, the *Echinococcus* metacestode. Natural intermediate hosts of *E. multilocularis* are rodents and other small mammals, whereas *E. granulosus s.l.* naturally infects a variety of larger mammals, such as sheep and cattle, depending on the parasite genotype. Inside metacestodes, protoscoleces (PSCs) develop that will differentiate into adult worms when ingested by definitive hosts such as foxes or dogs [2]. *E. granulosus s.l.* can also infect aberrant hosts that usually do not further transmit the parasite, but still develop CE. *E. granulosus s.l.* thereby imposes a considerable burden on human and veterinary health and is responsible for high economic losses, in particular in the case of CE [3].

Humans, as accidental intermediate hosts of *E. granulosus*, can be infected by ingesting the eggs of the tapeworm in the feces of definitive hosts. These eggs develop into oncospheres, which then develop into larval cysts within the internal organs of the intermediate hosts. In humans, unilocular hydatid cysts are found primarily in the liver (70%) and lungs (20%), with the remaining 10% occurring in other organs, such as the kidneys, spleen, brain, heart, and bones. The annual incidence of CE is estimated at 188,000 new cases. This results in an associated burden of 184,000 disability-adjusted life years (DALYs), highlighting the significant impact on global health [4].

The World Health Organization Informal Working Groups on Echinococcosis (WHO-IWGE) classification provides the basis for basically choosing four treatment and management options for CE: surgery, percutaneous sterilization, anti-parasitic treatment with benzimidazoles, and observation (watch and wait) for inactive, clinically silent cysts. Surgical intervention for CE requires highly skilled surgeons, an experienced expert team, and advanced medical facilities. To date, the WHO recommends only albendazole and mebendazole as anti-parasitic drug therapeutic agents for CE patients [5]. However, these benzimidazoles operate parasitostatically rather than parasitocidally and require long-term medication for alveolar *echinococcosis* [6]. A retrospective study indicated that the effectiveness of albendazole in treating CE varied, with recovery rates between 11.8% and 35.2%, and about 40% of patients did not respond favorably to the medication [7, 8]. Moreover, long-term albendazole treatment can lead to serious adverse effects [9]. The limited and inefficacious nature of current drug therapy options underscores the need for the development of novel and effective drugs for CE.

Previous studies assessed the viability of PSCs and metacestodes through visual observation using an inverted optical microscope [10], trypan blue staining [11], or a PSC motility assay [12]. Previous in vitro drug screening methods for *E. granulosus s.l.* have been focused on demonstrating direct protoscolicidal effects, mainly via the eosin exclusion test [13, 14]. However, *E. granulosus* PSCs are not the disease-causing stage of the parasite, and the eosin exclusion test is based on a low-throughput subjective evaluation of eosin staining intensity by light microscopy. The morbidity of CE is caused by the growth of metacestode cysts that cause compression of affected organs [15]. Therefore, drug screening should primarily focus on the metacestode cyst stage. However, given that second infection and post-surgery treatment to limit and kill PSCs often occur, protoscolicides and metocetodicides are needed. If a compound can be identified active on both PSC and metacestode cyst, that will be ideal for treating cystic echinococcosis. The criteria for evaluating dead cysts encompass a visual inspection to identify loss of turgidity and collapse of germinal layers, which appear conspicuously detached from the laminated layer [10]. However, the methods suitable for high-throughput screening still need further development. Image-based processing represents a promising technique for high-throughput screening [16]. High-content imaging has emerged as a valuable alternative to traditional assays [17]. For example, King et al. developed a new image-based, high-throughput, and high-content assay for testing natural products (purified compounds and extracts) against the human parasite *Trichomonas vaginalis*. The present study utilized high-content screening as an approach to screening for anti-echinococcal inhibitors.

Drug repurposing or repositioning to identify new therapeutic applications for “old” drugs, including FDA-approved drugs and drugs in clinical or preclinical stages [18], is an attractive strategy that can lead to the fast-track discovery of treatments for many diseases and in particular neglected tropical diseases. Our previous study employed a drug repurposing strategy to identify new therapeutic agents against echinococcal cysts. We repurposed pyronaridine, an approved antimalarial drug, for the treatment of CE. Following a three-dose intraperitoneal regimen (57 mg/kg, once a day for 3 days), pyronaridine caused 100% cyst mortality [19]. In line with this, the Repurposing, Focused Rescue, and Accelerated Medchem (ReFRAME) initiative was launched by Calibr at Scripps Research. The ReFRAME library includes about 12,000 valuable molecules collected from three resources—Clarivate Integrity, GVK Excelra GoStar, and Citeline Pharmaprojects—to expedite drug discovery [20]. It consists of FDA-approved or registered drugs (approximately 35%), investigational new drugs across various stages of clinical development (approximately 58%), and preclinical compounds (nearly 3%) with existing safety data (repeat dose efficacy or toxicity studies) [20]. Therefore, a majority of the library comprises unapproved therapeutic agents, presenting prospects for repurposing. The molecular targets for most of the compounds contained within the LOPAC1280 and SelleckChem libraries are well documented. The Sigma LOPAC1280 library comprises 1280 pharmacologically active compounds with known mechanisms. The content of the LOPAC1280 library reflects the most commonly screened targets in the drug discovery community. The SelleckChem library comprises 2745 molecules; approximately 1100 are FDA-approved, ~ 50 have been launched commercially, and ~ 230 are undergoing clinical trials, while the remainder are in preclinical stages of development.

In this study, we screened the ReFRAME, LOPAC1280, and SelleckChem libraries to identify licensed drugs with novel anti-echinococcal activity and efficacy to suppress the viability of PSCs and cysts of *E. granulosus*. To facilitate the application of the ReFRAME library to screen for anti-echinococcal compounds, we established a novel assay for PSCs based on a propidium iodide (PI) staining method and high-content screening (HCS) technology. The compounds identified were applied in an ex vivo experiment to characterize their efficacy on hydatid cysts.

## Methods

### In vitro culture and drug efficacy assessment of *E. granulosus* PSCs

PSCs were aspirated from echinococcal cysts isolated from naturally infected sheep livers collected from an abattoir in Urumqi, Xinjiang Uyghur Autonomous Region, China. The in vitro culture of PSCs was carried out on the basis of a previously described method [21]. Briefly, the PSCs underwent enzymatic digestion using 1% pepsin in sterilized Hank’s buffer at a pH of 2.0 for 30 min at 37 °C to eliminate dead and immature PSCs. Subsequently, the PSCs were washed eight times with phosphate-buffered saline (PBS) at pH 7.2. To assess vitality, the parasites were stained with 0.1% methylene blue, and only batches demonstrating over 98% viability were utilized in further experiments. Briefly, a 0.1% methylene blue solution was added to each well. After 5 min, the PSCs were observed under an inverted microscope. Dead PSCs were stained blue, while the live PSCs remained colorless [19]. The purified PSCs were cultured in 1640 medium containing 20% fetal calf serum (FCS), 0.2% yeast extract, and 0.4% glucose at 37 °C in a humid atmosphere with 5% CO_2_, at a concentration of 5000 PSC/mL. Purified PSCs were cultured for 1 month.

For PSC treatment, a 96-well plate was set up with approximately 100 ± 50 PSCs in 100 μL of medium per well. The PSCs were cultured in RPMI 1640 medium (Invitrogen, San Diego, CA, USA) supplemented with 20% fetal calf serum (FCS, HyClone, Logan, UT, USA) and antibiotics (100 IU/mL penicillin and 100 μg/mL streptomycin; HyClone) at 37 °C in a humid atmosphere with 5% CO_2_. Compounds dissolved in dimethyl sulfoxide (DMSO) (stock solutions of 10 mM) were added to the wells, resulting in the appropriate final concentrations for the assay.

During the screening procedure, 500 nL of a drug solution (original concentration, 10 mM; final concentration, 50 μM) or pure DMSO (final concentration, 0.5%) for experimental controls was placed in each well. The treatment plates were incubated at 37 °C in a humid atmosphere with 5% CO_2_ for durations ranging from 24 h to 96 h. The PSCs were then exposed to 20 μg/mL PI for 15 min before acquiring images using HCS.

### Compound libraries

The repurposing library utilized in this study included a selection of compounds from the LOPAC1280 library (from Sigma-Aldrich, https://www.sigmaaldrich.com/HK/zh/product/sigma/lo1280), the SelleckChem Bioactive Compound library (from Selleck Chemicals LLC, http://www.selleckchem.com/screening/chemical-library.html), and the ReFRAME library (https://reframedb.org/). The compound stock solutions were prepared in DMSO at 10 mM and were stored at −80 °C.

### Fluorescent dye test

Live, dead, and total PSCs were stained and analyzed by a high-content Opera Phenix system; nine fluorescent dyes, including DiOC18(3) (DIO), YOYO-1, Hoechst 33,342, calcein, ethidium homodimer-1, SYBRSAFE, Fluorescein Diacetate (FDA), SYTO 9, and PI, were used to stain both live and dead PSCs (1:1) in 96-well plate. The use and concentration of dyes were in accordance with their respective product manual. After 15–20 min of incubation, the fluorescence was visualized by a Perkin-Elmer Opera Phenix™ high-content screening system.

### Image acquisition

Phenotypic screening was conducted using a Perkin-Elmer Opera Phenix™ high-content screening system. Images were captured using an automated Opera Phenix™ Nipkow confocal HCS system (Perkin-Elmer, Germany) equipped with a 10 × objective lens (NA 1.0; binning 1). For each well of a 96-well plate, nine fields were imaged, and for 384-well plates, a single field was imaged. Fluorescence imaging was employed utilizing the PI channel (excitation at 493 nm and emission at 636 nm) to detect dead PSCs, while a brightfield channel facilitated the visualization of total PSCs. Images of PSCs or cysts/microcysts were acquired per well using the specified settings, yielding numbers sufficient for quantification. The acquired images were visualized using Harmony software (version 4.6; Perkin-Elmer).

PSCs that showed structural damage were initially excluded from the analysis as they were deemed unrepresentative. In the primary screen, the hits causing high mortality of PSCs were detected by the naked eye because most PSCs in the wells were stained by PI. In the secondary and third dose–response tests, dead and total PSC numbers were counted by visual inspection and manual counting. The main outputs (per well) judged by visual inspection were (i) the number of PSCs; (ii) the number of PI-stained (dead) PSCs; and (iii) the percentage of dead to the total number of PSCs.

The following was used to calculate the death rate: death rate of PSCs (%) = 100 × number of dead PSCs/number of total PSCs. The equation for the MIC_90_ (minimum inhibitory concentration causing a 90% death of PSCs) calculation was as follows: death rate of PSCs = bottom + (top–bottom)/(1 + 10^((LogMIC_90_ – concentration of compound)*HillSlope)).

### Development of anti-PSC assays in 96-well and 384-well formats

For assay development in a 96-well format, 100 ± 50 PSCs per 100 μL were seeded in each well of 96-well plates. Degrasyn and DMSO were pre-dispensed into the wells of the plates to a final concentration of 50 μM and 0.5%, respectively, before adding the medium containing PSCs or cultured small cyst (CSC). After incubation for 3 days, the images of the PSCs or CSCs (nine fields per well) were acquired by an HCS system.

For assay development in a 384-well format, 40 ± 10 PSCs/30 μL were seeded in each well of 384-well plates. Degrasyn and DMSO were pre-dispensed into plates to a final concentration of 50 μM and 0.5%, respectively, before adding the medium containing PSCs or CSCs. After incubation for 3 days, the images of PSCs or cysts/CSCs (one field per well) were acquired by the HCS system.

### Positive drug selection

Degrasyn, tyrphostin 9, and albendazole were pre-dispensed into plates to a final concentration of 1, 10, and 50 μM, respectively, before adding the medium containing PSCs or CSCs. Then, the image screening system was used to detect the inhibition rate on PSC viability of each compound, with 0.5% DMSO serving as a negative control.

### Development of PSCs into CSCs

The in vitro* E. granulosus* culture progression was divided into two stages: an early stage in which the PSC culture was established by digesting echinococcal cysts isolated from naturally infected sheep livers, followed by a late stage with differentiation into cysts. CSCs were cultured from PSCs according to our previously described method [22]. Briefly, the PSCs were incubated at 37 °C in glass culture vessels in RPMI 1640 medium (Invitrogen, San Diego, CA) containing 20% (v/v) FCS (Invitrogen), 0.45% (w/v) yeast extract, 0.4% (w/v) glucose, 100 IU/mL penicillin, and 100 μg/mL streptomycin placed on a solid-phase base prepared by heating newborn calf serum at 75 °C for 45 min (Smyth, 1990). Approximately 100 PSCs/mL were cultured, and the liquid medium was replaced every 3 days. After 56 days of cultivation, about 10% of the PSCs had developed into cysts with a thin laminated layer. The external diameter of the cysts was 328.6 ± 48 μm, and they appeared to the naked eye as clear capsular cysts. The cysts were carefully aspirated from the culture flasks and separated from dead or undeveloped PSCs by Pasteur pipetting. CSCs included microcysts and cysts: the diameter of the microcysts ranged from 300 μm to 1 mm, and the diameters of the cysts were greater than 1 mm.

### In vitro cyst/microcyst culture and viability assay

CSCs were cultured from PSCs utilizing the method described in a previous section. The in vitro cultured cysts (10 ± 5/well) or microcysts (10 ± 5/well) were seeded in 96-well plates before adding drugs. For the cyst/microcyst viability assay, cysts or microcysts were cultured in one well of a 96-well plate. After 1 day of culture, drugs were added to each well at a final concentration of 10 μM. For the cyst viability assay, the cyst survival rate was determined every 24 h after incubation with drugs, while for the microcyst viability assay, the survival rate of microcysts was determined at 4 days after drug incubation. The following equation was used for the death rate: death rate of CSCs (%) = 100 × (number of dead CSCs/number of total CSCs).

### Animal experiment

All animals were purchased from Charles River Corporation and maintained under pathogen-free conditions with a 12-h dark/light cycle at a temperature of 22 ± 3 °C. The mice had access to food and water ad libitum. Female mice aged 6–8 weeks were used in all experiments. All mice were euthanized under deep anesthesia (100 mg/kg pentobarbital used for euthanasia) at the end of the experiment (10 weeks after inoculation) or when showing pain symptoms.

#### Ex vivo culture of *E. granulosus* metacestodes

BALB/c mice were inoculated with cultured microcysts following the methodology outlined in a previous study [22]; 10 weeks post-infection, the mice were euthanized, and cysts were harvested from the peritoneal cavity. The cysts were categorized as size-small cysts, with a diameter less than 5 mm and without PSCs, or large cysts, with a diameter greater than 5 mm and with PSCs. Subsequently, the cysts were incubated in bottles with RPMI 1640 culture medium enriched with 10% FCS and antibiotics.

#### Ex vivo cyst viability assay

To determine the effect of omaveloxolone (HY-12212, MCE) and niclosamide (HY-B0497, MCE) on isolated cyst viability ex vivo, cysts collected from the peritoneal cavity of infected mice were incubated with different concentrations of drugs (1, 5, and 20 μM) for 7 days. The mortality rate of the cysts was recorded each day according to the following equation: death rate of cyst/microcyst (%) = [number of dead (cysts and microcysts)/number of total (cysts and microcysts)].

### Statistical analysis

Data are presented as mean values and error bars indicate the SD from at least three independent experiments. One-way analysis of variance (ANOVA) statistical analyses were performed with a Tukey post hoc test using GraphPad 9.0 (Prism) statistical software.

## Results

### In vitro culture of *E. granulosus* PSCs and PSC viability determination

**Fig. 1:**
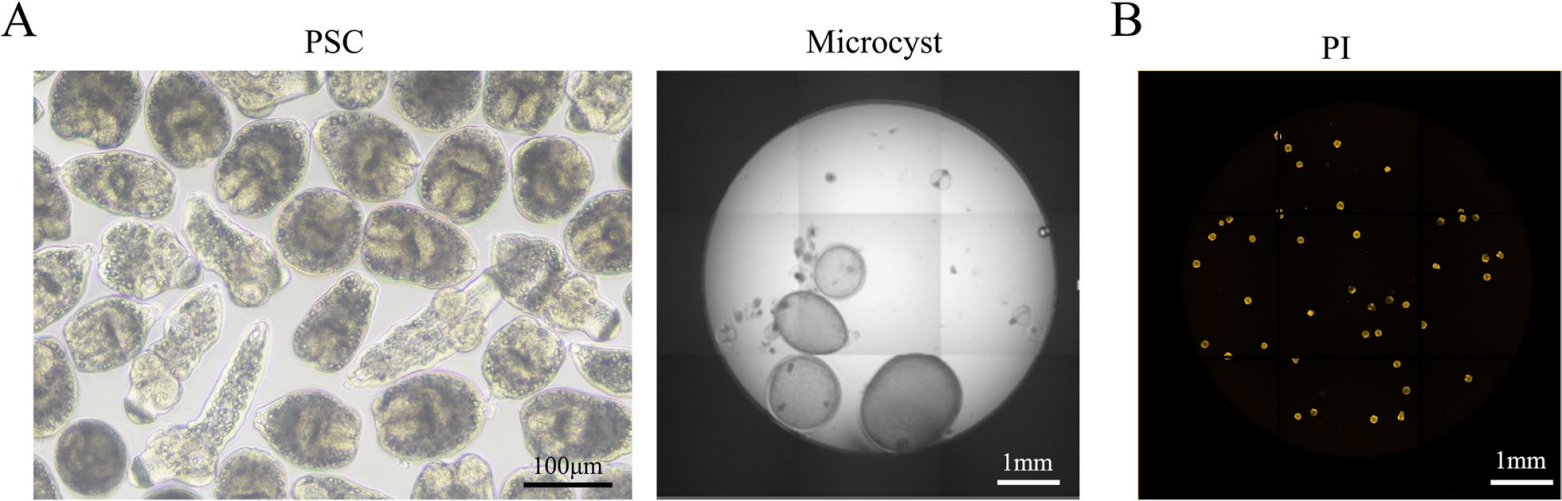
Image of cultured echinococcal PSCs and cultured small cysts (CSCs), and fluorescent images of dye-stained PSCs. **A** Echinococcal PSCs were aspirated from echinococcal cysts isolated from naturally infected sheep and cultured for 3 days. Live PSCs with locomotility were captured by an optical microscope (upper left). After 8–10 weeks of culturing, the PSCs developed into CSCs. Images of CSCs were captured by a Perkin-Elmer Opera Phenix™ system (upper right). **B** PI dye was co-incubated with a mixture of live and dead PSCs in a 96-well plate. The excited fluorescence was monitored by the Perkin Elmer Opera Phenix™ system. The images of PSCs with indicated dyes are displayed

To obtain live and mature PSCs, PSCs were aspirated from echinococcal cysts isolated from naturally infected sheep livers, followed by digestion, purification, and culturing. After culturing, the medium was changed every 3 days. After 6–8 weeks of in vitro culture, some CSCs with a complete laminated layer were observed. After 2 months, completely developed CSCs were observed (Fig. 1A).

Here, we attempted to utilize fluorescent dye to stain live, dead, and total PSCs and apply them to a high-content Opera Phenix system. As shown in Supplementary file 1, nine dyes, DIO, YOYO-1, Hoechst 33,342, calcein, ethidium homodimer-1, SYBRSAFE, FDA, SYTO 9, and PI, were used to stain the live and dead mixed PSCs. The results showed that except for PI, the dyes showed no specific binding efficiency with either live or dead PSCs (Fig. 1B, Supplementary file 1). PI showed a preference for dead PSC staining and showed little background noise, suggesting it could be used as a tool to reveal the status of PSCs after drug incubation. We then compared PI staining with eosin staining. Dead and live PSCs were mixed using different ratios and then stained by PI or eosin methylene blue. The results showed that the viability rates of PSCs by both staining methods were close (Supplementary file 1), suggesting that PI staining is a promising approach for identifying PSC viability.

### Development of anti-PSC drug screen assay in 96- and 384-well plate formats

**Fig. 2:**
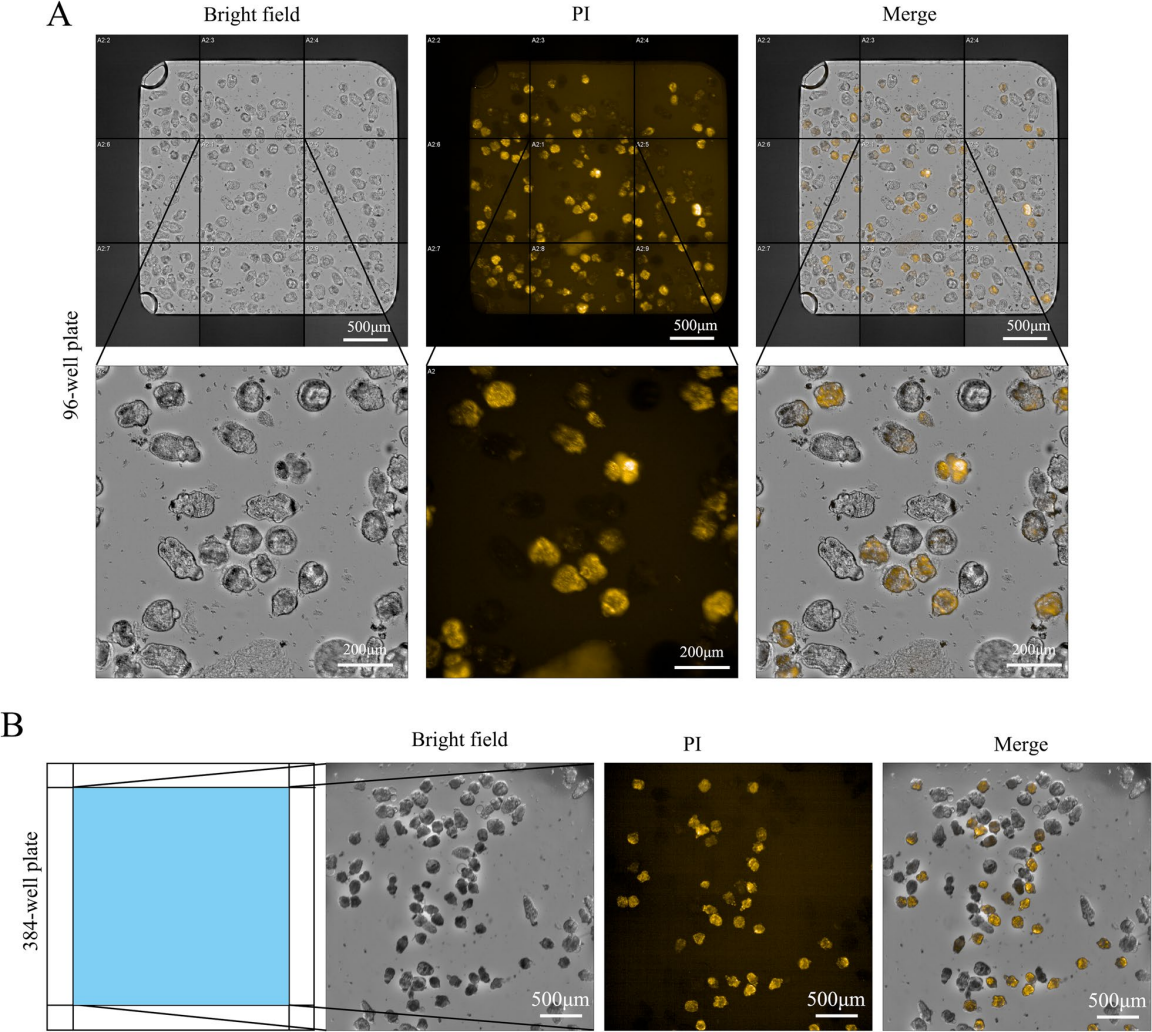
Image acquisition of PSCs in 96- and 384-well plates by the Opera system. **A** Sample image produced with the Opera system showing the effects of fixing and staining echinococcal PSCs with 2.5 μM propidium iodide (PI) in a 96-well plate for 15 min. PI, orange fluorescence. **B** Sample image produced with the Opera system showing the effects of fixing and staining echinococcal PSCs with 2.5 μM PI in a 384-well plate for 15 min. PI, orange fluorescence

Next, we attempted to establish an HCS assay for the reliable quantification of PI-stained dead PSCs and total PSCs using the Opera Phenix system. Thermal treatment and ethanol incubation were used to kill PSCs, and then the dead PSCs were mixed with live ones. Image acquisition was performed using the Opera Phenix HCS system (Fig. 2A). Bright field microscopy was used to detect total PSCs in one well of the 96-well plate, while PI staining showed dead PSCs in the images acquired with the HCS system. For the 96-well plates, nine fields were acquired per well (Fig. 2A), while for the 384-well plate, one field was obtained per well (Fig. 2B). The ratio of dead to total PSCs was determined primarily by the detection of PI-stained PSCs and total PSCs by visual inspection. PSCs that showed structural damage were excluded from the analysis as they were deemed unrepresentative.

**Fig. 3:**
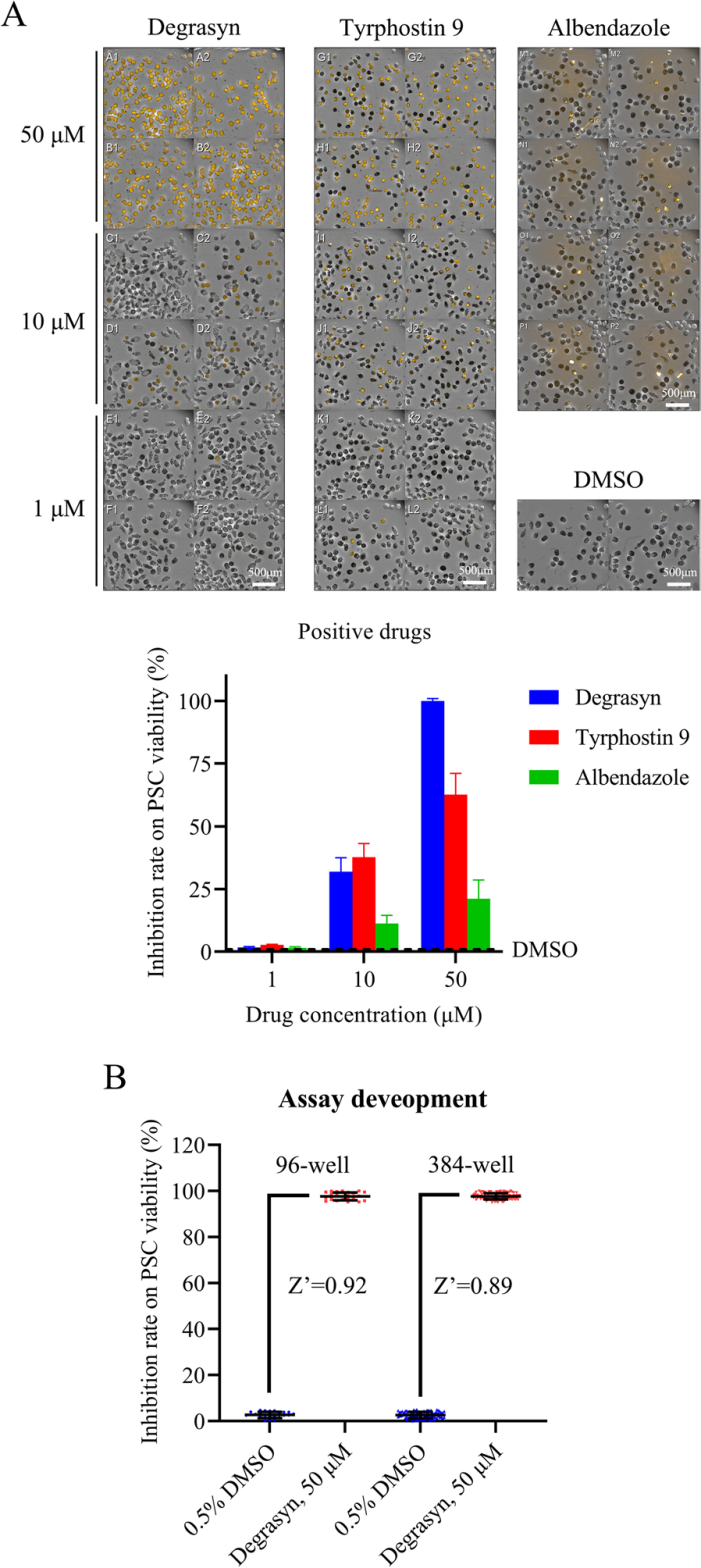
Selection of positive drugs using the HCS methodology. **A** A total of 100 ± 50 PSCs were seeded onto a 96-well plate. On day 1 post-culture, degrasyn, tyrphostin 9, and albendazole at different doses (1, 10, and 50 μM) were added to the medium. On day 3 post-treatment, dead PSCs and live PSCs were imaged by the Opera system. **B** Result of Z prime between DMSO treatment group and postive drug degrasyn group in 96-well and 384-well format

Prior to screening the anti-echinococcal drugs, we first attempted to choose a positive control drug with sufficient efficiency to kill the parasite. Degrasyn, tyrphostin 9, and the well-documented anti-enchinococcal drug albendazole were applied to the PCSs at concentrations of 1, 10, and 50 μM, while 0.1% DMSO served as a negative control. The PCSs were then analyzed by the image screening system. The results indicated that the survival rate of PSCs reached 0% after treatment with 50 μM degrasyn for 3 days. Lower concentrations of degrasyn, tyrphostin 9, and albendazole displayed no effect on the survival rate of echinococcal PSCs (Fig. 3A, B). Therefore, 50 μM degrasyn was chosen as the positive control.

**Fig. 4:**
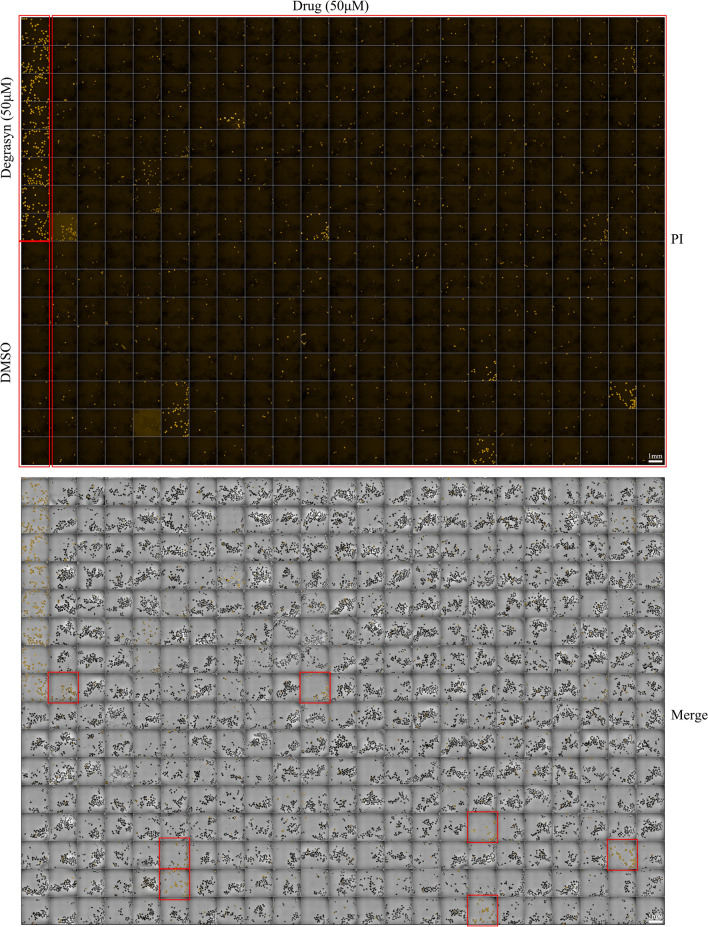
Representative images of the first stage of HCS. PSCs (50–150/well) were seeded onto 96-well plates. On day 1 post-culture, the positive control degrasyn (50 μM) was added to the medium. Compounds were prepared at a concentration of 50 μM in 96-well plates. After a 3-day incubation, PI dye was added to the wells at a final concentration of 2.5 μM. Dead PSCs and live PSCs were imaged by the Opera system

The next step was scaling up by performing the primary HCS in a 384-well plate (Fig. 4). In PI-stained images, a high lethality of the compound was directly observed and identified by the naked eye through visual inspection (50 μM degrasyn groups in Fig. 4). In the merged picture, the lethality rate can be counted and calculated. Here, we show an example of the high-content images, in which it was observed that 50 μM degrasyn caused complete mortality of echinococcal PSCs, whereas the vehicle control (0.5% DMSO) did not affect the survival rate of PSCs. The wells with 100% dead PSCs were easily observed, as nearly all PSCs displayed as yellow in the merged pictures. The wells were marked, and recorded (Fig. 4).

### High-content screening for an anti-echinococcal drug

**Fig. 5:**
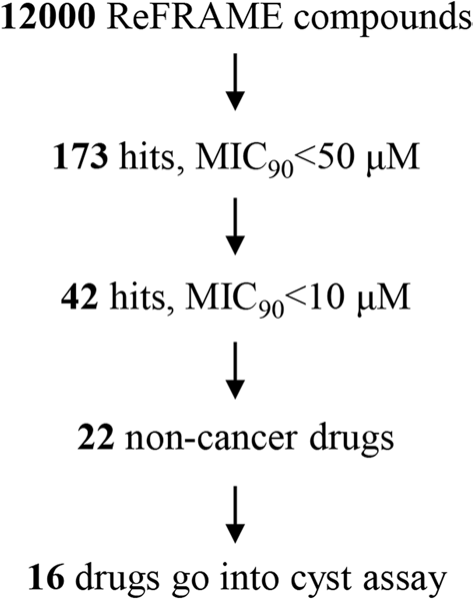
Primary screening and secondary confirmation of the ReFRAME library. The rationale for the 16 repurposed compounds selected from the primary and secondary screens

Next, we applied the HTS method using the ReFRAME, LOPAC, and Selleck libraries. Of the overall 12,000 ReFRAME compounds tested, 173 hits were identified in a primary screen (survival rate of PSCs < 90% at 50 μM), 20 hits were identified from the LOPAC library, and 39 hits were identified from the Selleck library. (Because some LOPAC and Selleck hits overlapped with ReFRAME and others failed in the following experiment, they were not described in the following results.) These compounds inhibited the viability of echinococcal PSCs with inhibition levels greater than 90% at 50 μM, similar to or better than the inhibition rate caused by 50 μM degrasyn (Fig. 5, Supplementary files 2, 3, 4). The 173 hits were distributed among cancer, psychoactive, inflammatory, microbial, cardiovascular, hypertensive, parasitic, and other targets and diseases (Table 1).

**Table 1:** Disease category division of the primary stage hits

Disease category	Hits
Cancer	52
Psychoactive drugs	29
Inflammation	16
Anti-microbial	24
Cardiovascular	11
Hypertension	7
Anti-parasite	7
Others	27
Total	173

The hits were then subjected to a second stage of testing over a range of several concentrations (0.5, 2, 10, and 50 μM). In a dose–response test, one hit was found to possess the highest inhibitory potency, causing over a 90% death rate of PSCs at a concentration of 0.5 μM, and three hits inhibited PSC viability over 90% at concentrations between 0.5 and 2 μM, whereas 38 hits inhibited PSC viability over 90% at concentrations between 2 and 10 μM (Fig. 5, Table 2, Supplementary file 2).

**Table 2:** Disease category division of the primary stage hits

Potency	Hits
MIC_90_ < 0.5 μM	1
MIC_90_ 0.5–2 μM	3
MIC_90_ 2–10 μM	38
MIC_90_ 10–50 μM	131
Total	173

**Fig. 6:**
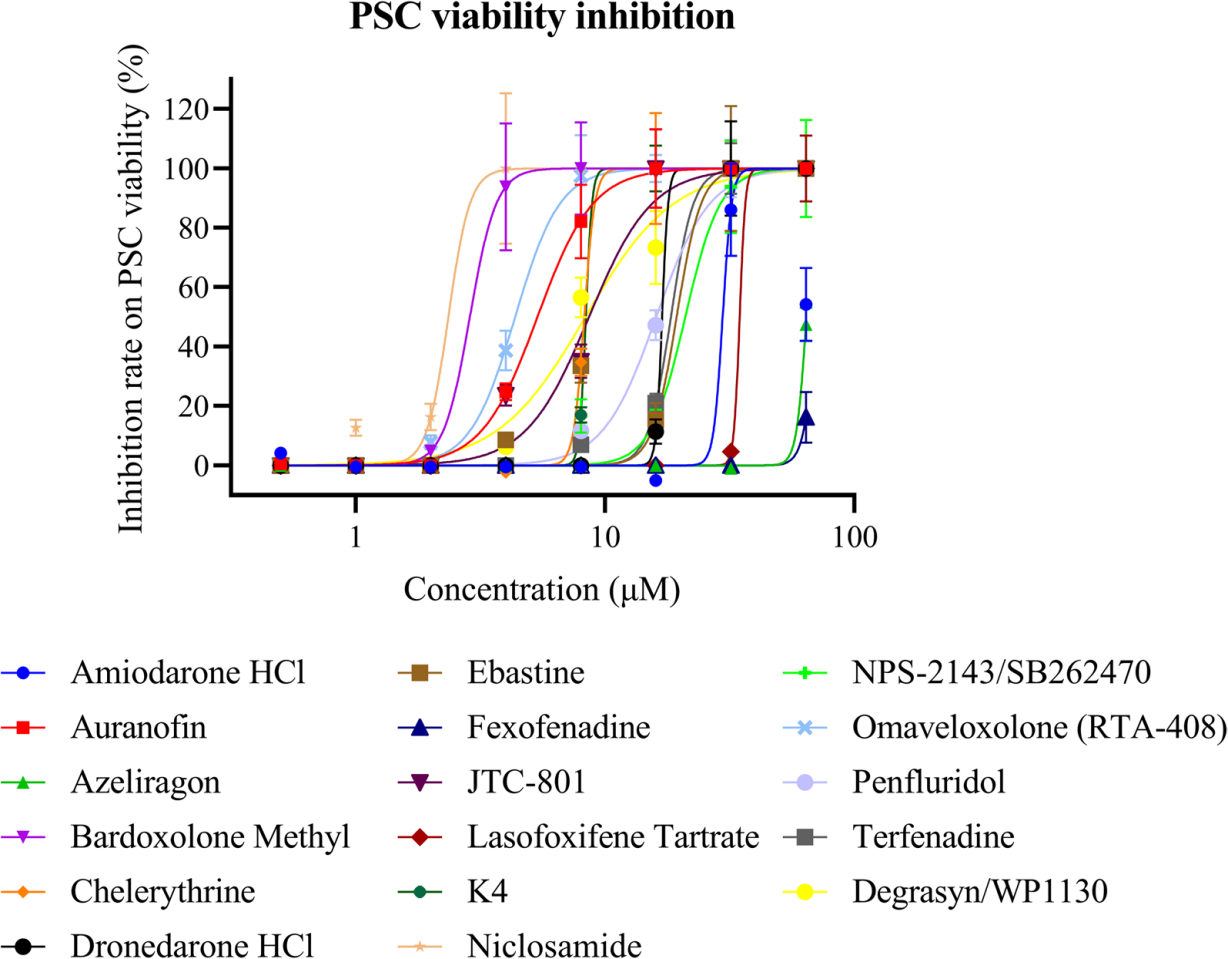
Third confirmation of the anti-echinococcal efficiency of the 16 hits. After 2 rounds of screening, the 16 repurposed compounds were selected for further dose-dependent testing. The 16 compounds with various concentrations (0.5, 1, 2, 4, 8, 16, 32, and 64 μM) were added to wells seeded with echinococcal PSCs. On day 3 post-treatment, the death rate of PSCs was calculated. Sigmoidal concentration–response curves were generated by Prism GraphPad 7.0

A third round of testing with a range of eight concentrations (0.5, 1, 2, 4, 8, 16, 32, and 64 μM) was performed on 22 hits from the total hits of the last dose response test (excluding cancer drugs); 16 of them retained potent activity and produced sigmoidal concentration–response curves with MIC_90_ values indicative of their effectiveness (Figs. 5, 6, Table 3, Supplementary file 5). A total of 16 compounds showed superior effects compared with the positive control degrasyn in the dose response assay, including niclosamide, bardoxolone, omaveloxolone (RTA-408), auranofin, and JTC-801. Among them, we identified three new compounds, niclosamide, bardoxolone, and omaveloxolone, with MIC_90_ activities of 2.9, 3.8, and 6.9 μM, respectively.

**Table 3:** Summary of the information on repurposed drugs and results of secondary confirmation on echinococcal PSCs

Name	Mechanism of action	Category	Clinical drug stage	MIC_90_, μM	Cytotoxicity in HepG2, CC_50_, μM	
Niclosamide	Autophagy inducer	Anti-parasite	Approved	2.9	6.14	
Bardoxolone methyl	Nuclear factor kappa B Inhibitor	Inflammation	Phase III	3.8	8.13	
Omaveloxolone	Nuclear factor kappa B inhibitor	Inflammation	Phase II/III	6.9	9.33	
Vitamin K4	Nutritional supplement, vitamin K	Vitamin K deficiency	Approved	8.9	> 100	
Chelerythrine	Protein kinase C inhibitor	Antimicrobial	Phase I	9.2	32.69	
Auranofin	Thioredoxin reductase inhibitor	Inflammation	Approved	9.4	13.42	
JTC-801	Opioid receptor-like 1 antagonist	Painkiller	Phase II	16.4	7.43	
Dronedarone HCl	Alpha adrenoreceptor antagonist	Cardiovascular	Approved	18	15.5	
Terfenadine	Histamine H1 receptor antagonist	Allergy	Phase III	23.1	7.31	
Ebastine	Histamine H1 receptor antagonists	Allergy	Approved	24.8	15.3	
Penfluridol	Dopamine receptor antagonist	Psychoactive drug	Approved	27.6	8.63	
NPS-2143	Calcium receptor antagonist	Hypertension	Phase I	30	15.26	
Azeliragon	RAGE receptor antagonist	Psychoactive drugs	Phase III	71	28.91	
Lasofoxifene	Estrogen receptor modulator	Osteoporosis	Approved	ND	ND	
Fexofenadine	Antihistamine	Allergy symptoms		ND	ND	

ND, not detected

### Further validation of active drugs in the cyst and microcyst viability assay

**Fig. 7:**
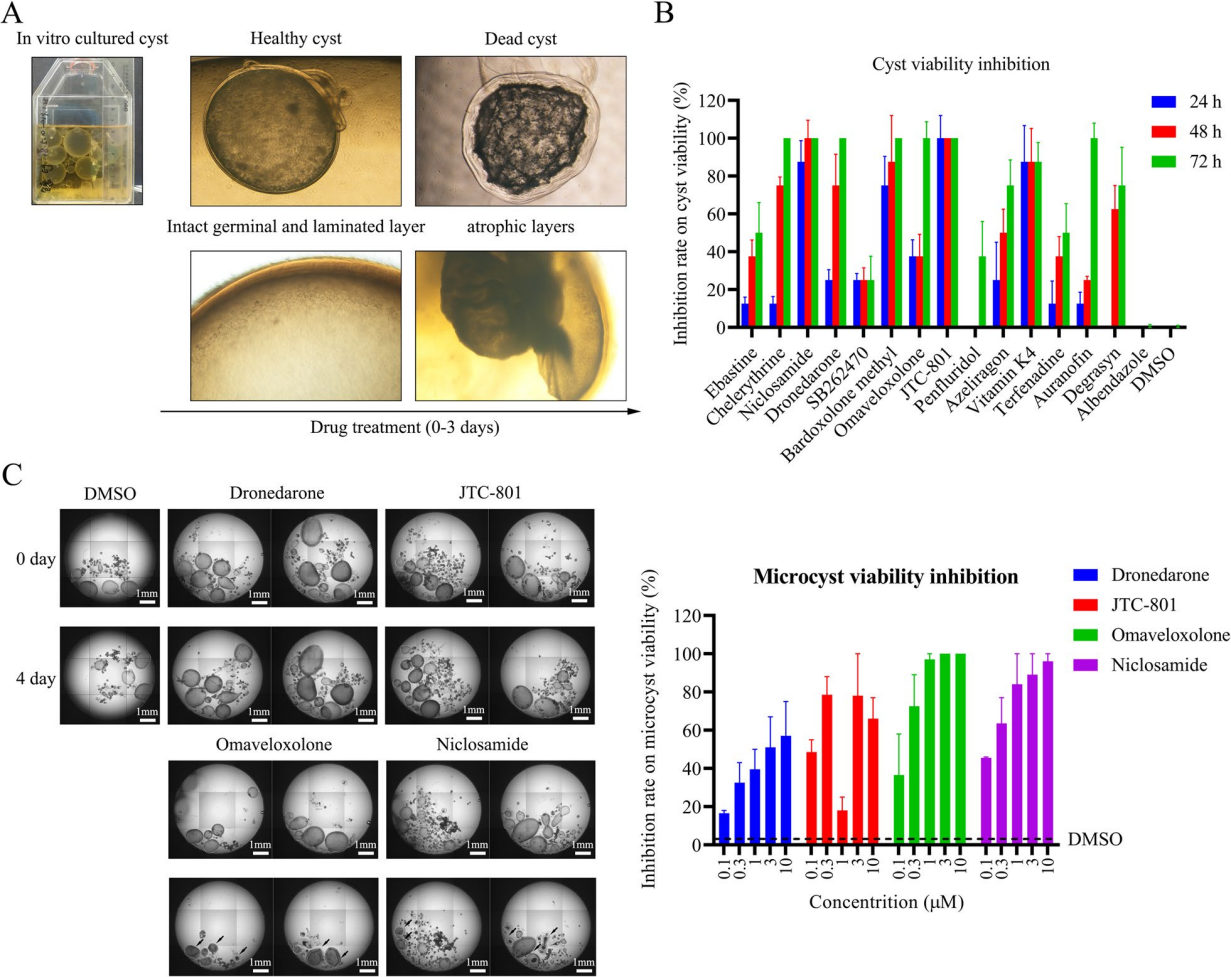
Effects of hits on the CSCs in vitro. **A** Cysts with diameters (> 1 mm) developed from PSCs cultured for more than half a year. The healthy cysts and dead cysts were distinguished by impaired germinal layers. **B** Effects of the 16 hits on the inhibitory rate of cysts (> 1 mm) in vitro. Cysts (*n* = 3–5/well) were seeded onto a 96-well plate. The dead cysts and healthy cysts were counted by visual inspection of deconstructed layers. **C** Effect of niclosamide, omaveloxolone, JTC-801, and dronedarone on the inhibitory rate of microcysts (< 1 mm) in vitro. Cysts (*n* = 10/well) were seeded onto a 96-well plate. The images were acquired by the Opera system. The dead cysts and healthy cysts were counted by visual inspection of deconstructed layers and PI staining

To further identify active compounds against echinococcal cysts from the 16 drugs, a cyst survival assay was performed using lab-cultured cysts and microcysts. First, cysts (*n* = 15 ± 5 per well) were seeded in a 96-well plate and then treated with the 16 drugs for 3 days. Degrasyn and albendazole served as positive controls. On day 3 post-drug treatment, it was observed that degrasyn treatment led to structural damage of the cyst, as evidenced by a collapsed outer acellular laminated layer and shrunken cyst core (Fig. 7A, Supplementary file 6). Among the 16 drugs, 7 of them reached a 100% inhibitory rate against the cysts on day 3 post-treatment, including chelerythrine, niclosamide, dronedarone, bardoxolone methyl, omaveloxolone, JTC-801, and auranofin (Fig. 7B). Next, we examined the efficiency of these seven compounds on the microcysts. Microcysts (*n* = 15 ± 5 per well) were seeded in a 96-well plate and incubated with one of the seven compounds for 4 days. At the end of incubation, the microcysts were analyzed by high-content scanning. Our data showed that omaveloxolone and niclosamide (10 μM) caused complete lethality of the microcysts, while dronedarone, JTC-801, chelerythrine (image not shown), and auranofin (image not shown) did not exert a significant inhibitory effect on microcyst viability (Fig. 7C).

### Effect of omaveloxolone and niclosamide in an ex vivo cyst viability assay

**Fig. 8:**
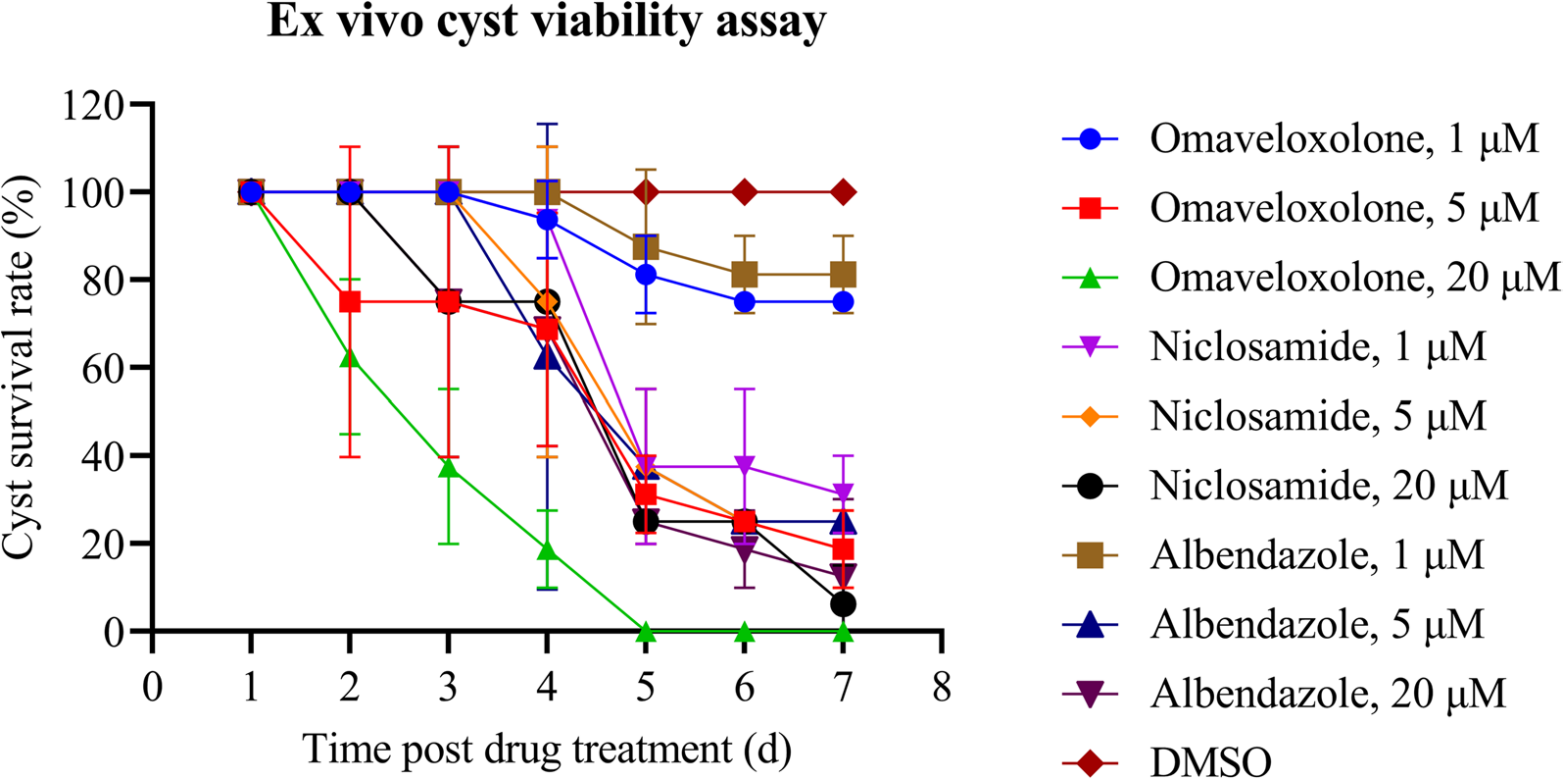
Effect of omaveloxolone and niclosamide on isolated cysts ex vivo. Cysts were collected from mice infected with microcysts. Omaveloxolone and niclosamide at different concentrations were incubated with the cysts for 1–7 days. The dead cysts and healthy cysts were counted by visual inspection of deconstructed layers

Considering the effectiveness of bardoxolone methyl, omaveloxolone, and niclosamide on both PSC and lab-cultured cysts/microcysts, as well as a similar mechanism of action of bardoxolone methyl and omaveloxolone, we further incubated these two compounds with *E. granulosus* cysts (collected from mice infected with microcysts) at concentrations ranging from 1 to 20 μM for 7 days. An obvious metacestodicidal effect was observed after treatment with omaveloxolone and niclosamide. Both compounds showed dose-dependent and time-dependent parasiticidal activity against the *E. granulosus* cysts ex vivo. The administration of 20 μM of omaveloxolone killed all cysts by day 5, whereas a 5 μM concentration resulted in a 50% reduction in cyst viability by day 2. In comparison, niclosamide demonstrated inferior efficacy in cyst elimination. Complete lethality was only observed in the 20 μM niclosamide treatment group on day 7. However, a high parasiticidal efficiency (approximately 80%) occurred at a low incubation concentration of niclosamide from day 5 (Fig. 8, Supplementary file 7). Collectively, we found that omaveloxolone was more effective against the cysts at a high concentration, while niclosamide was more effective against the cysts at a low dosage.

## Discussion

Given that cystic hydatid disease presents a considerable global health challenge, the urgent development of novel anti-parasitic agents is imperative. In this study, HCS technology was first utilized to establish a high-throughput method to screen for protoscolicidal compounds in the ReFRAME library, the Sigma-Aldrich LOPAC1280 library, and the SelleckChem library. In total, 173 hits were identified from the Reframe library, whereas 20 and 39 hits were identified from the LOPAC library and Selleck library, respectively, after primary screening. In the following two rounds of dose–response tests with PSCs and further tests in cultured cysts and microcysts, we identified a suitable selectivity index of at least seven compounds against *Echinococcus* PSCs and cysts in vitro. Furthermore, two molecules, niclosamide and omaveloxolone, had potent activity against *E. granulosus* cysts ex vivo.

To the best of our knowledge, this is the first attempt to introduce PI dye to stain dead *E. granulosus* PSCs for screening. PI is a fluorescent intercalating agent that can be used to stain cells and nucleic acids. PI binds DNA by intercalating between the bases with little or no sequence preference. PI is used as a DNA stain in flow cytometry to evaluate cell viability or DNA content in cell cycle analysis, or in microscopy to visualize the nucleus and other DNA-containing organelles. PI is not membrane permeable, and this property renders it useful to differentiate necrotic, apoptotic, and healthy cells based on membrane integrity [23]. In this study, live PSCs had intact membranes that blocked PI from entering the cell to stain nucleic acids. After PSCs were inhibited by thermal treatment or incubation with ethanol and protoscolicidal drugs, the cell membrane was damaged and allowed PI to permeate the membrane. We also compared PI staining with eosin staining, which is the gold standard for determining PSC viability. Dead and live PSCs were mixed using different ratios and then stained by PI or eosin methylene blue. The results showed that the viability rates of PSCs by both staining methods were very similar, suggesting that PI staining is a promising approach for identifying PSC viability. Z prime test also indicated that PI staining-based assay showed a good robustness.

In the present study, it was hypothesized that high-content imaging could provide a more sensitive method to assess the viability of individual PSCs. Nevertheless, this study identified a compatibility issue: live PSCs resisted staining by all tested dyes, rendering them undetectable by solely using imaging-based methodologies. To address this, our approach involved using a single fluorescence channel to label dead PSCs, greatly reducing assay complexity. This HCS technology applied to *E. granulosus* PSCs was found to be a useful alternative to traditional assays.

Another highlight of this study was the utilization of the ReFRAME library. Utilizing this library for screening may expedite the development of promising drugs against *E. granulosus* infections and facilitate public–private partnerships for drugs currently under active commercialization. Drug repurposing has expedited the drug research and development process in recent years, reducing costs by 40% [24, 25]. Notably, various studies utilizing the ReFRAME library have identified repurposed drugs with anti-parasitic properties, including those effective against *Candida auris* [26], *Cryptosporidium* VB-201 [20], *Trypanosoma cruzi* (348U87) [27], and *Entamoeba histolytica* (ponatinib) [28]. In this study, after two rounds of dose response tests, seven compounds tested in vitro against *Echinococcus* PSCs and cysts with a suitable selectivity index were identified; among them, niclosamide and omaveloxolone, with explicit potent in vitro activity, were also effective against *E. granulosus *ex vivo.

Currently, the most potent compounds described against *E. multilocularis* are MMV689480 (buparvaquone) and MMV671636 (ELQ-400), with MIC_50_ activities of 2.87 and 0.02 μM against *E. multilocularis* metacestodes cultured in vitro, respectively [29]. In the present study, we identified three new compounds, niclosamide, bardoxolone, and omaveloxolone, with a potency similar to that of buparvaquone, with MIC_90_ activities in *E. granulosus* of 2.9, 3.8, and 6.9 μM, respectively. In the secondary round of screening, we confirmed the anti-echinococcal PSC activity of one previously published compound (niclosamide [18]), whereas the other 15 compounds we discovered were novel.

From evaluating anti-echinococcal PSC and cyst activity, we identified three compounds, niclosamide, bardoxolone, and its derivative omaveloxolone, as active against both PSCs and cysts in vitro. Bardoxolone and omaveloxolone represent the second generation of synthetic oleanane triterpenoid compounds developed by Reata Pharmaceuticals. Preclinical studies have shown that omaveloxolone exhibits antioxidative and antiinflammatory effects [30, 31], as well as the capacity to enhance mitochondrial bioenergetics [32]. Currently, omaveloxolone is undergoing clinical trials for an array of diseases, such as Friedreich’s ataxia and mitochondrial myopathies, immuno-oncology applications, and the prevention of corneal endothelial cell loss after cataract surgery [33]. According to Kangussu-Marcolino et al., bardoxolone methyl showed a potent inhibitory effect on *N. fowleri* growth, achieving approximately 85% inhibition at 24 h and 97% after 48 h [34]. Haid et al. also applied repurposing screening to identify antivirals with broad-spectrum coronavirus activity. They found that bardoxolone and omaveloxolone acted as inhibitors of both alpha- and beta-coronaviruses [35]. Bardoxolone and omaveloxolone alleviated oxidative stress by activating Nrf2; however, oxidative stress was demonstrated to initiate early apoptotic processes in PSCs [36]. Hence, both compounds were thought to protect PSCs through antioxidant responses. However, we observed that bardoxolone and omaveloxolone treatment efficiently killed both PSCs and cysts/microcysts in vitro, suggesting that bardoxolone and omaveloxolone used other mechanisms or targets in *E. granulosus* to exert their function.

Niclosamide, an anthelmintic drug, demonstrates efficacy against the adult stages of multiple tapeworm species, such as in the treatment of diphyllobothriasis, hymenolepiasis, and taeniasis [37]. In addition, it has shown experimental promise addressing other conditions, including Parkinson’s disease, diabetes, a variety of viral and microbial infections, and several types of cancers [38, 39]. Lundström-Stadelmann used an in vitro screening cascade that included an assessment of the drug-induced physical damage of metacestodes, the impact on metacestode viability, and the viability of isolated parasite stem cells [18]. By utilizing this screening cascade, they showed that mefloquine, niclosamide, and MMV665807 (a salicylanilide-derivative of niclosamide) were active against *E. multilocularis* metacestodes in vitro. In mice that were infected either intraperitoneally with metacestodes or orally with eggs, oral treatment with either niclosamide or MMV665807 did not lead to a reduction of the metacestode burden compared with the standard treatment with albendazole. According to their hypothesis, niclosamide has a poor absorption rate in vivo, suggesting a predictable failure of niclosamide in an *E. granulosus*-infected animal model [18]. Kaethner et al. utilized four assays to assess drug activity against *E. multilocularis* and *E. granulosus s.s.* PSCs, metacestode vesicles, and germinal layer cells, including (i) a metacestode vesicle damage marker release assay, (ii) a metacestode vesicle viability assay, (iii) a germinal layer cell viability assay, and (iv) a PSCs motility assay. MMV665807 and niclosamide were active against the parasites of both species in all four assays [12]. The findings in both publications were consistent with our data. As validation for our assays, including an in vitro anti-PSC viability assay and in vitro and ex vivo cyst/microcyst viability assays, we also identified niclosamide as having anti-*E. granulosus* PSC and cyst activity.

One of the limitations of this study was the lack of data on an *E. granulosus*-infected animal model, given the limited experimental conditions for establishing an animal model of *E. granulosus*. Another disadvantage was that we did not introduce a computational hurdle in distinguishing live PSCs via brightfield microscopy. We endeavored to create an algorithm for the automated processing of the collected images of live PSCs to facilitate the analysis of the datasets produced by the image software. The algorithm’s development was hindered by two major obstacles: (i) the heterogeneity in the shape and size of live PSCs and (ii) their motility, which led to significant blurring of images, even at relatively fast exposure times.

## Conclusions

With the integration of HCS technology into the development and optimization of an HTS methodology, here we first described a novel screening method for repurposing compounds that inhibited *E. granulosus* PSC viability. Our results offer in vitro evidence of niclosamide and omaveloxolone effectiveness against *E. granulosus* that warrants further exploration for the treatment of this disease. This approach paves the way for identifying novel repurposing agents that may be utilized in future anti-parasitic strategies to combat infections caused by *E. granulosus*.

### Supplementary Information

Additional file 1: Table S1. Summary of dye test results on live and dead PSCs.

Additional file 2: Dataset S1. Summary of secondary hits from the ReFRAME library.

Additional file 3: Dataset S2. Summary of secondary hits from the Sigma-Aldrich LOPAC1280library.

Additional file 4: Dataset S3. Summary of secondary hits from the SelleckChem library.

Additional file 5: Dataset S4. PSC images of 16 drug treatment results in a dose–response assay.

Additional file 6: Figure S1. Drug efficacy in the in vitro cyst viability assay.

Additional file 7: Figure S2. Raw pictures of cysts in* ex vivo* cyst viablity study.

## Data Availability

Data are provided within the manuscript or supplementary information files.

## Abbreviations

CE: Cystic echinococcosis
*E. granulosus*: *Echinococcus* *granulosus* Sensu lato
HCS: High-content screening
HTS: High-throughput screening
PI: Propidium iodide
ReFRAME: Repurposing Focused Rescue and Accelerated Medchem
PSC: Protoscoleces
AE: Alveolar Echinococcosis
WHO: World Health Organization
PGI: Phosphoglucose isomerase
DMSO: Dimethyl sulfoxide
FCS: Fetal calf serum
