# Supplementary material for: Repurposing of a library for high-content screening of inhibitors against Echinococcus granulosus
Source: Parasit Vectors. 2024 Sep 3;17:373. doi: 10.1186/s13071-024-06456-6 (PMC11370232; doi:10.1186/s13071-024-06456-6)

**Supplementary file 1.** Summary of dye test results on the live or dead PSC and comparison between eosin and PI staining.


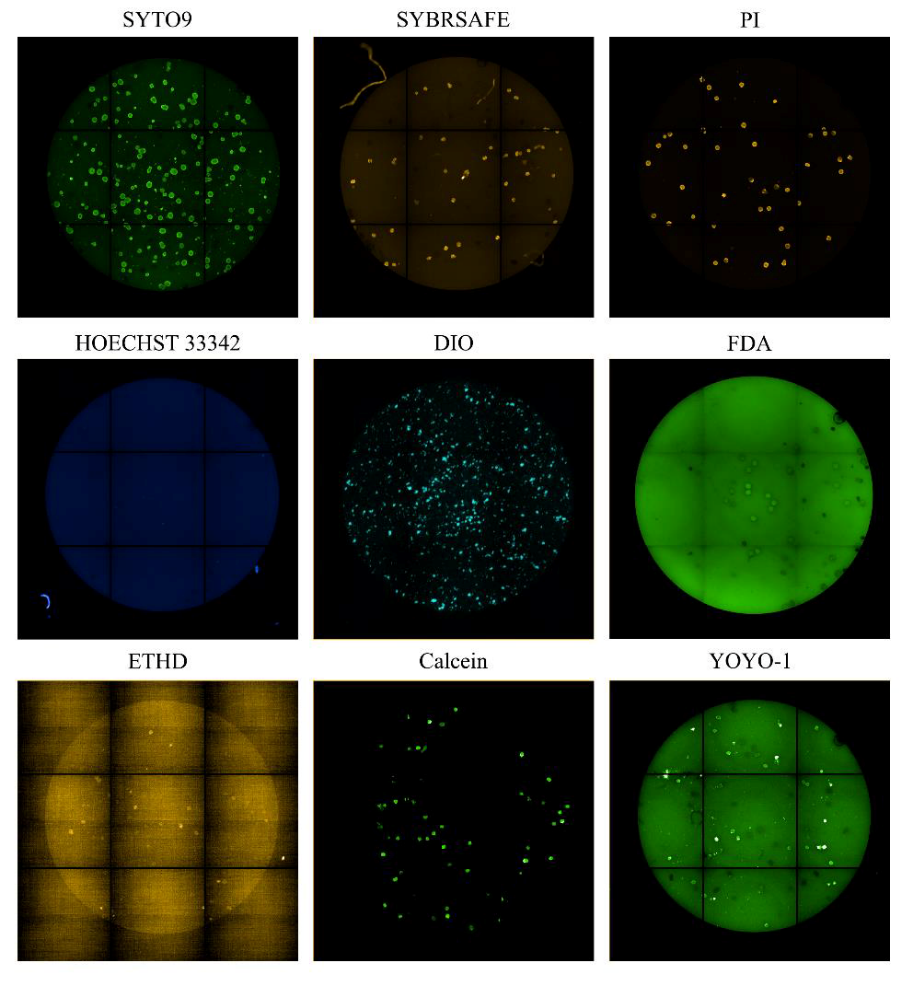


| Dye | Permeability | Target | Fluorescence | Result |
| --- | --- | --- | --- | --- |
| DIO;DIOC18(3) | N.A | Cell membrane | Green | Fail, no fluorescence |
| YOYO-1 | Permeant | Total cell DNA | Green | Fail, dead cell preferred, high background noise |
| HOECHST 33342 | Permeant | Total cell DNA | Blue | Fail, no fluorescence |
| Calcein | Permeant | Live cell enzyme | Green | Fail, only stain dead cell |
| Ethidium homodimer-1 | Non-permeant | Dead cell DNA | Red | Fail, weak fluorescence |
| SYBRSAFE | Permeant | Total cell DNA | Orange | Fail, only stain dead cell |
| FDA | Non-permeant | Live cell enzyme | Green | Fail, only stain dead cell and high background noise |
| SYTO 9 | Permeant | Total cell DNA | Green | Fail, dead cell preferred |
| PI | Non-permeant | Dead cell DNA | Orange | Applicable |


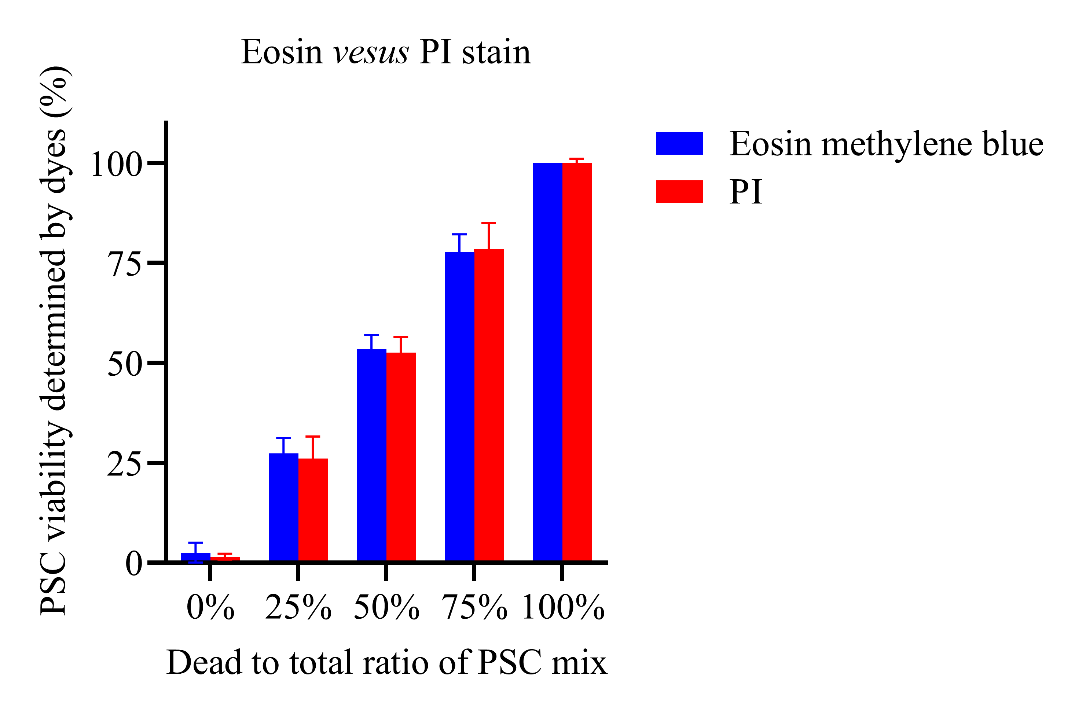

Supplement: Supplementary file 1 — Additional file 1: Table S1. Summary of dye test results on live and dead PSCs. [file 13071_2024_6456_MOESM1_ESM.docx]
