# Supplementary figures and images for: Repurposing of a library for high-content screening of inhibitors against Echinococcus granulosus
Source: Parasit Vectors. 2024 Sep 3;17:373. doi: 10.1186/s13071-024-06456-6 (PMC11370232; doi:10.1186/s13071-024-06456-6)

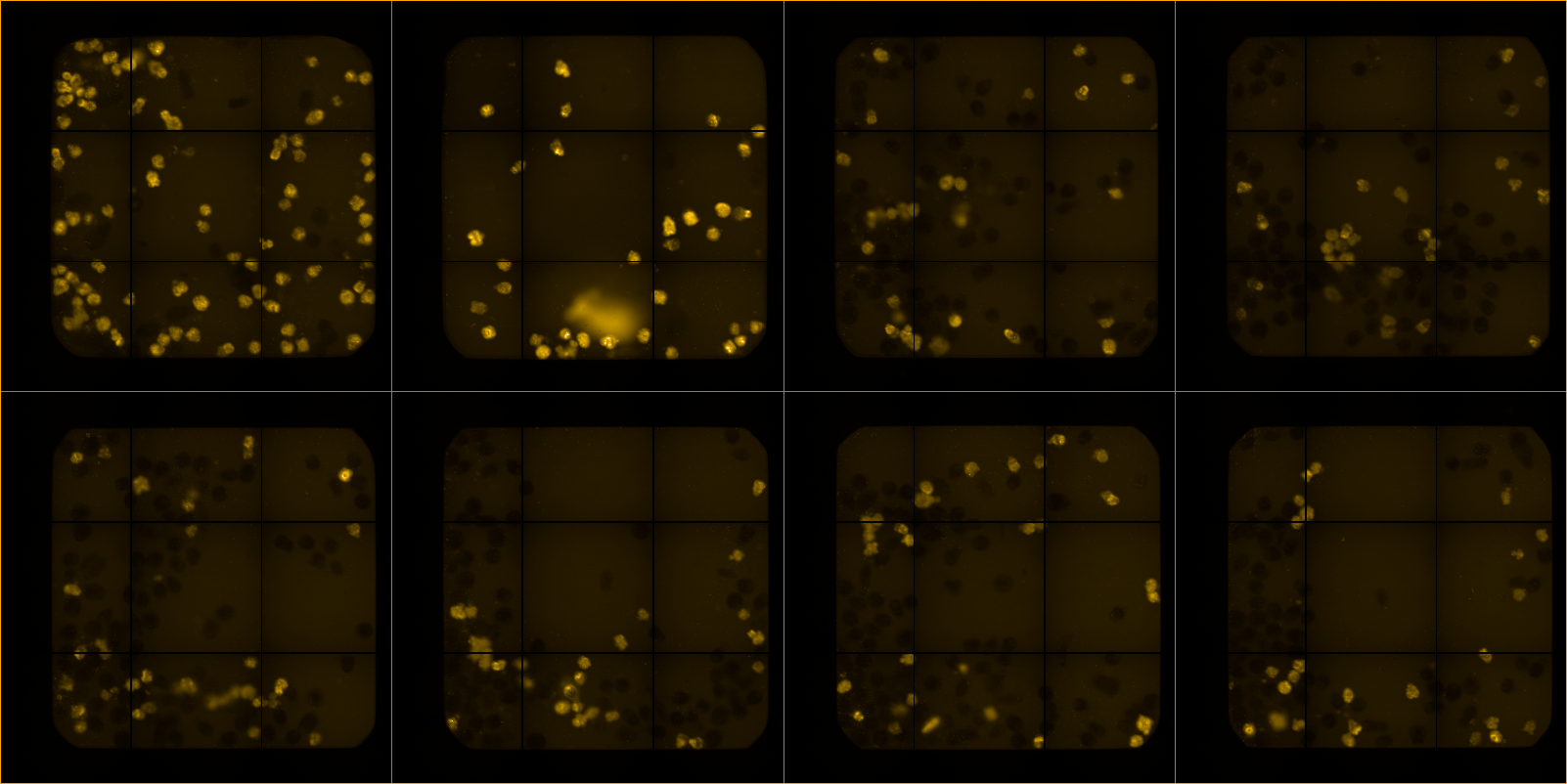

Supplement: Supplementary file 5 — Additional file 5: Dataset S4. PSC images of 16 drug treatment results in a dose–response assay. [file 13071_2024_6456_MOESM5_ESM.zip › Supplementary file 5/Aminodarone HCl-2.png]

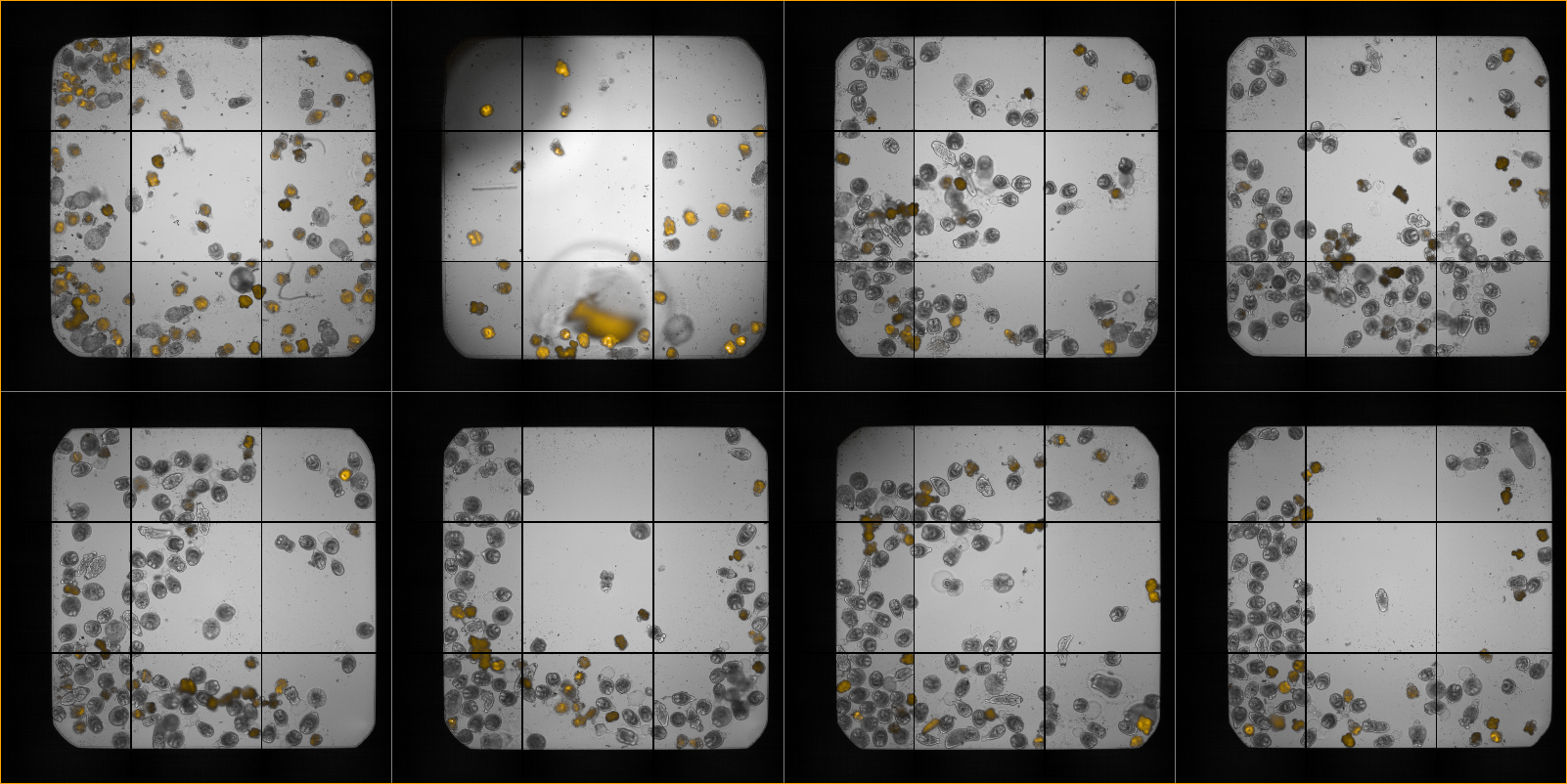

Supplement: Supplementary file 5 — Additional file 5: Dataset S4. PSC images of 16 drug treatment results in a dose–response assay. [file 13071_2024_6456_MOESM5_ESM.zip › Supplementary file 5/Aminodarone HCl.png]

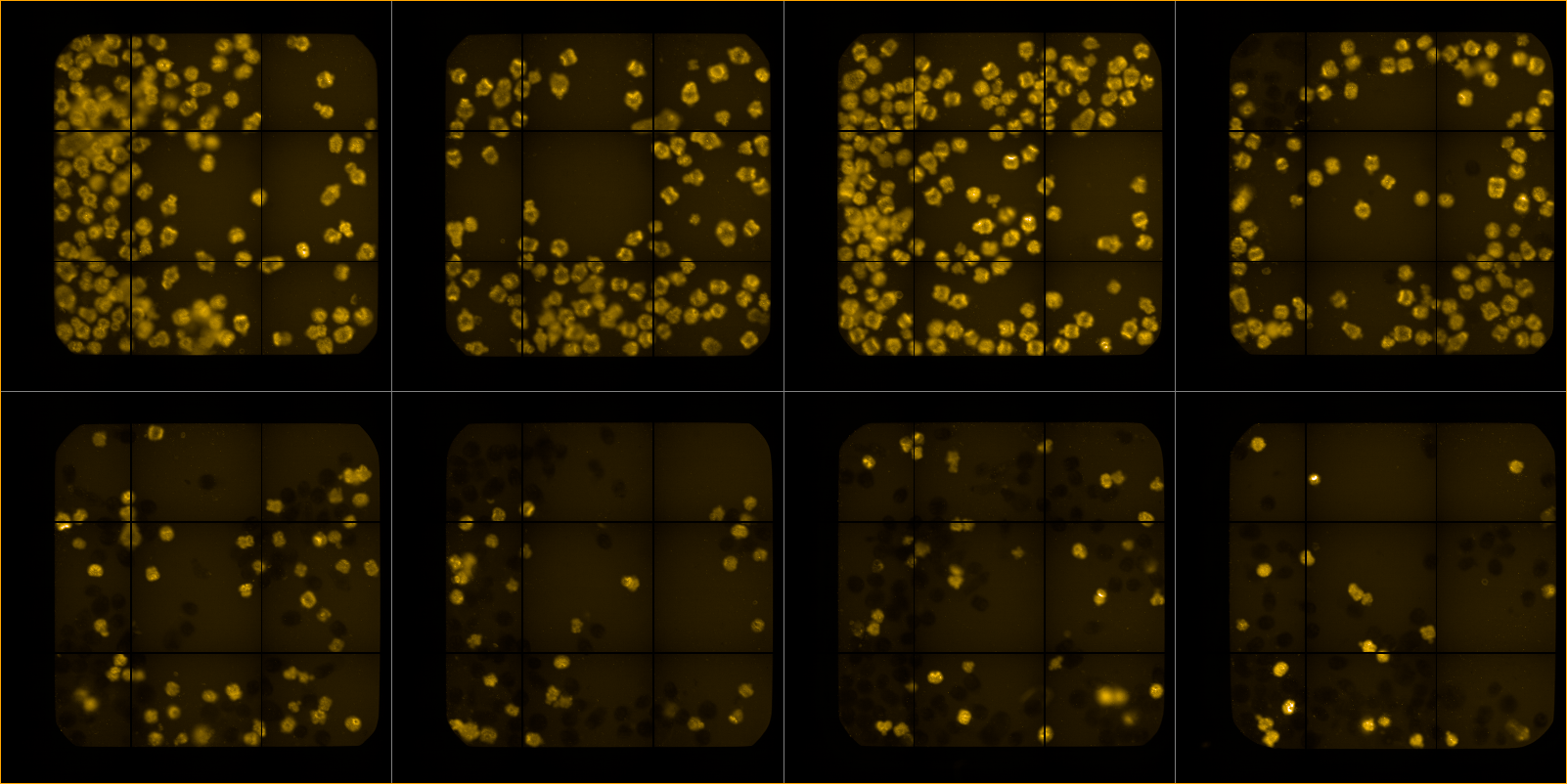

Supplement: Supplementary file 5 — Additional file 5: Dataset S4. PSC images of 16 drug treatment results in a dose–response assay. [file 13071_2024_6456_MOESM5_ESM.zip › Supplementary file 5/Auranofin-2.png]

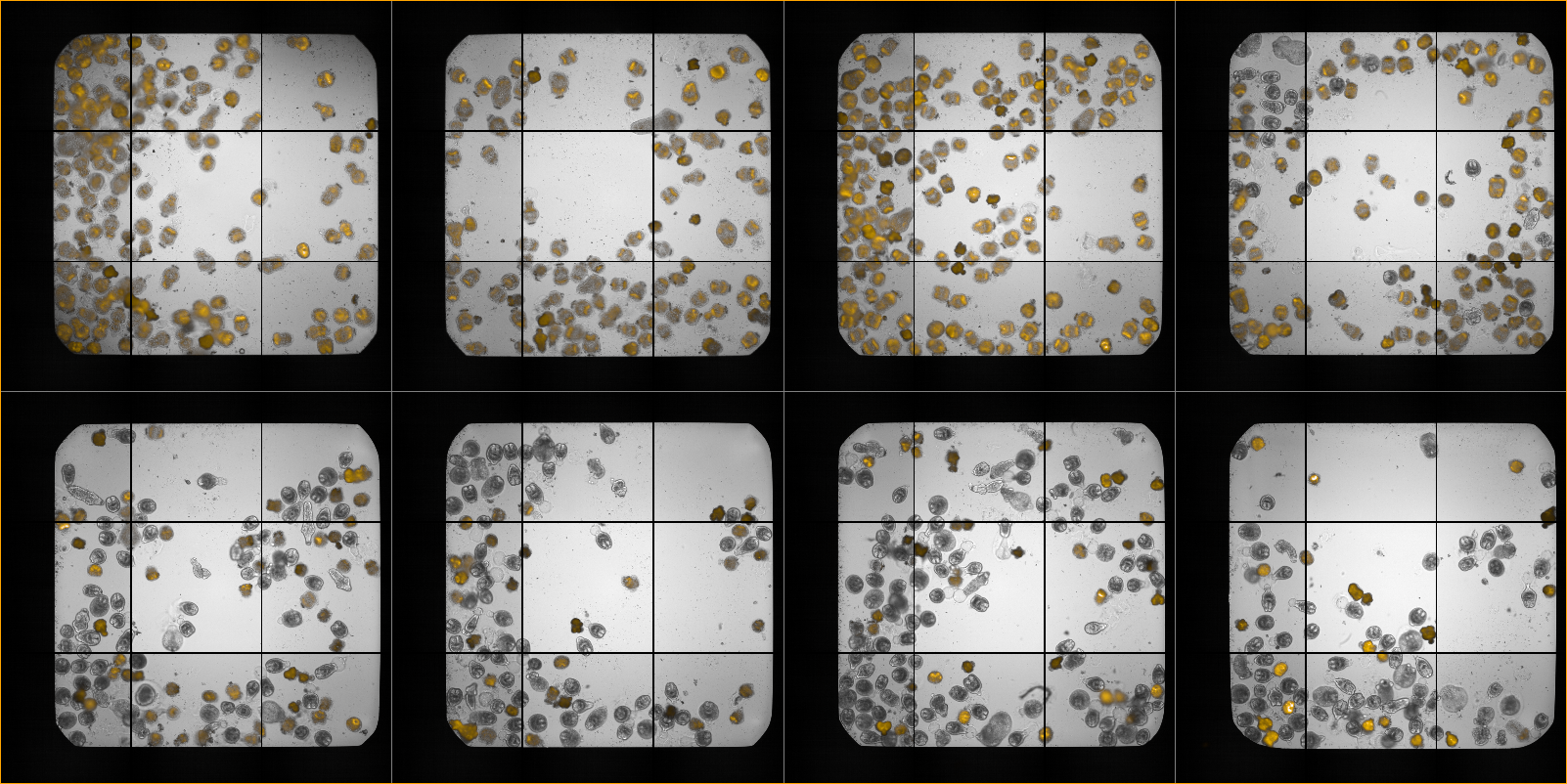

Supplement: Supplementary file 5 — Additional file 5: Dataset S4. PSC images of 16 drug treatment results in a dose–response assay. [file 13071_2024_6456_MOESM5_ESM.zip › Supplementary file 5/Auranofin.png]

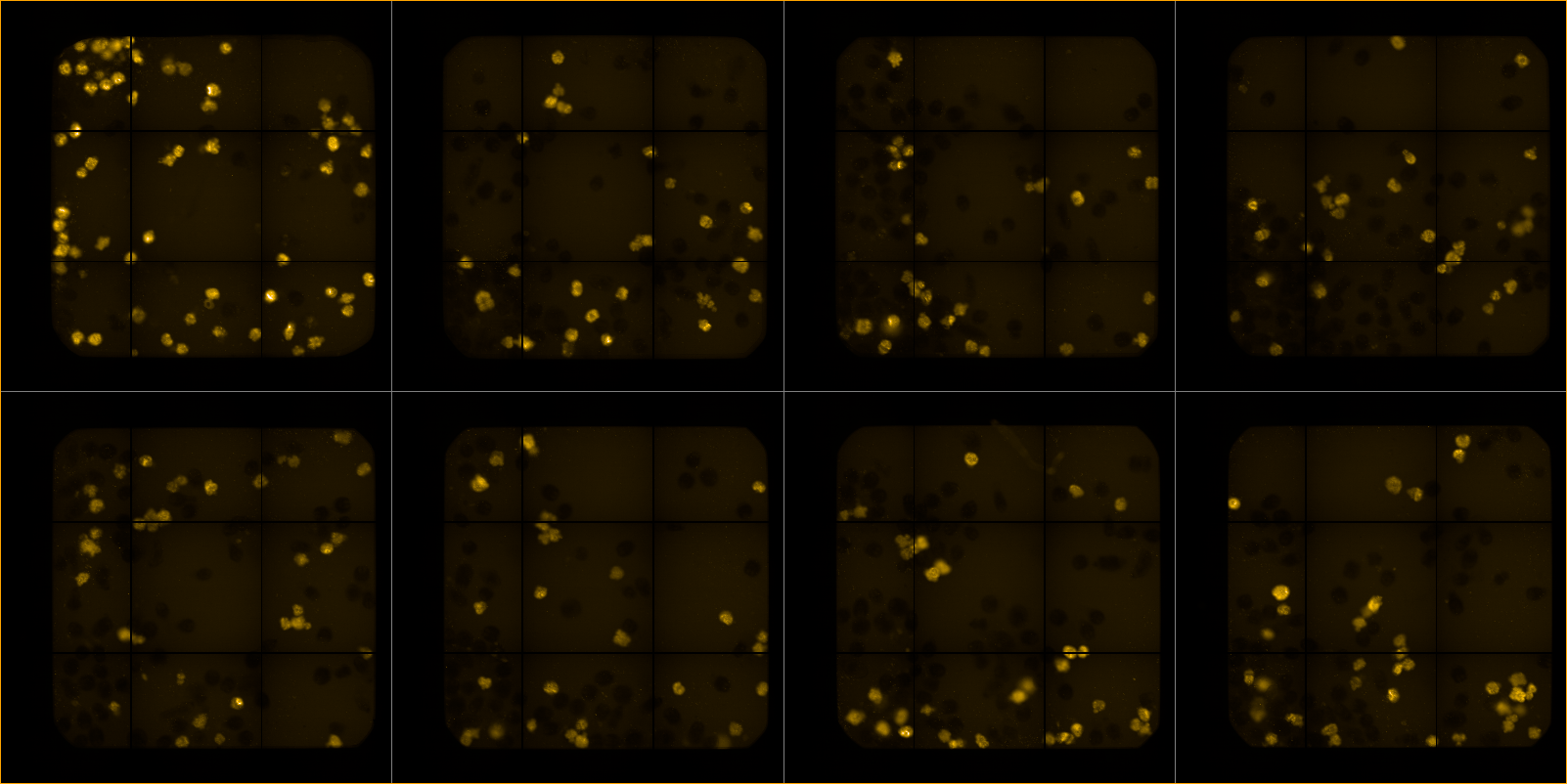

Supplement: Supplementary file 5 — Additional file 5: Dataset S4. PSC images of 16 drug treatment results in a dose–response assay. [file 13071_2024_6456_MOESM5_ESM.zip › Supplementary file 5/Azeliragon-2.png]

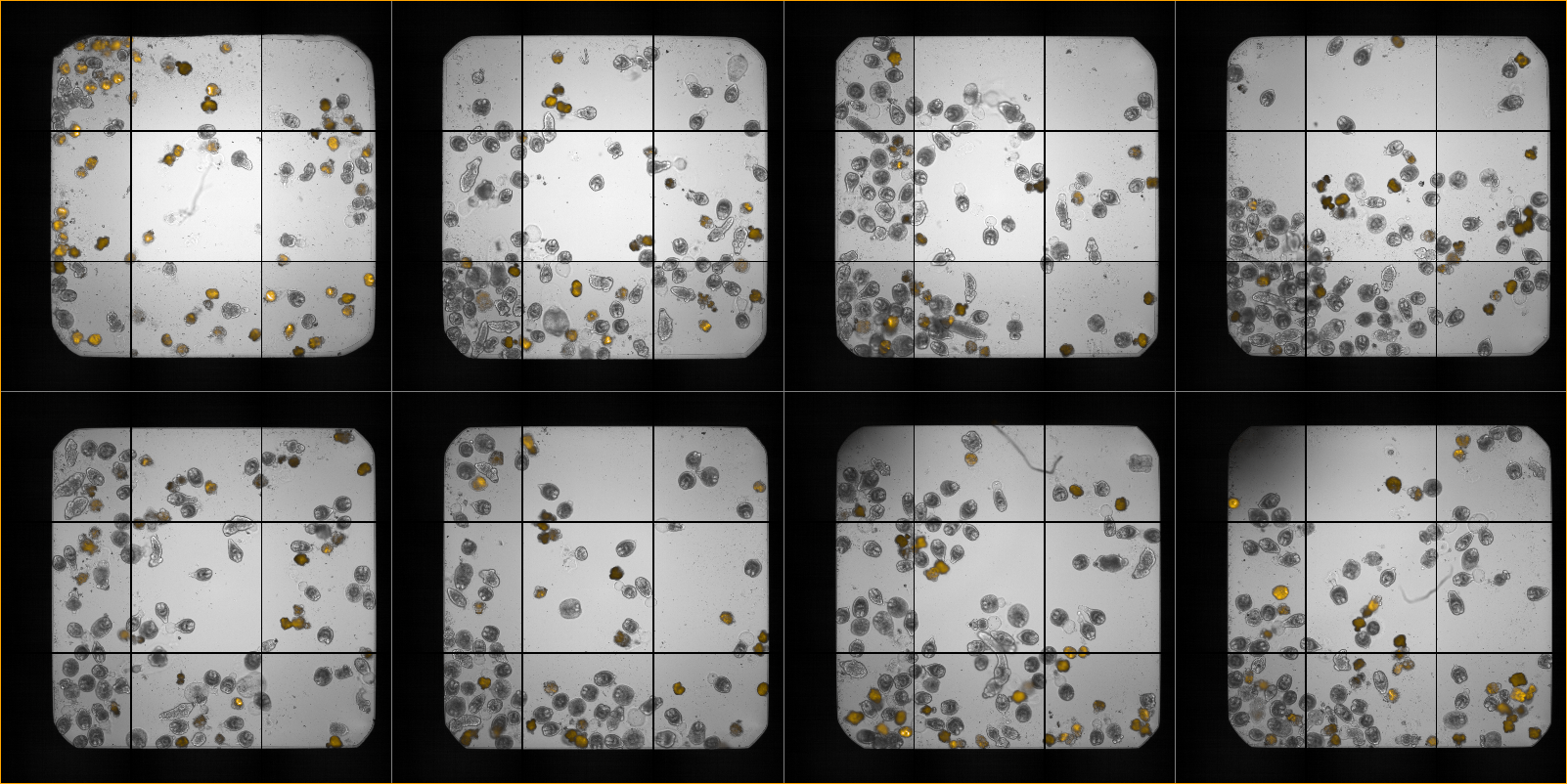

Supplement: Supplementary file 5 — Additional file 5: Dataset S4. PSC images of 16 drug treatment results in a dose–response assay. [file 13071_2024_6456_MOESM5_ESM.zip › Supplementary file 5/Azeliragon.png]

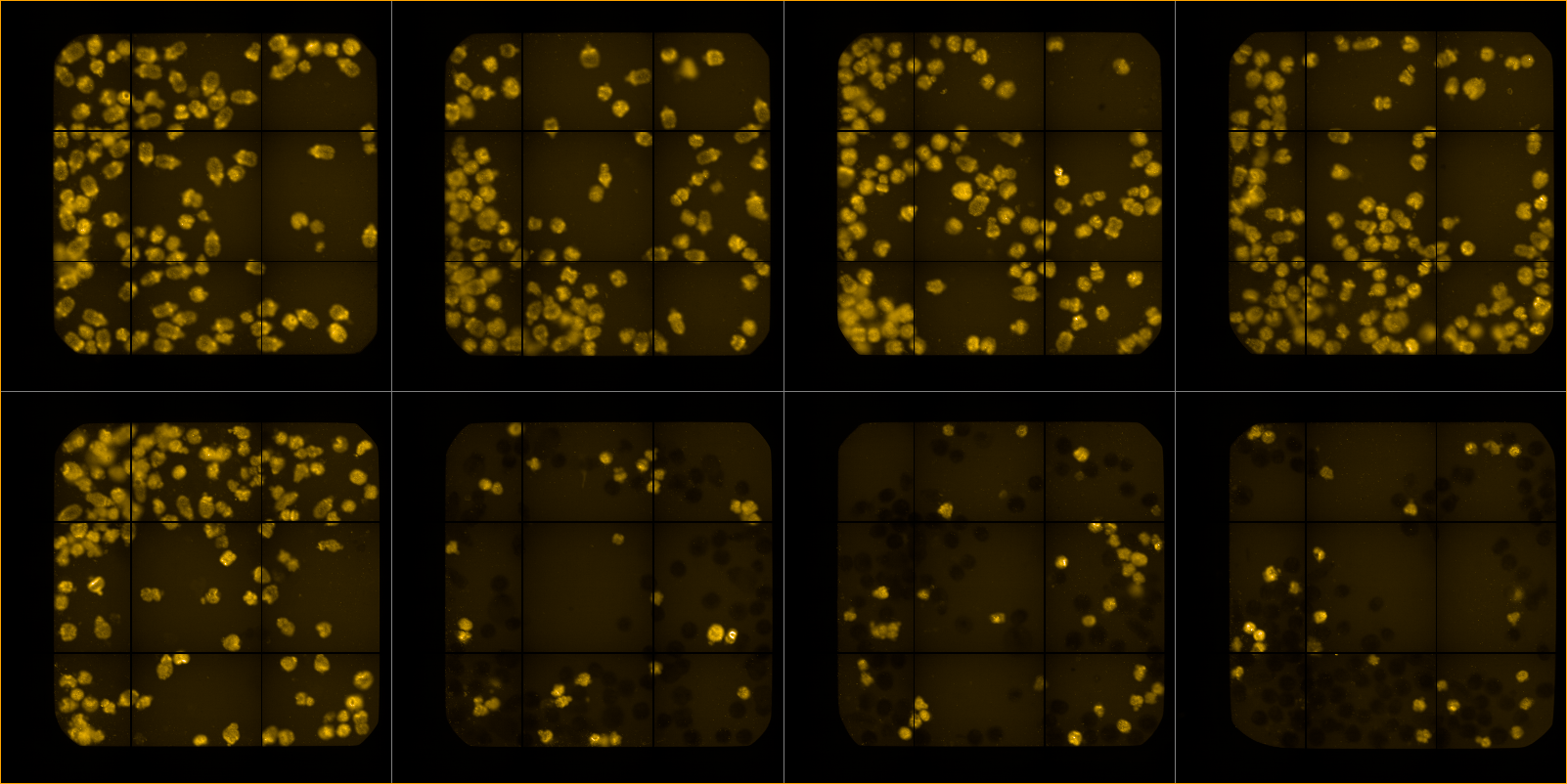

Supplement: Supplementary file 5 — Additional file 5: Dataset S4. PSC images of 16 drug treatment results in a dose–response assay. [file 13071_2024_6456_MOESM5_ESM.zip › Supplementary file 5/Bardoxolone Methyl-2.png]

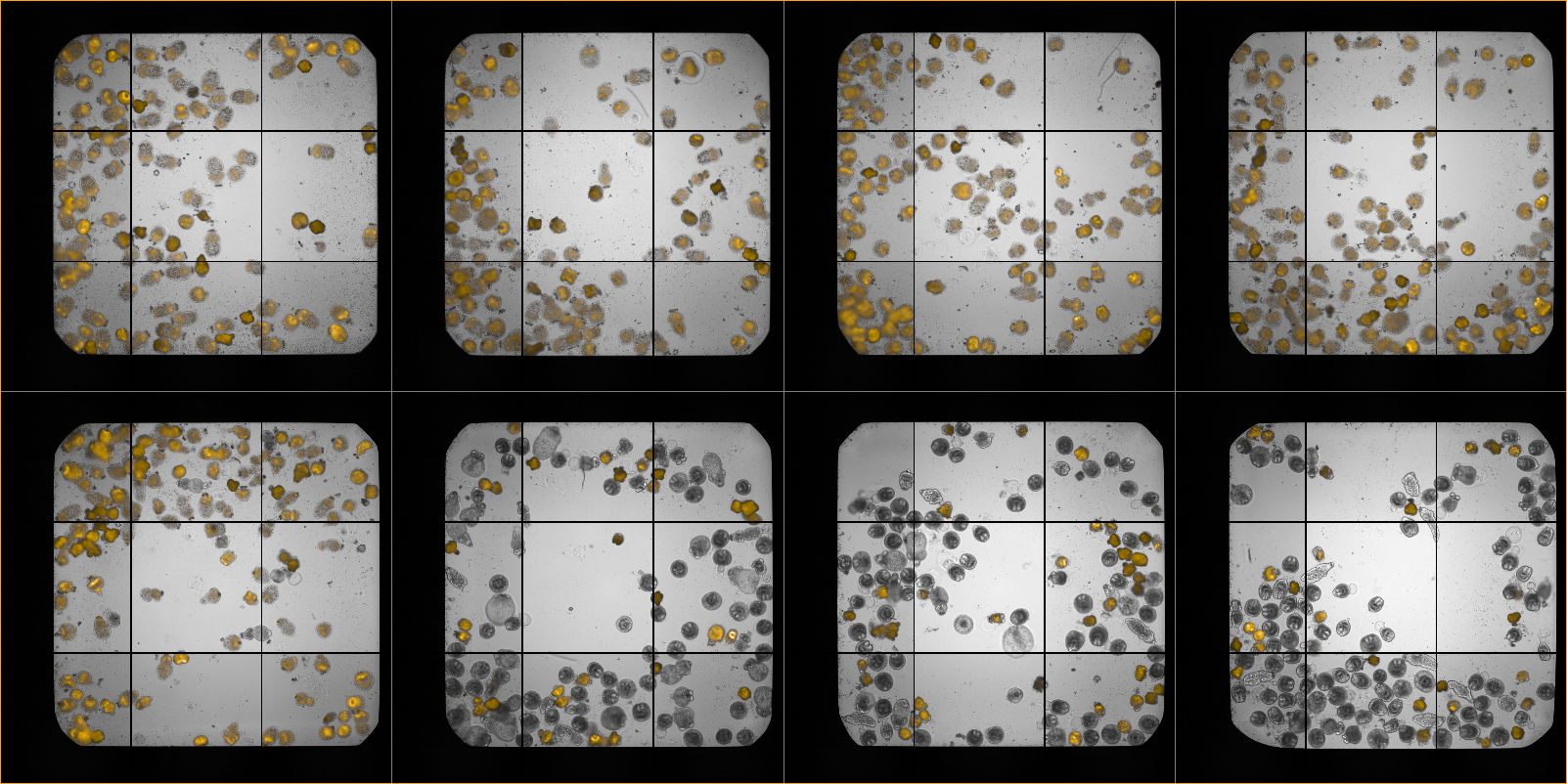

Supplement: Supplementary file 5 — Additional file 5: Dataset S4. PSC images of 16 drug treatment results in a dose–response assay. [file 13071_2024_6456_MOESM5_ESM.zip › Supplementary file 5/Bardoxolone Methyl.png]

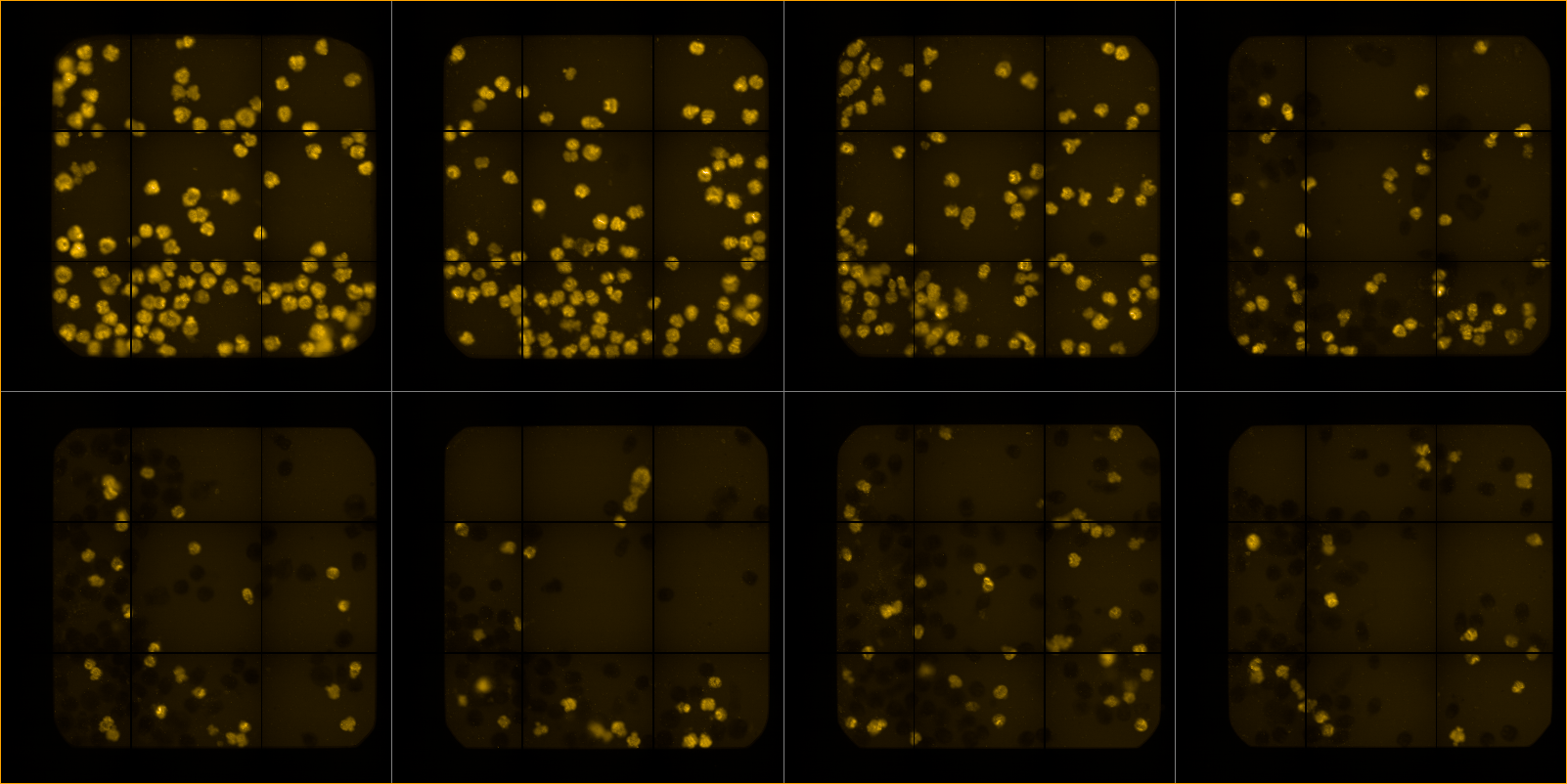

Supplement: Supplementary file 5 — Additional file 5: Dataset S4. PSC images of 16 drug treatment results in a dose–response assay. [file 13071_2024_6456_MOESM5_ESM.zip › Supplementary file 5/Chelerythrine-2.png]

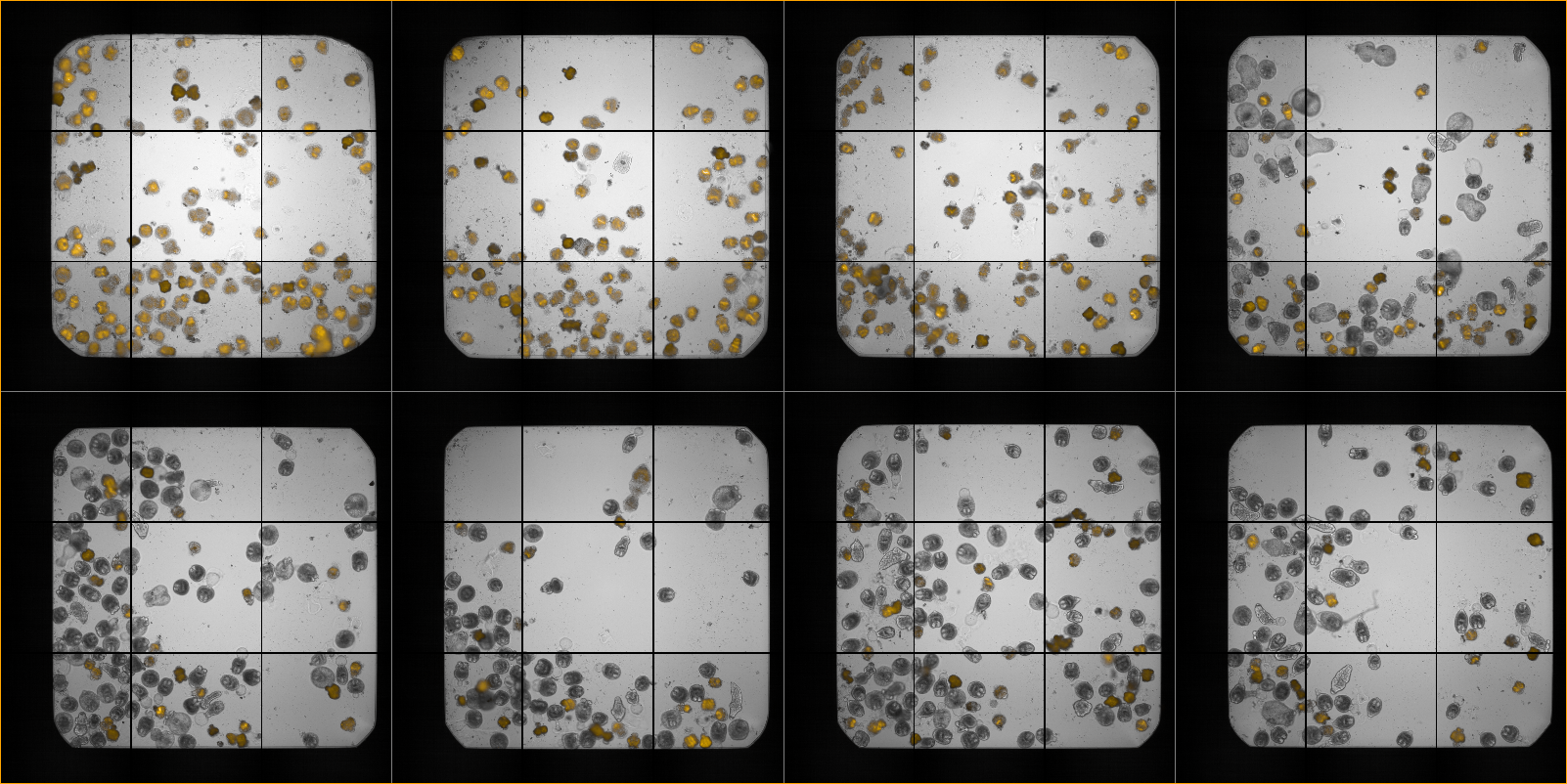

Supplement: Supplementary file 5 — Additional file 5: Dataset S4. PSC images of 16 drug treatment results in a dose–response assay. [file 13071_2024_6456_MOESM5_ESM.zip › Supplementary file 5/Chelerythrine.png]

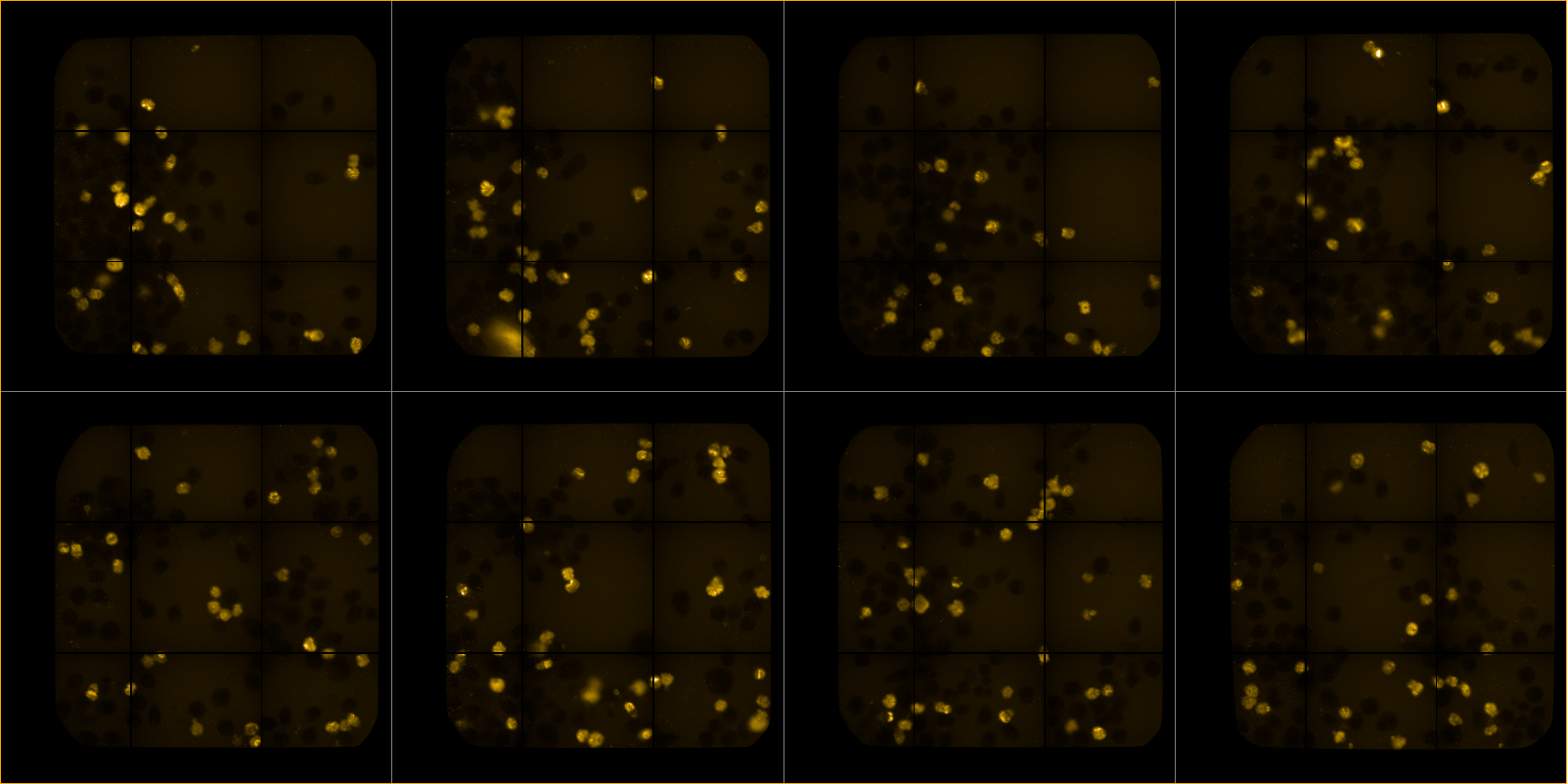

Supplement: Supplementary file 5 — Additional file 5: Dataset S4. PSC images of 16 drug treatment results in a dose–response assay. [file 13071_2024_6456_MOESM5_ESM.zip › Supplementary file 5/DMSO-2.png]

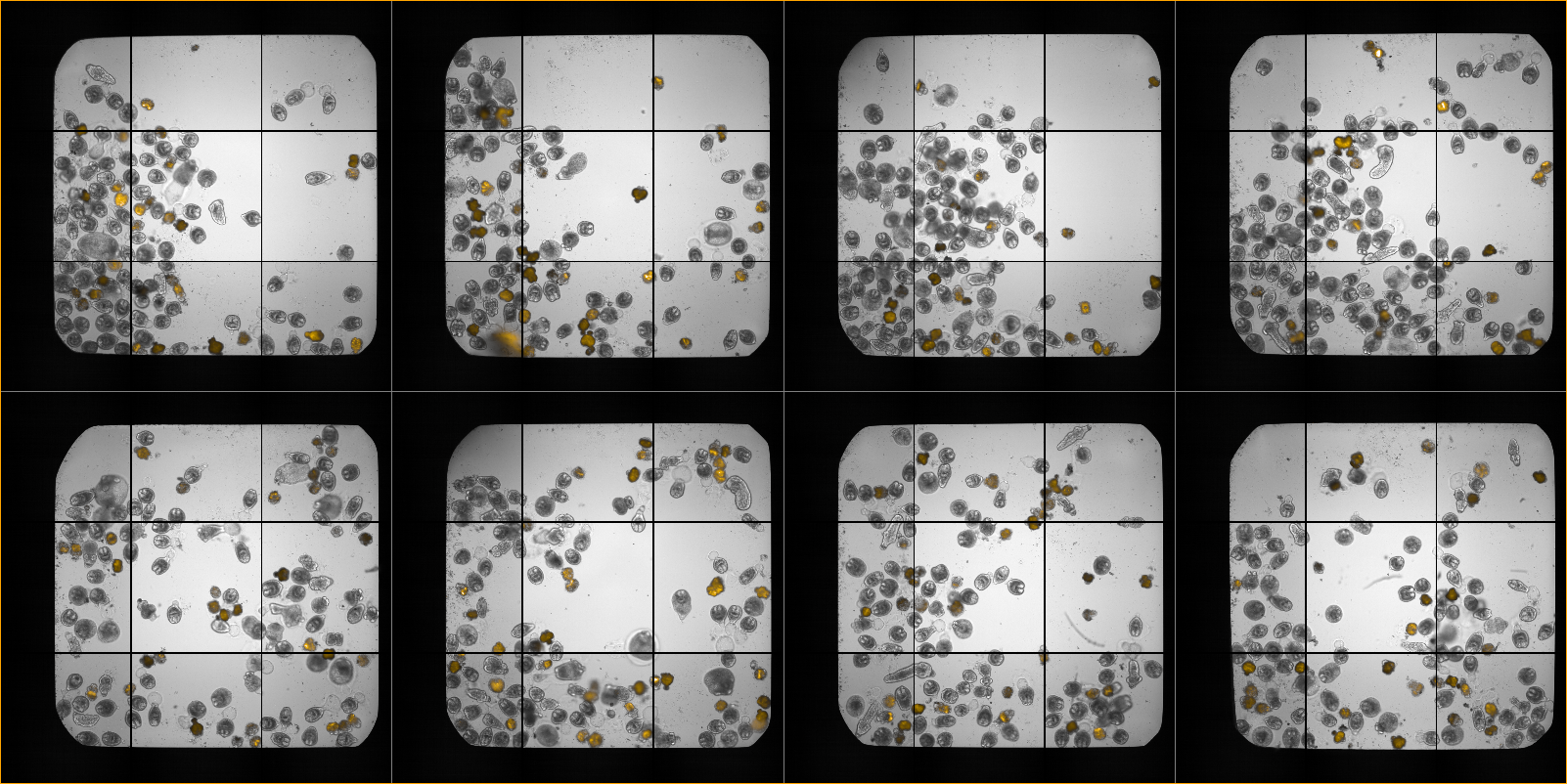

Supplement: Supplementary file 5 — Additional file 5: Dataset S4. PSC images of 16 drug treatment results in a dose–response assay. [file 13071_2024_6456_MOESM5_ESM.zip › Supplementary file 5/DMSO.png]

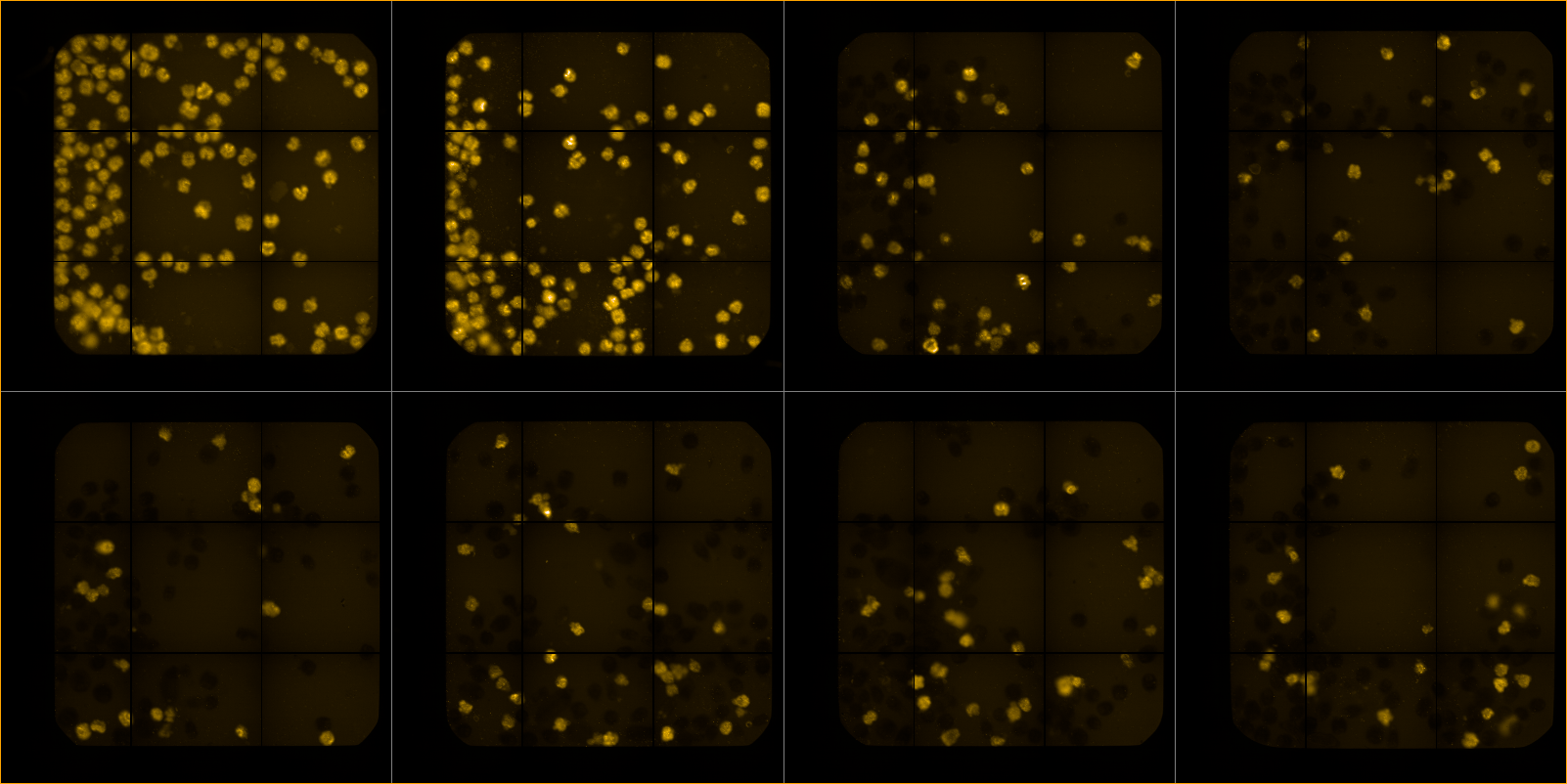

Supplement: Supplementary file 5 — Additional file 5: Dataset S4. PSC images of 16 drug treatment results in a dose–response assay. [file 13071_2024_6456_MOESM5_ESM.zip › Supplementary file 5/Dronedarone HCl-2.png]

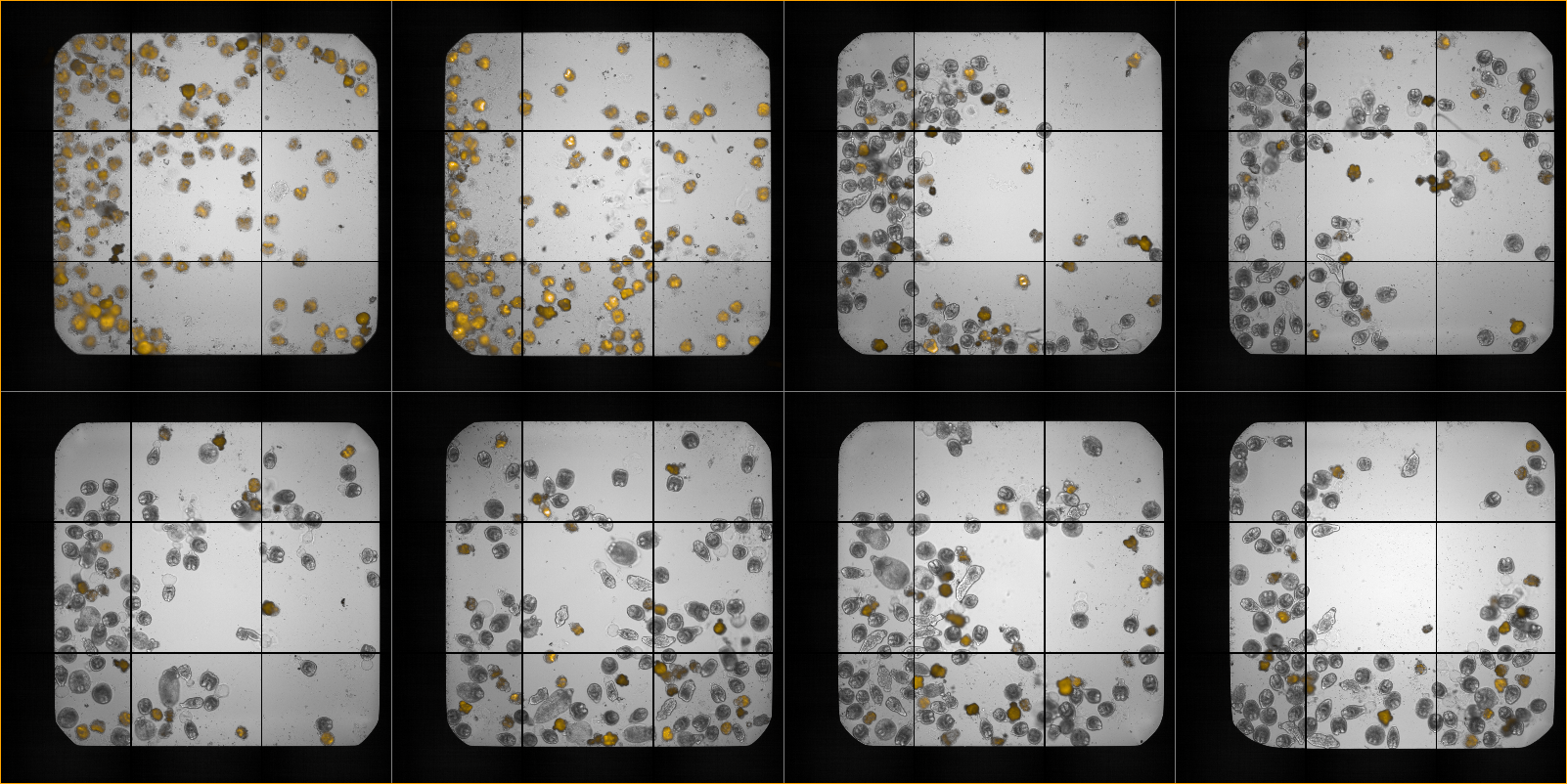

Supplement: Supplementary file 5 — Additional file 5: Dataset S4. PSC images of 16 drug treatment results in a dose–response assay. [file 13071_2024_6456_MOESM5_ESM.zip › Supplementary file 5/Dronedarone HCl.png]

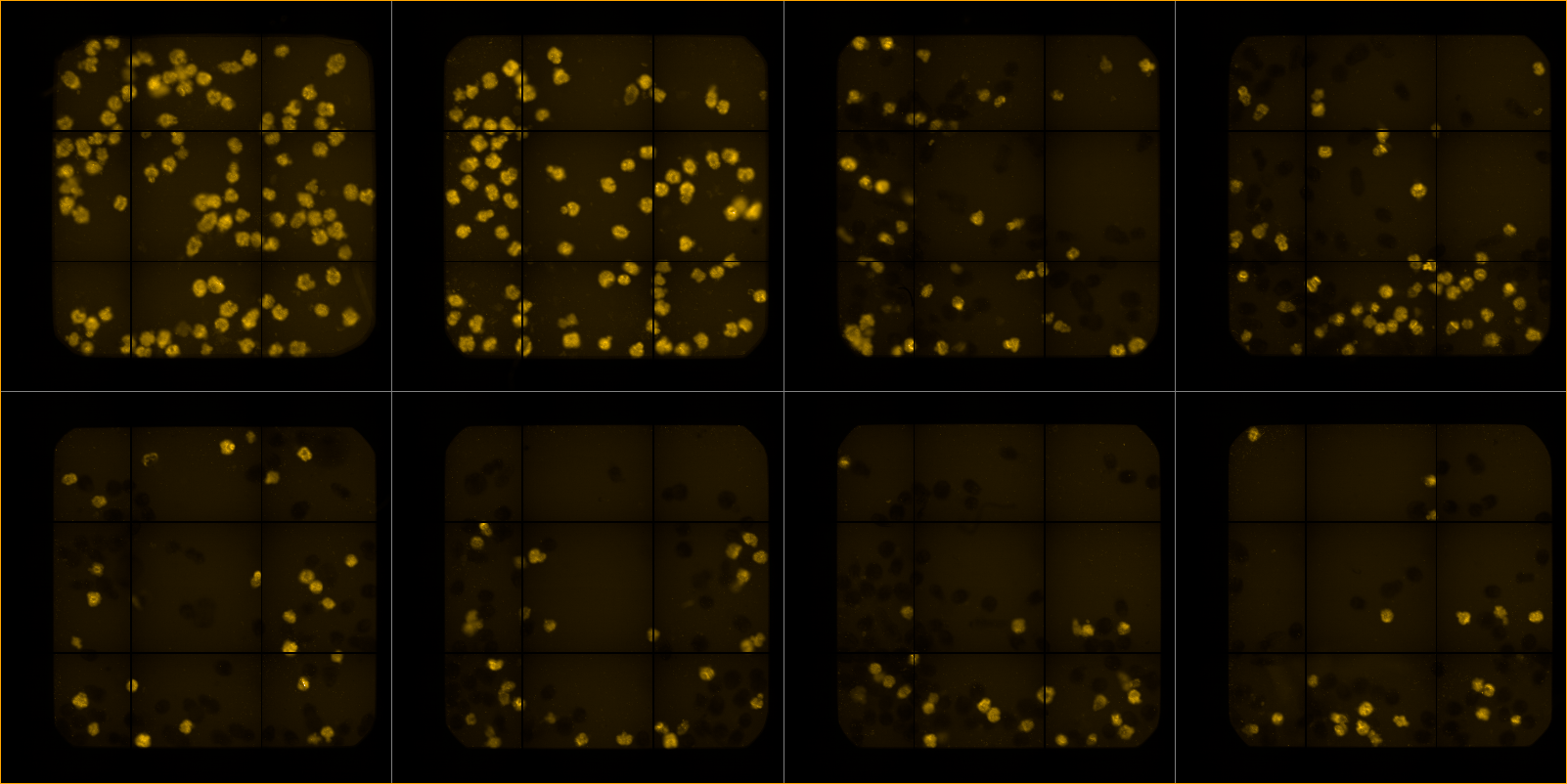

Supplement: Supplementary file 5 — Additional file 5: Dataset S4. PSC images of 16 drug treatment results in a dose–response assay. [file 13071_2024_6456_MOESM5_ESM.zip › Supplementary file 5/Ebastine-2.png]

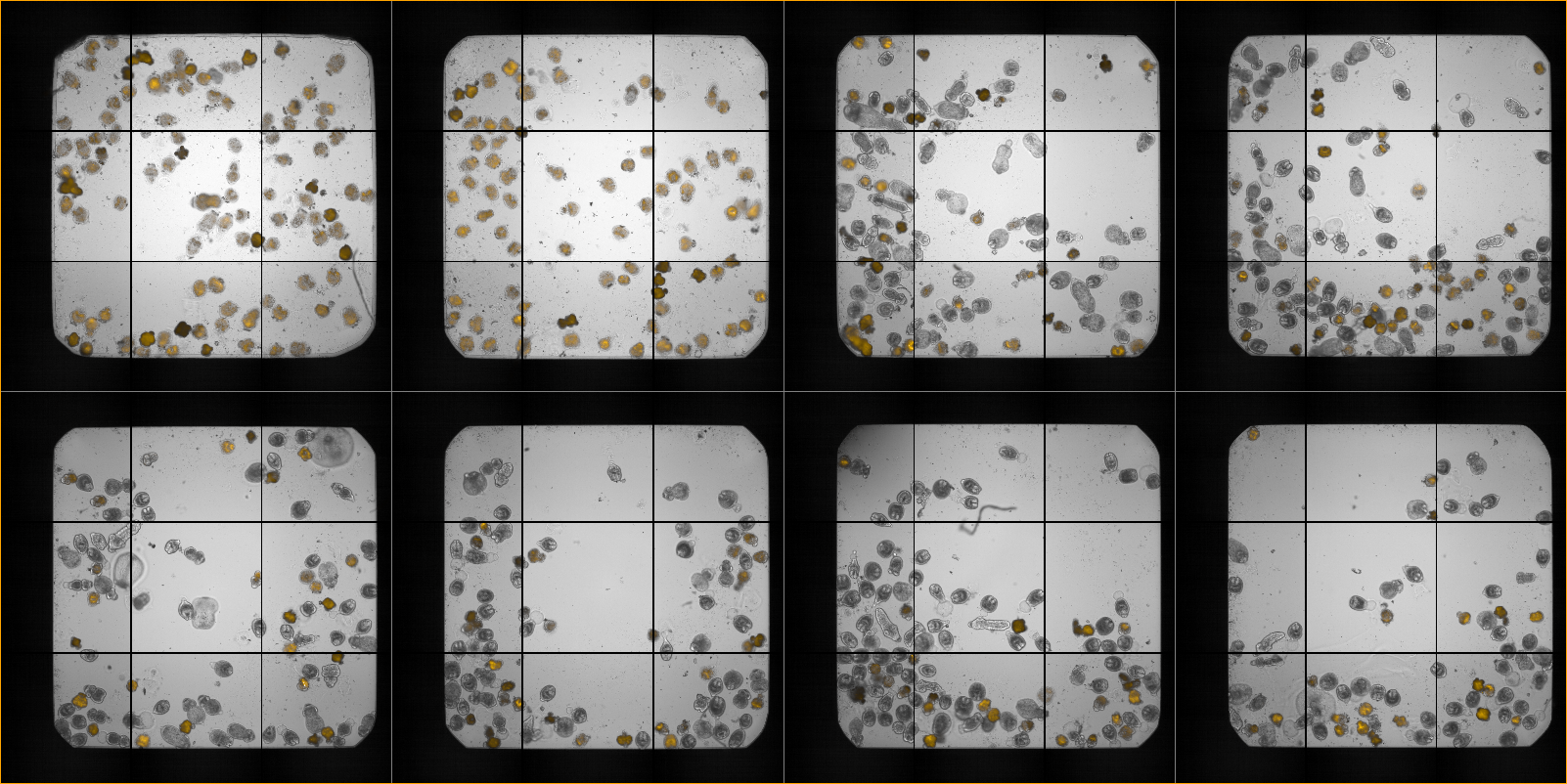

Supplement: Supplementary file 5 — Additional file 5: Dataset S4. PSC images of 16 drug treatment results in a dose–response assay. [file 13071_2024_6456_MOESM5_ESM.zip › Supplementary file 5/Ebastine.png]

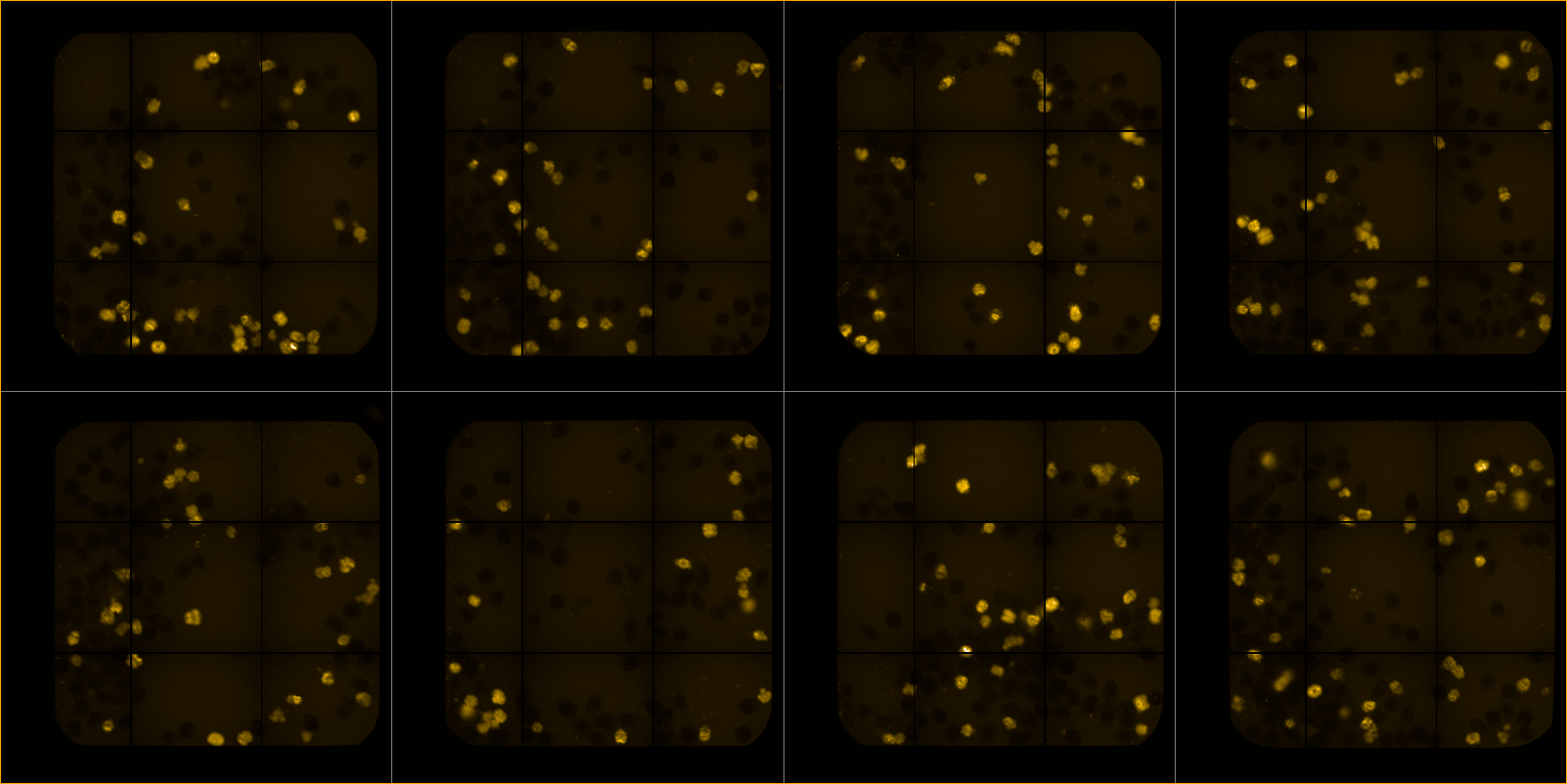

Supplement: Supplementary file 5 — Additional file 5: Dataset S4. PSC images of 16 drug treatment results in a dose–response assay. [file 13071_2024_6456_MOESM5_ESM.zip › Supplementary file 5/Fexofenadine-2.png]

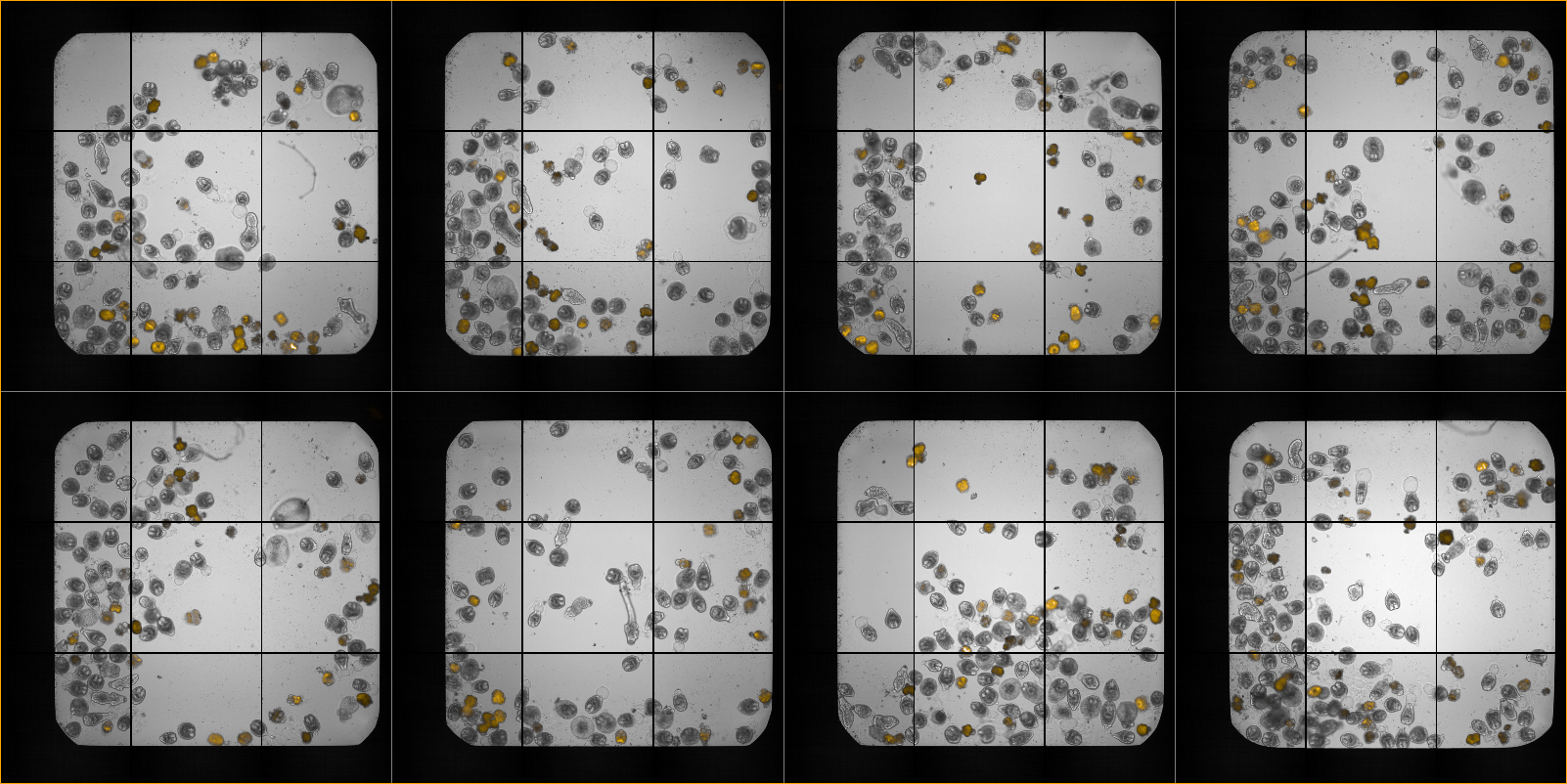

Supplement: Supplementary file 5 — Additional file 5: Dataset S4. PSC images of 16 drug treatment results in a dose–response assay. [file 13071_2024_6456_MOESM5_ESM.zip › Supplementary file 5/Fexofenadine.png]

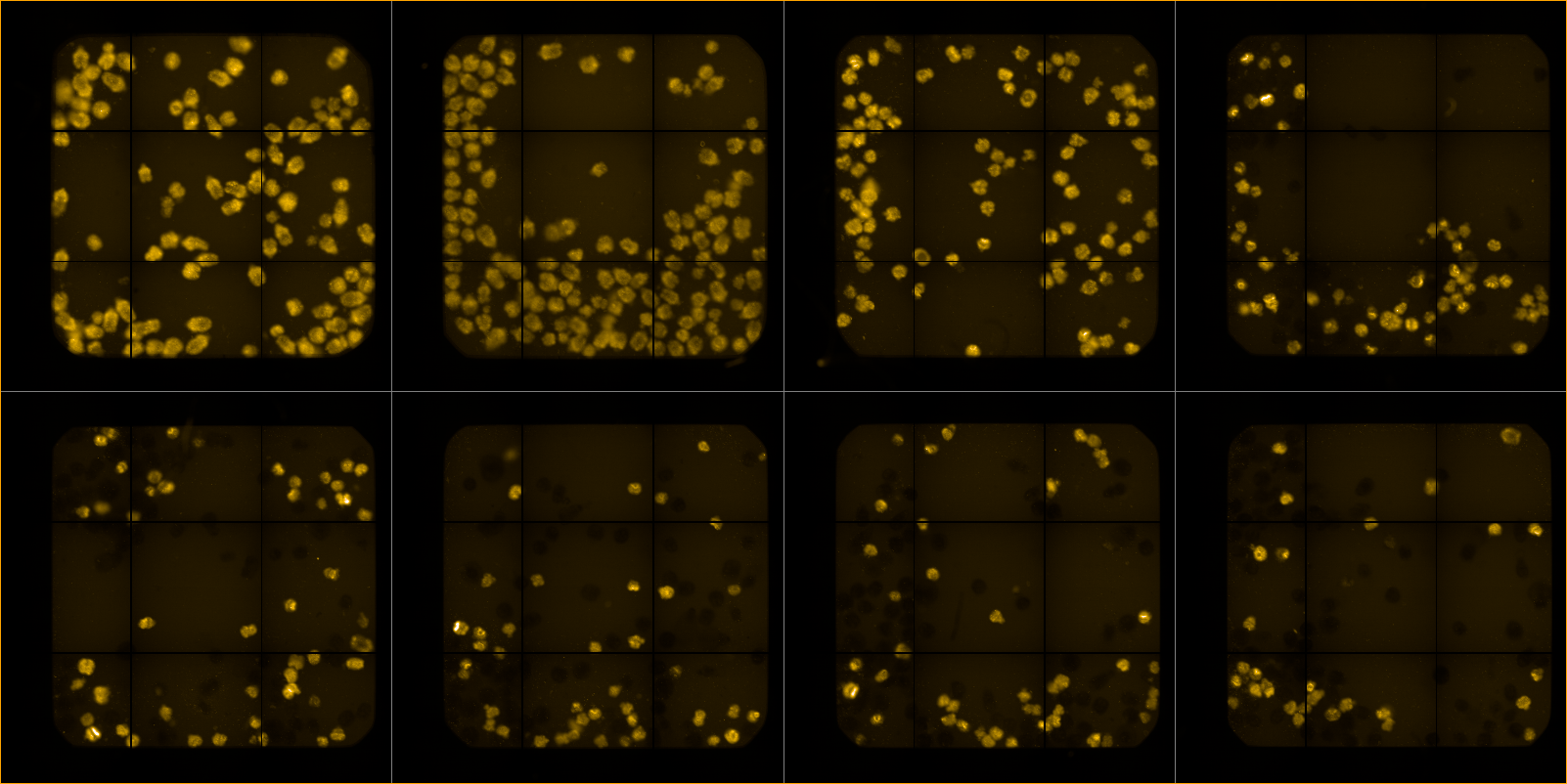

Supplement: Supplementary file 5 — Additional file 5: Dataset S4. PSC images of 16 drug treatment results in a dose–response assay. [file 13071_2024_6456_MOESM5_ESM.zip › Supplementary file 5/JTC-801-2.png]

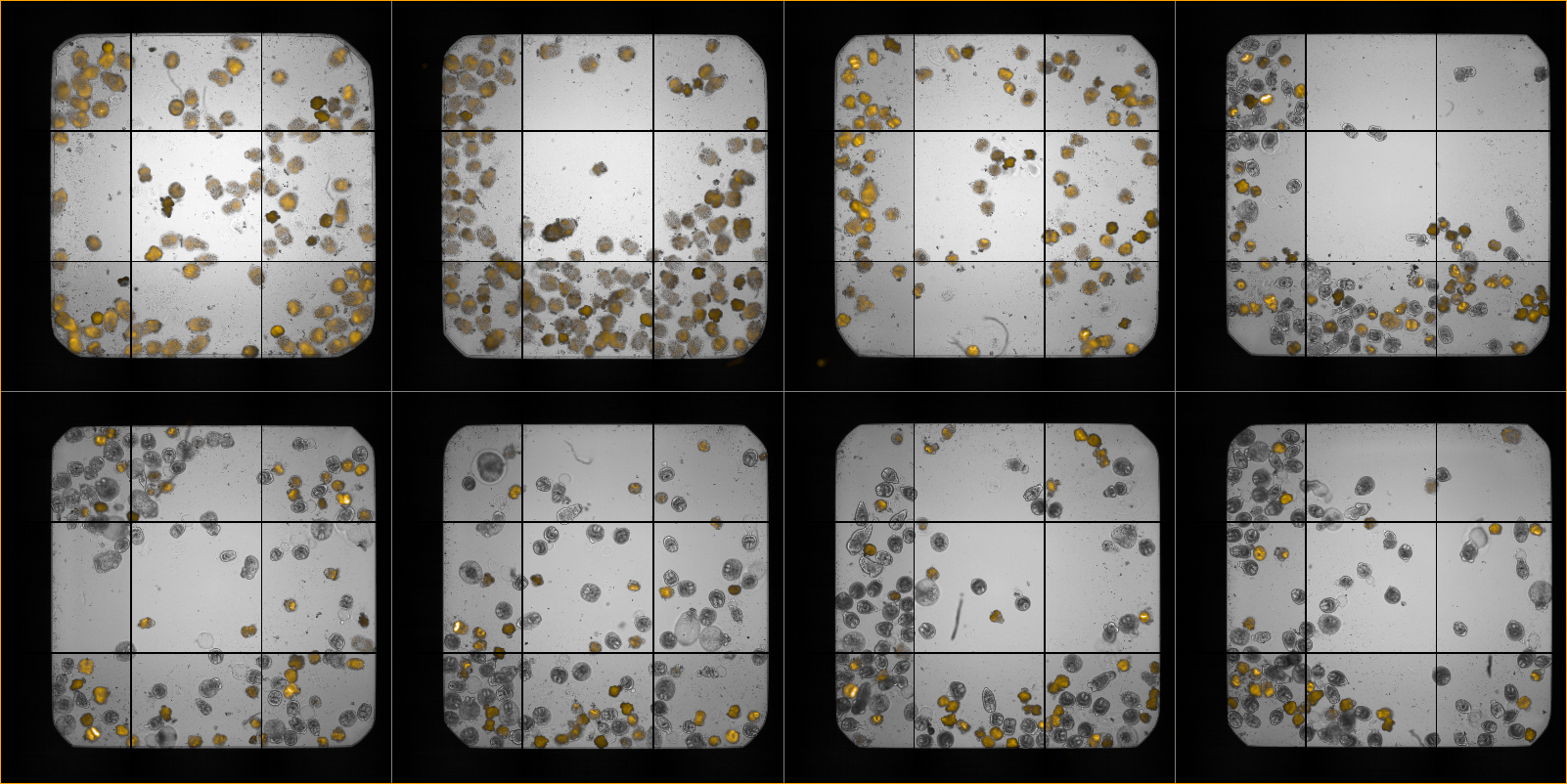

Supplement: Supplementary file 5 — Additional file 5: Dataset S4. PSC images of 16 drug treatment results in a dose–response assay. [file 13071_2024_6456_MOESM5_ESM.zip › Supplementary file 5/JTC-801.png]

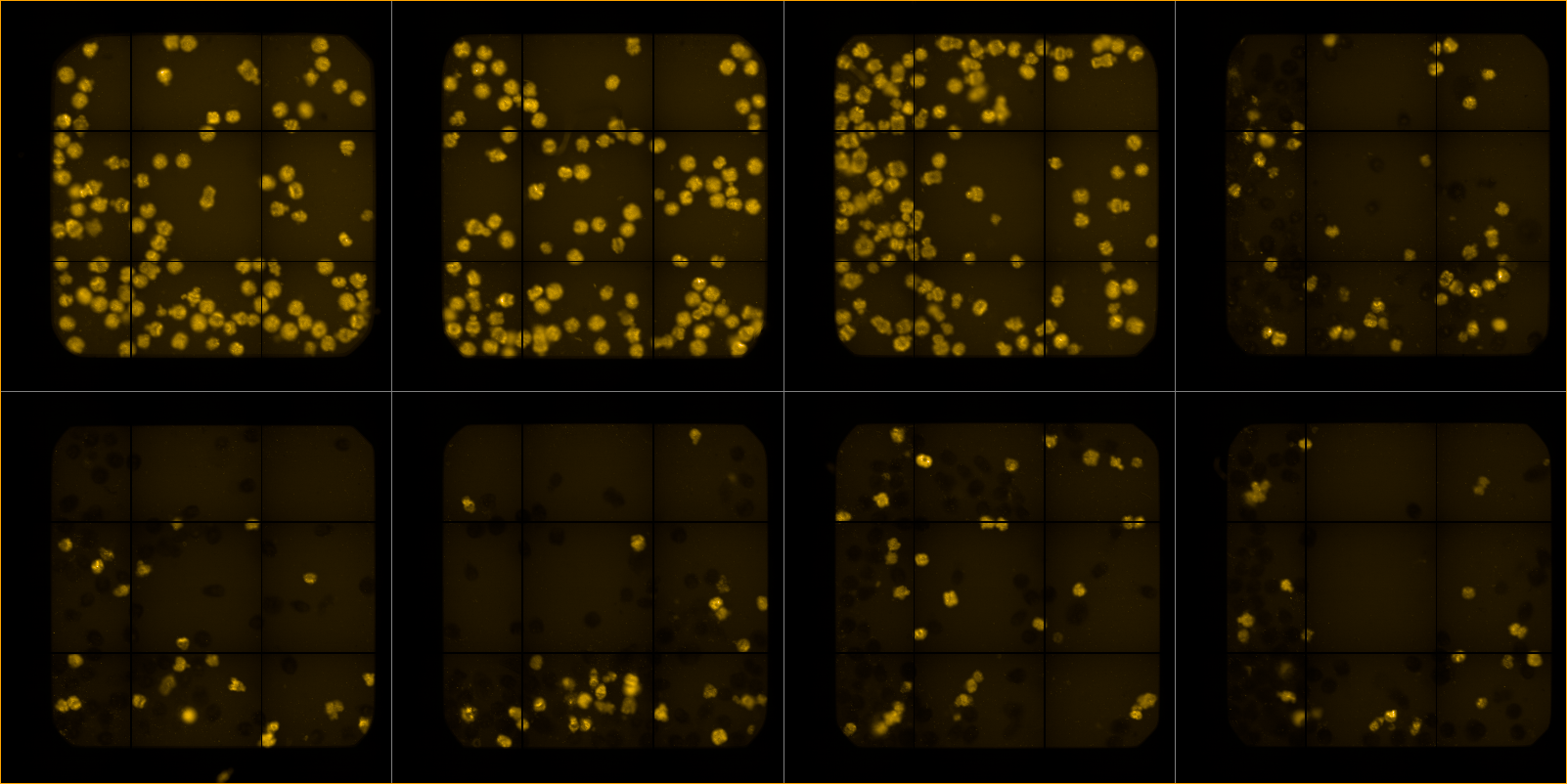

Supplement: Supplementary file 5 — Additional file 5: Dataset S4. PSC images of 16 drug treatment results in a dose–response assay. [file 13071_2024_6456_MOESM5_ESM.zip › Supplementary file 5/K4-2.png]

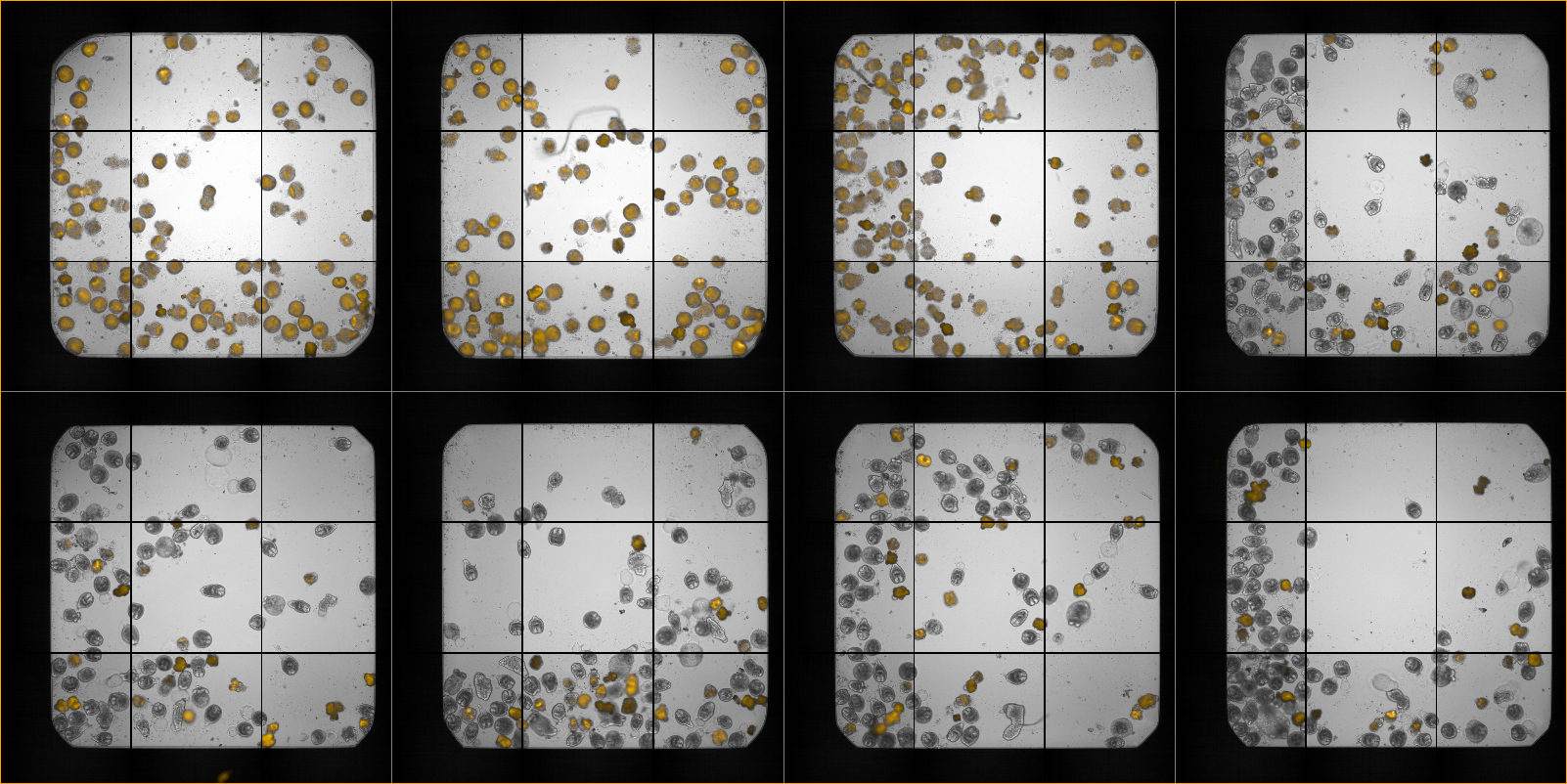

Supplement: Supplementary file 5 — Additional file 5: Dataset S4. PSC images of 16 drug treatment results in a dose–response assay. [file 13071_2024_6456_MOESM5_ESM.zip › Supplementary file 5/K4.png]

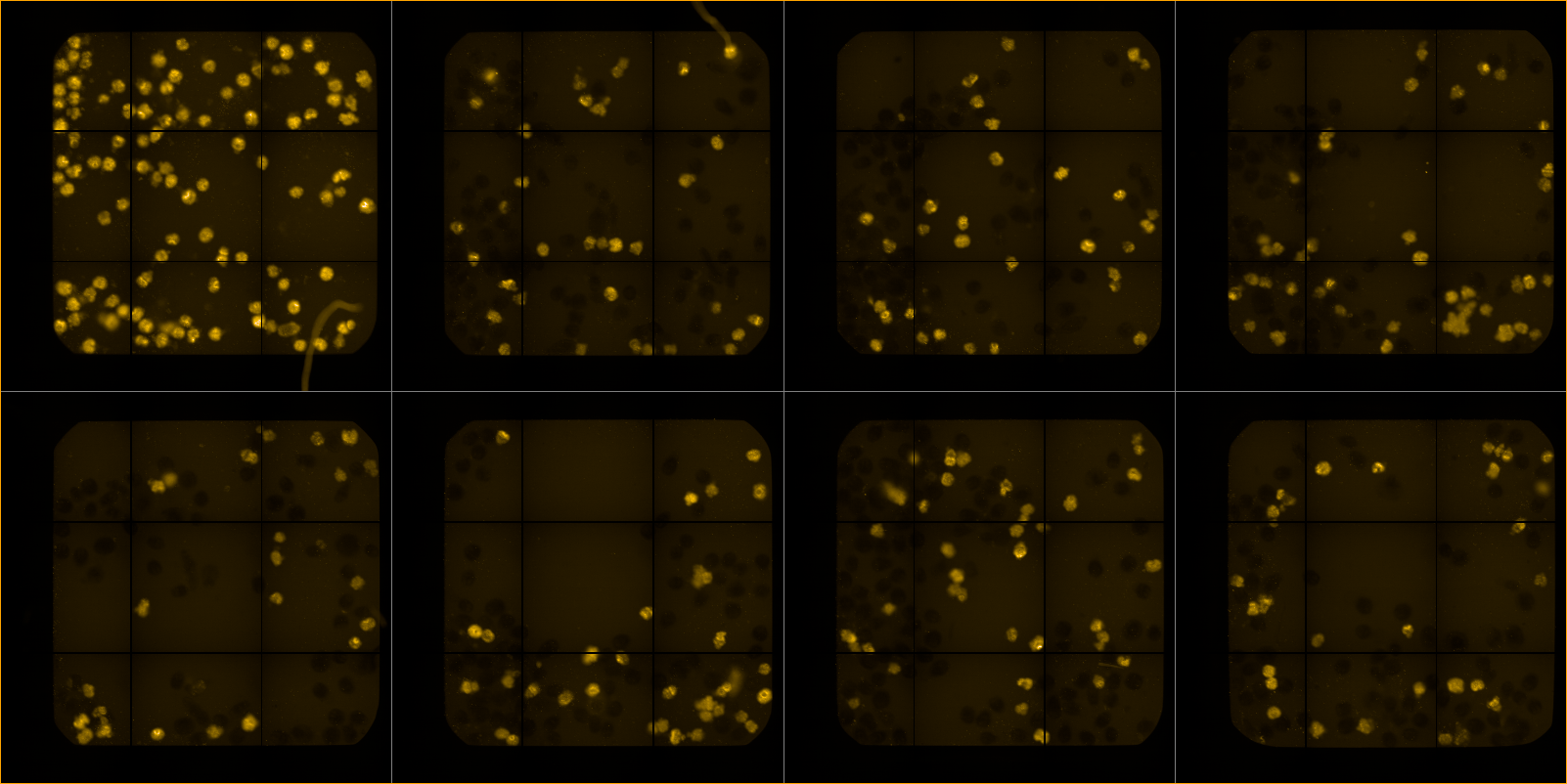

Supplement: Supplementary file 5 — Additional file 5: Dataset S4. PSC images of 16 drug treatment results in a dose–response assay. [file 13071_2024_6456_MOESM5_ESM.zip › Supplementary file 5/Lasofoxifene Tartrate-2.png]

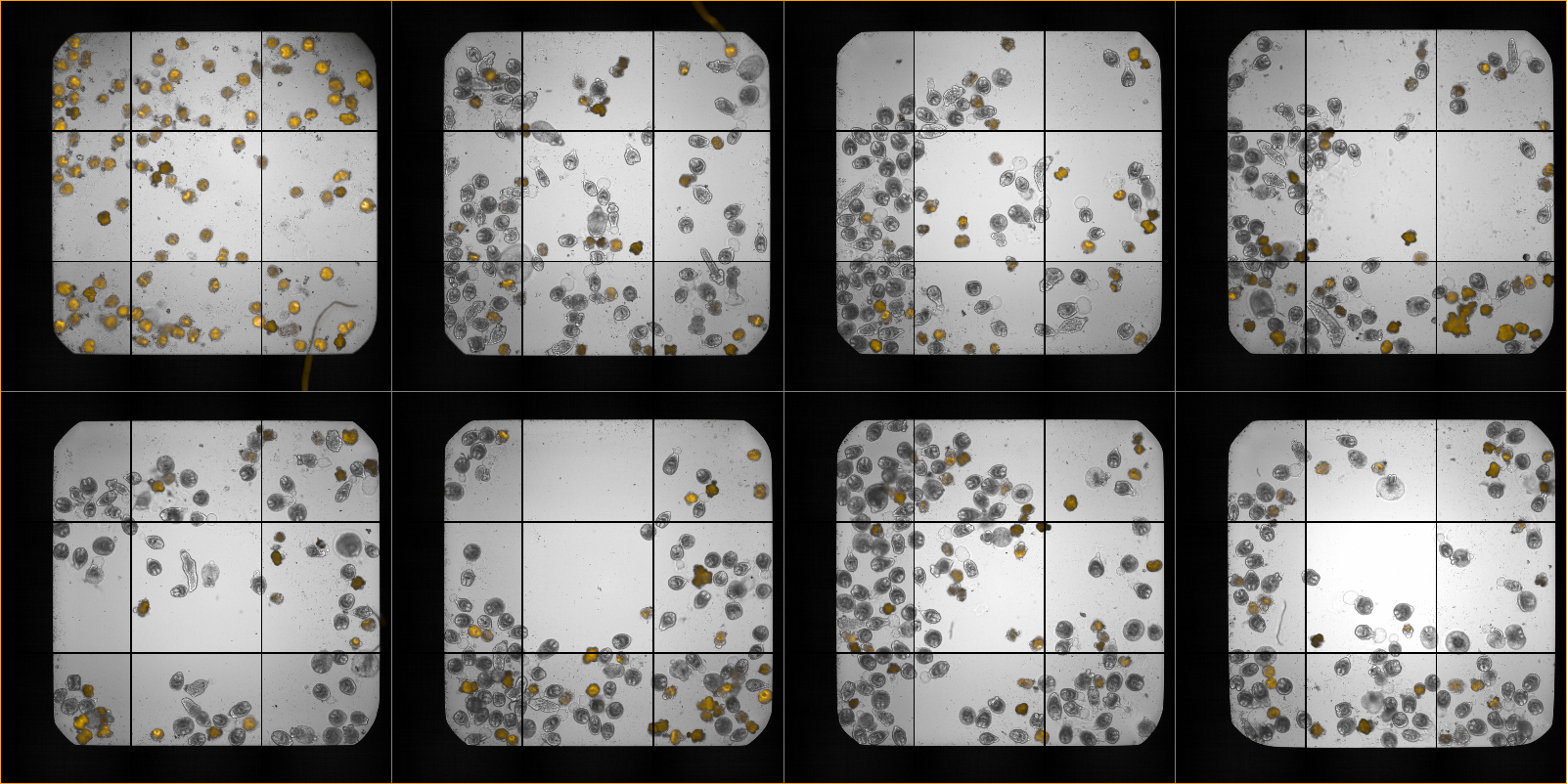

Supplement: Supplementary file 5 — Additional file 5: Dataset S4. PSC images of 16 drug treatment results in a dose–response assay. [file 13071_2024_6456_MOESM5_ESM.zip › Supplementary file 5/Lasofoxifene Tartrate.png]

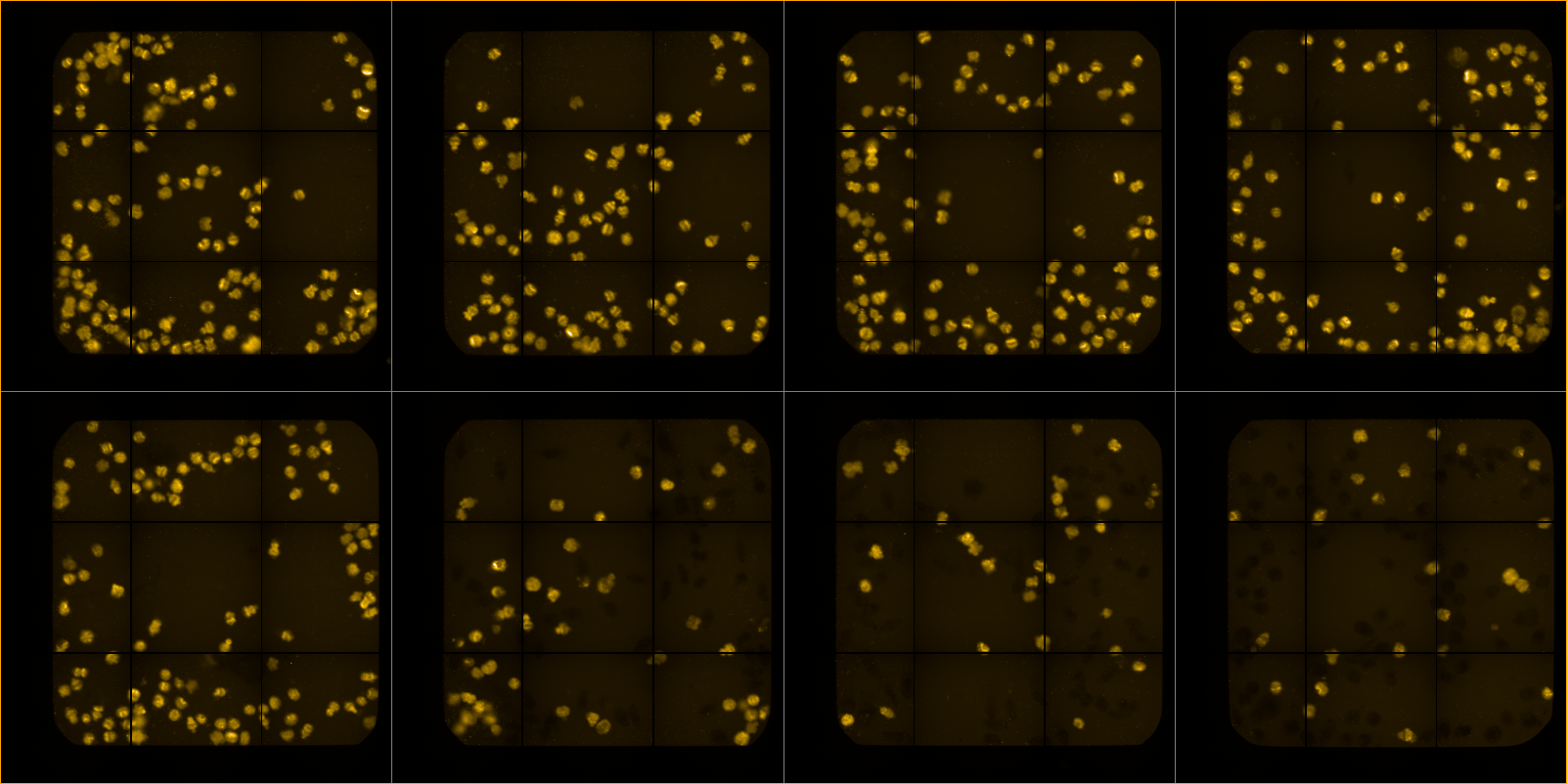

Supplement: Supplementary file 5 — Additional file 5: Dataset S4. PSC images of 16 drug treatment results in a dose–response assay. [file 13071_2024_6456_MOESM5_ESM.zip › Supplementary file 5/Niclosamid-2.png]

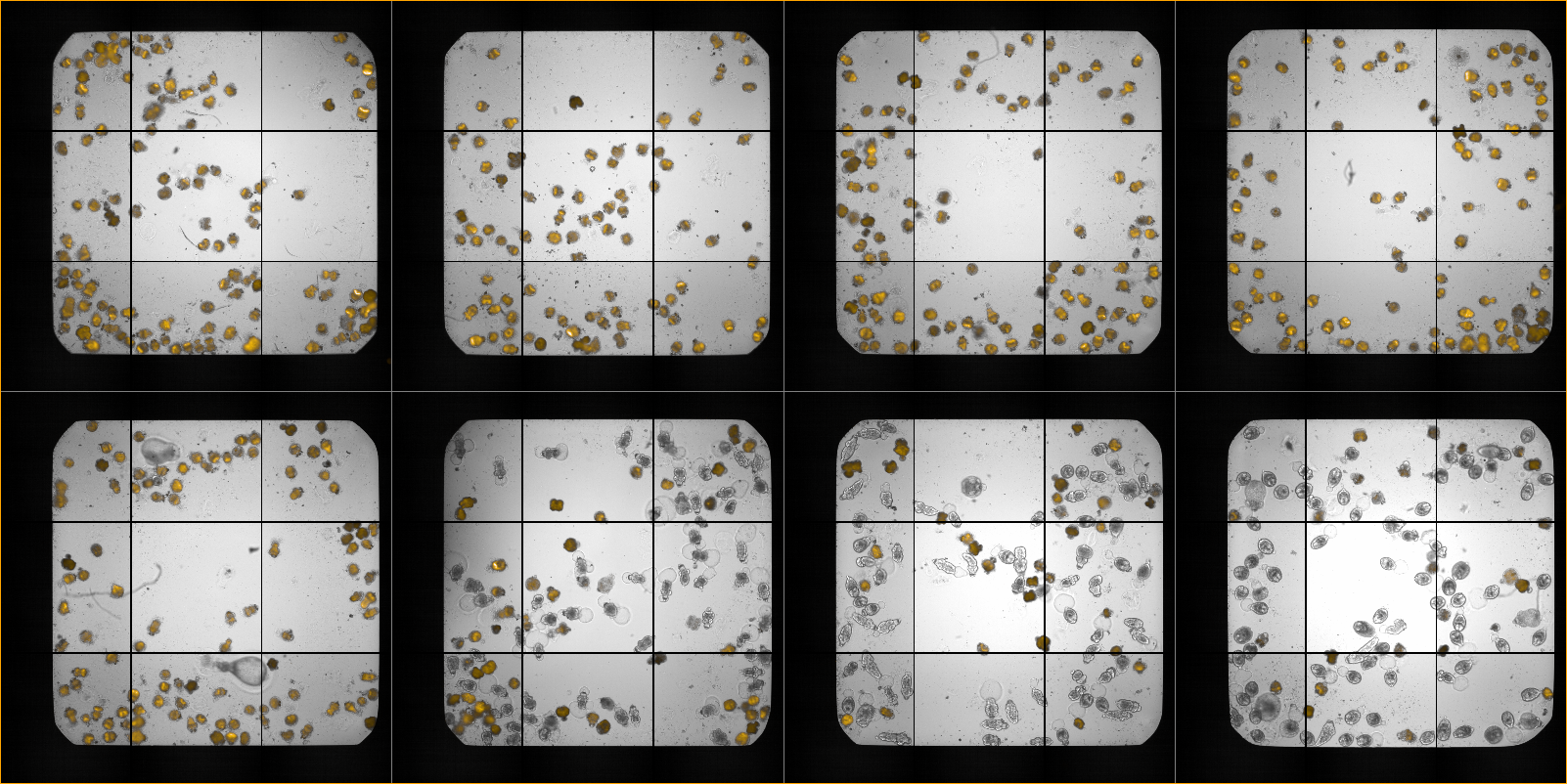

Supplement: Supplementary file 5 — Additional file 5: Dataset S4. PSC images of 16 drug treatment results in a dose–response assay. [file 13071_2024_6456_MOESM5_ESM.zip › Supplementary file 5/Niclosamid.png]

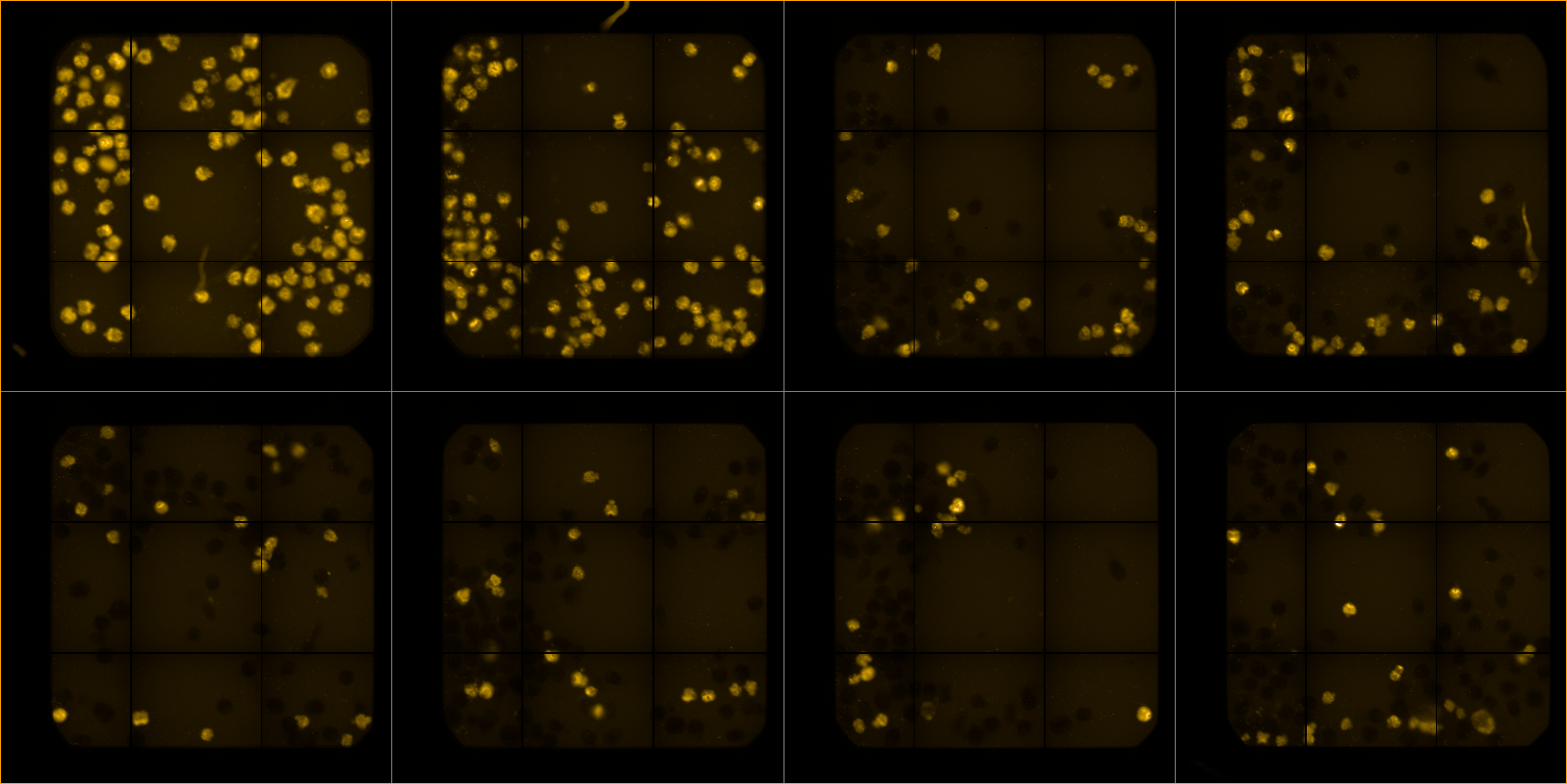

Supplement: Supplementary file 5 — Additional file 5: Dataset S4. PSC images of 16 drug treatment results in a dose–response assay. [file 13071_2024_6456_MOESM5_ESM.zip › Supplementary file 5/NPS-2143-2.png]

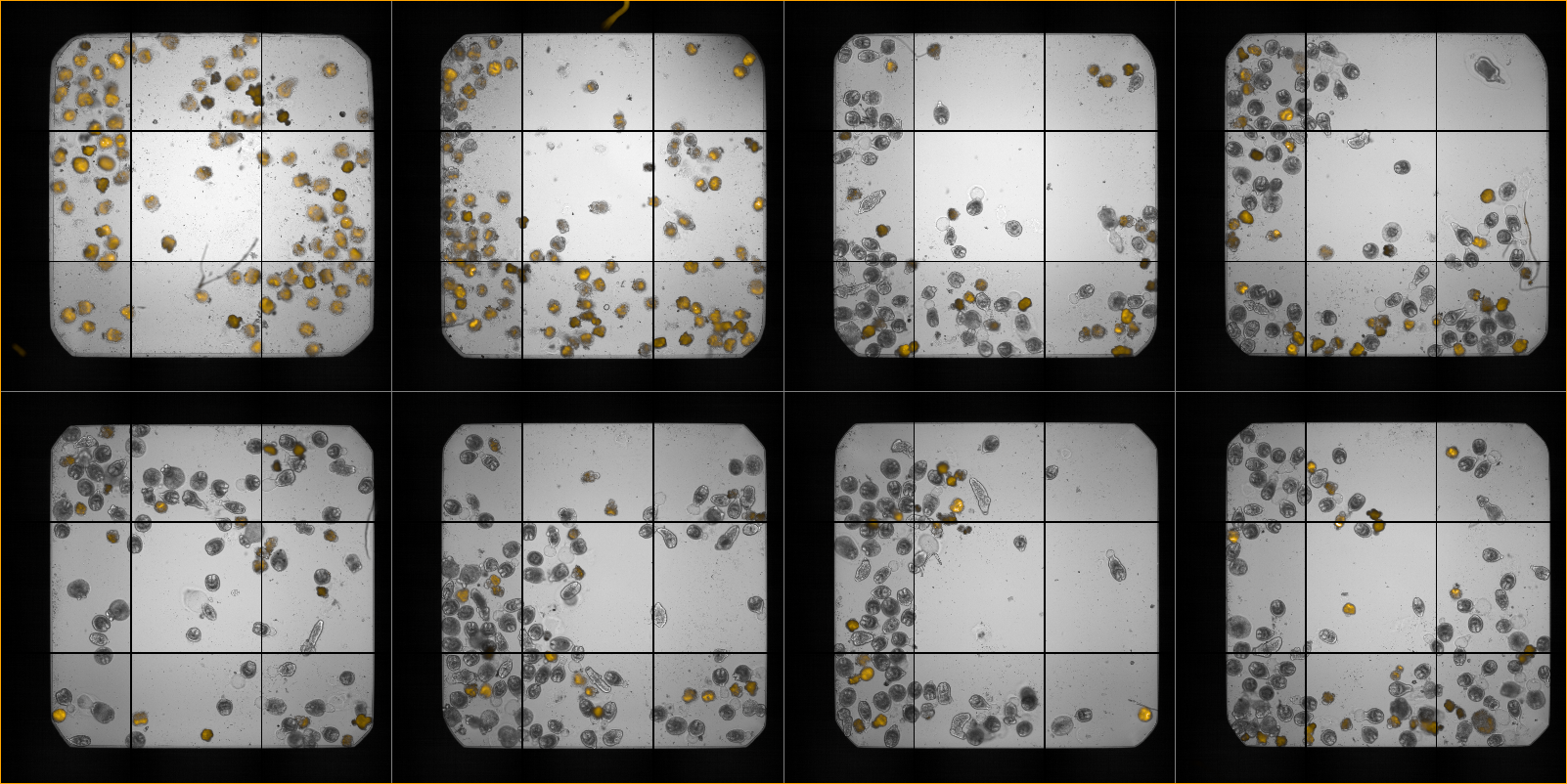

Supplement: Supplementary file 5 — Additional file 5: Dataset S4. PSC images of 16 drug treatment results in a dose–response assay. [file 13071_2024_6456_MOESM5_ESM.zip › Supplementary file 5/NPS-2143.png]

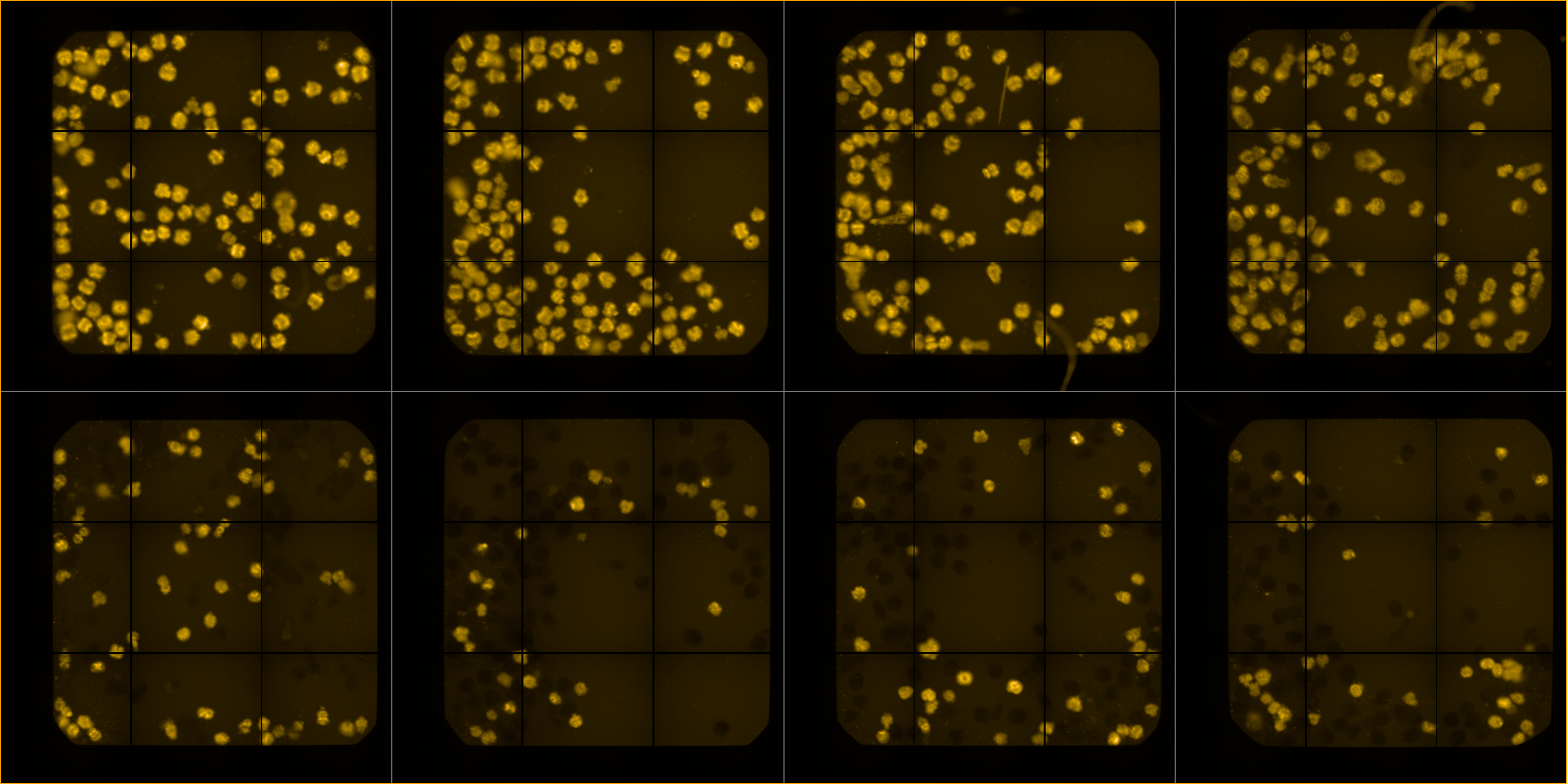

Supplement: Supplementary file 5 — Additional file 5: Dataset S4. PSC images of 16 drug treatment results in a dose–response assay. [file 13071_2024_6456_MOESM5_ESM.zip › Supplementary file 5/Omaveloxolone-2.png]

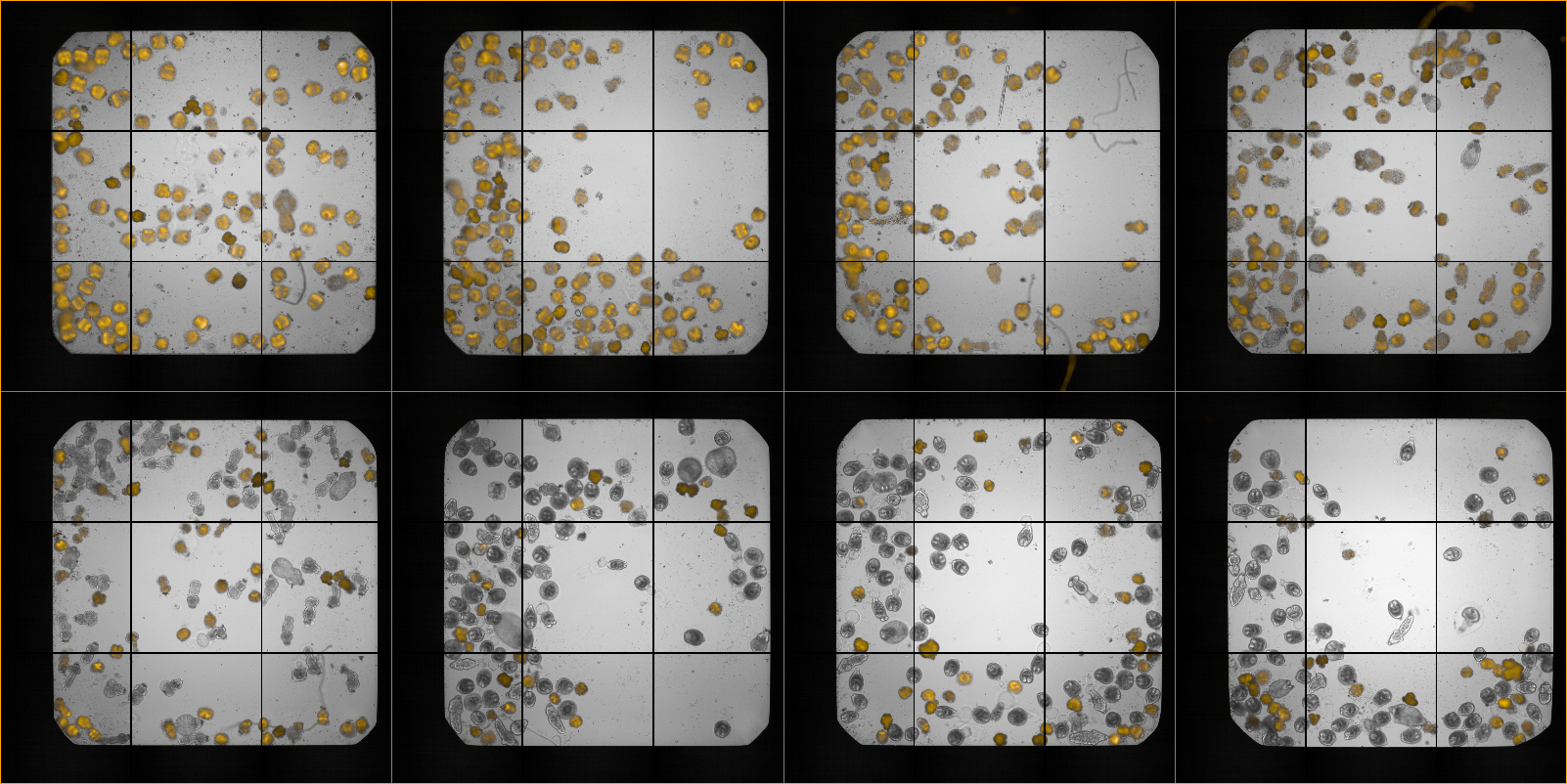

Supplement: Supplementary file 5 — Additional file 5: Dataset S4. PSC images of 16 drug treatment results in a dose–response assay. [file 13071_2024_6456_MOESM5_ESM.zip › Supplementary file 5/Omaveloxolone.png]

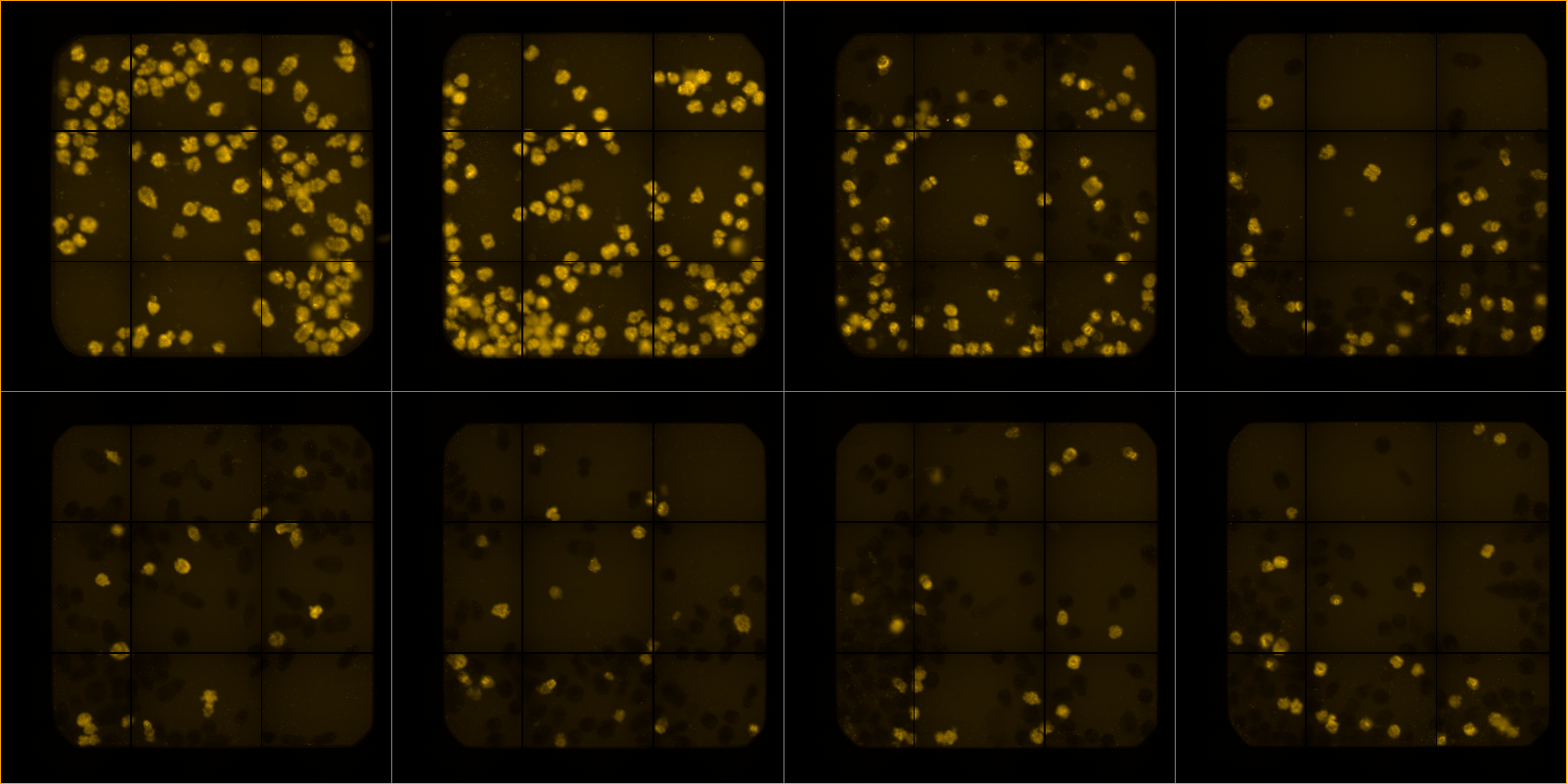

Supplement: Supplementary file 5 — Additional file 5: Dataset S4. PSC images of 16 drug treatment results in a dose–response assay. [file 13071_2024_6456_MOESM5_ESM.zip › Supplementary file 5/Penfluridol-2.png]

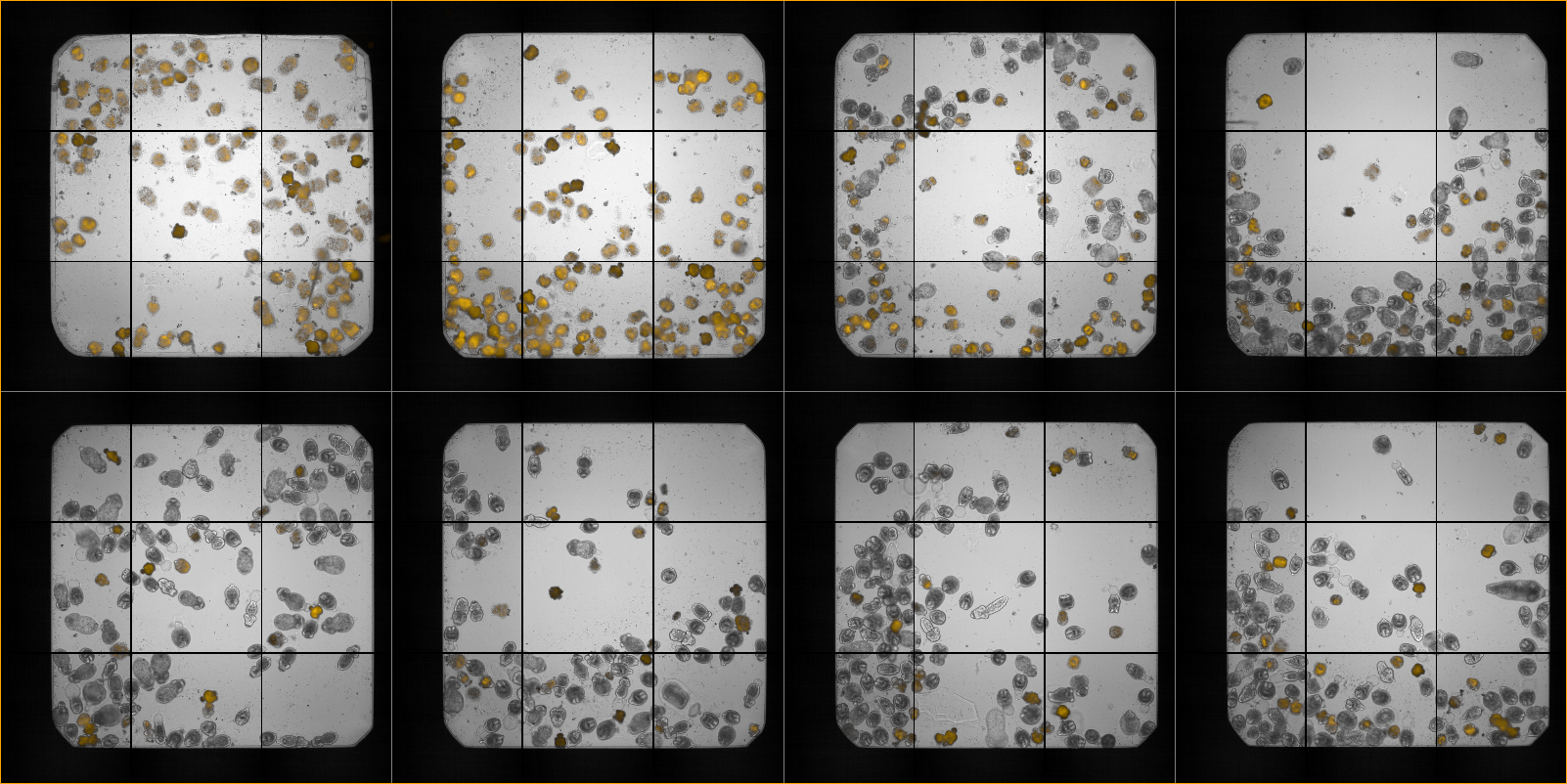

Supplement: Supplementary file 5 — Additional file 5: Dataset S4. PSC images of 16 drug treatment results in a dose–response assay. [file 13071_2024_6456_MOESM5_ESM.zip › Supplementary file 5/Penfluridol.png]

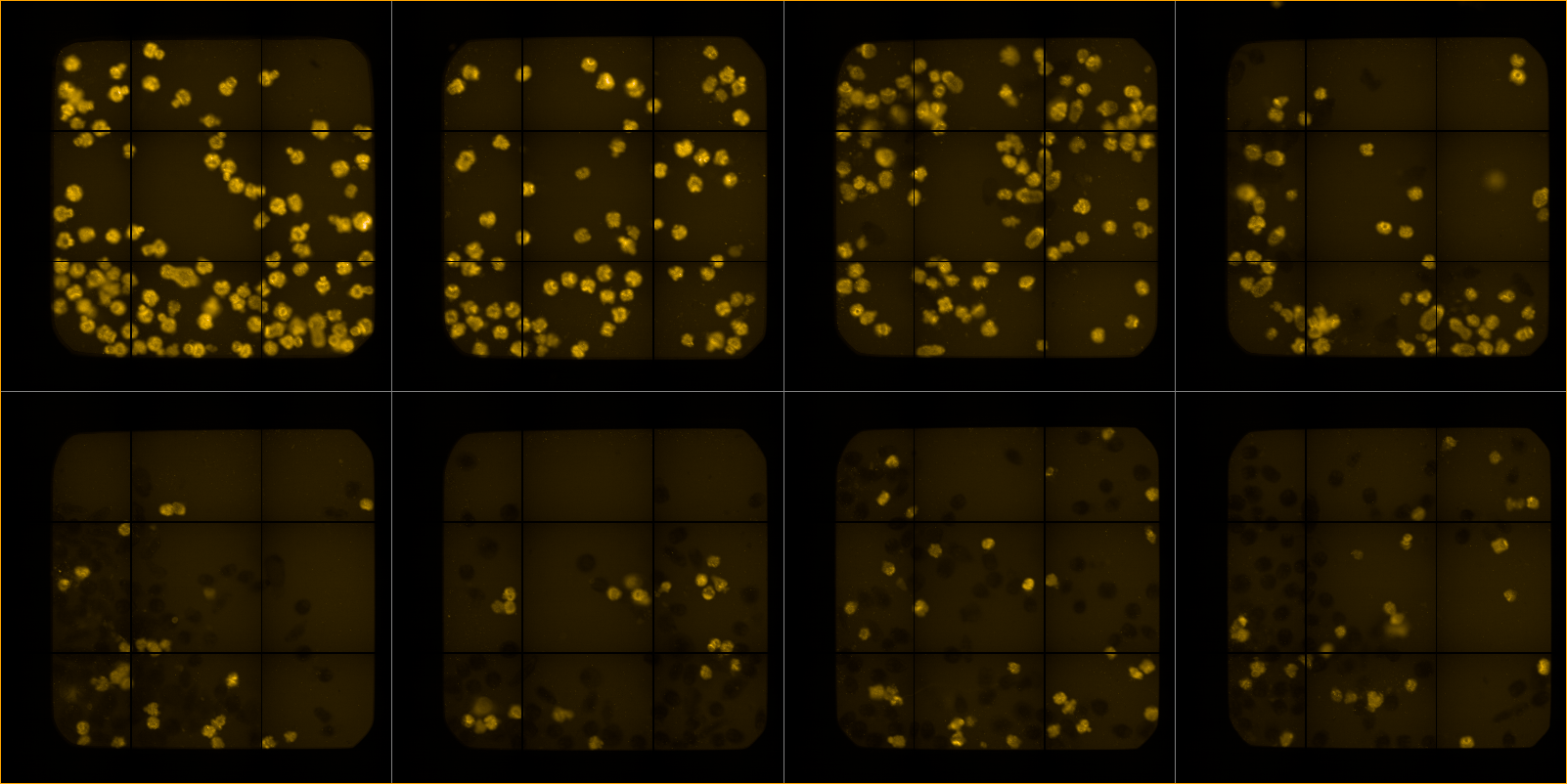

Supplement: Supplementary file 5 — Additional file 5: Dataset S4. PSC images of 16 drug treatment results in a dose–response assay. [file 13071_2024_6456_MOESM5_ESM.zip › Supplementary file 5/Positiv WP1130-2.png]

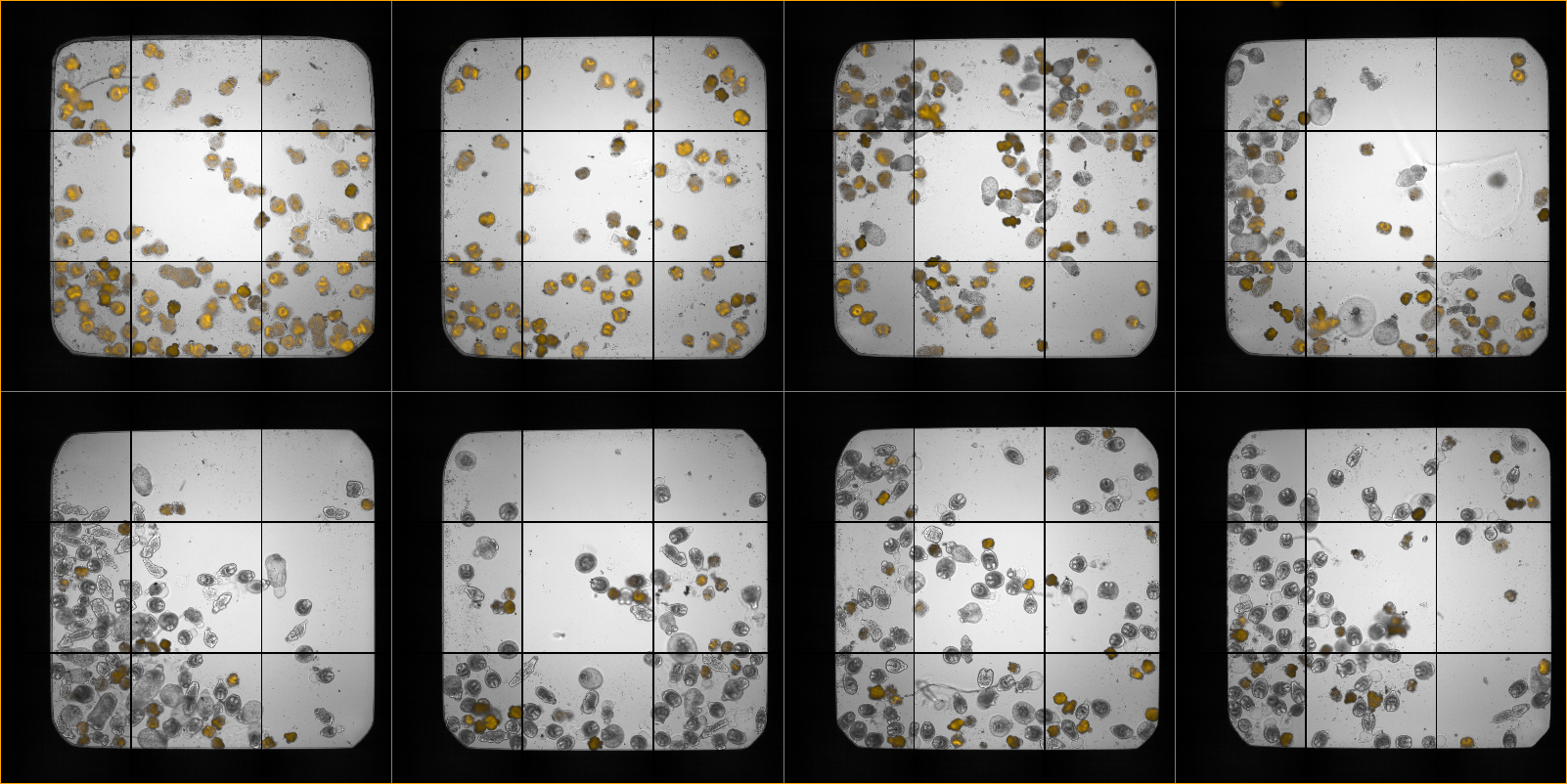

Supplement: Supplementary file 5 — Additional file 5: Dataset S4. PSC images of 16 drug treatment results in a dose–response assay. [file 13071_2024_6456_MOESM5_ESM.zip › Supplementary file 5/Positiv WP1130.png]

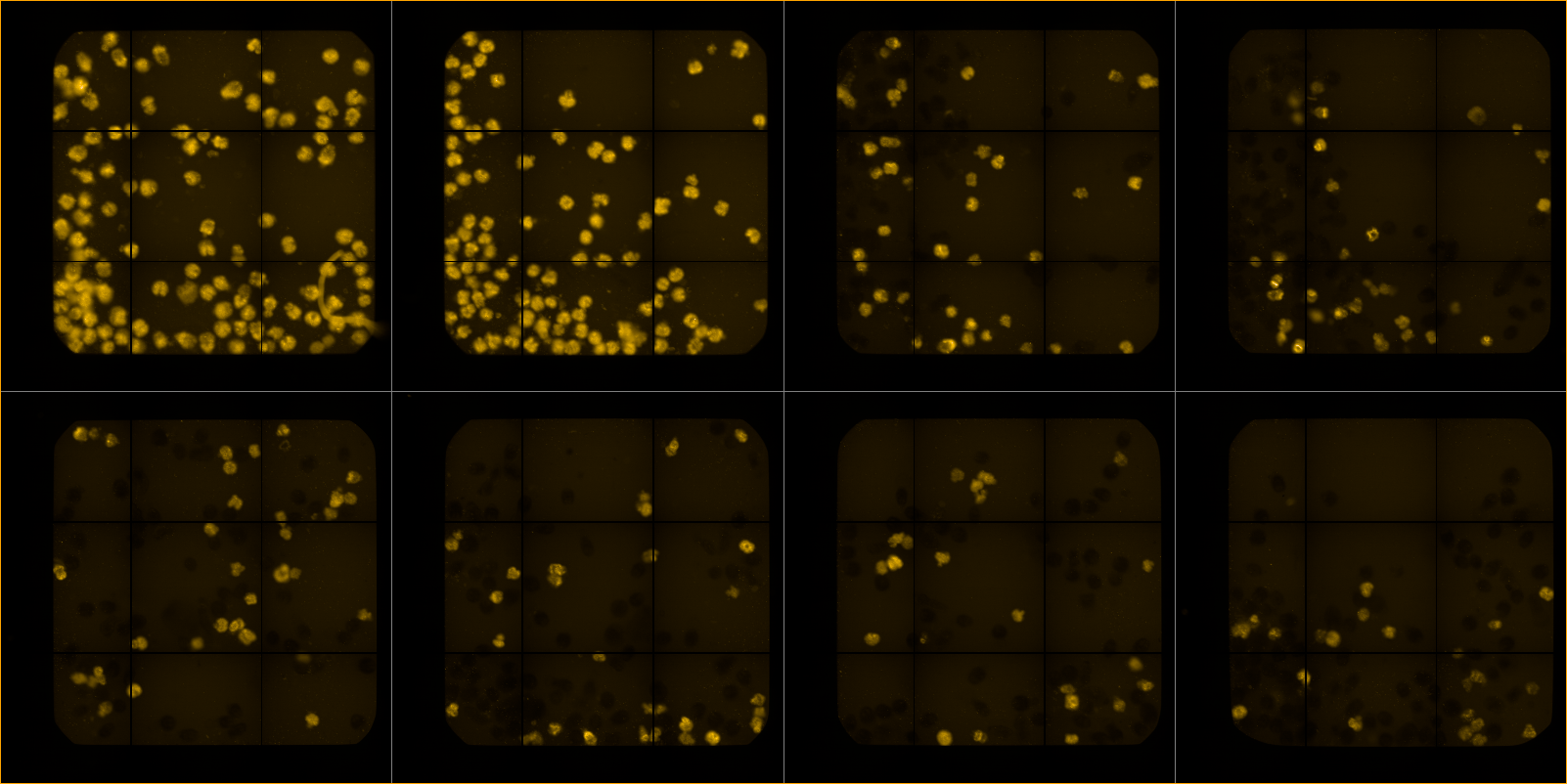

Supplement: Supplementary file 5 — Additional file 5: Dataset S4. PSC images of 16 drug treatment results in a dose–response assay. [file 13071_2024_6456_MOESM5_ESM.zip › Supplementary file 5/Terfenadine-2.png]

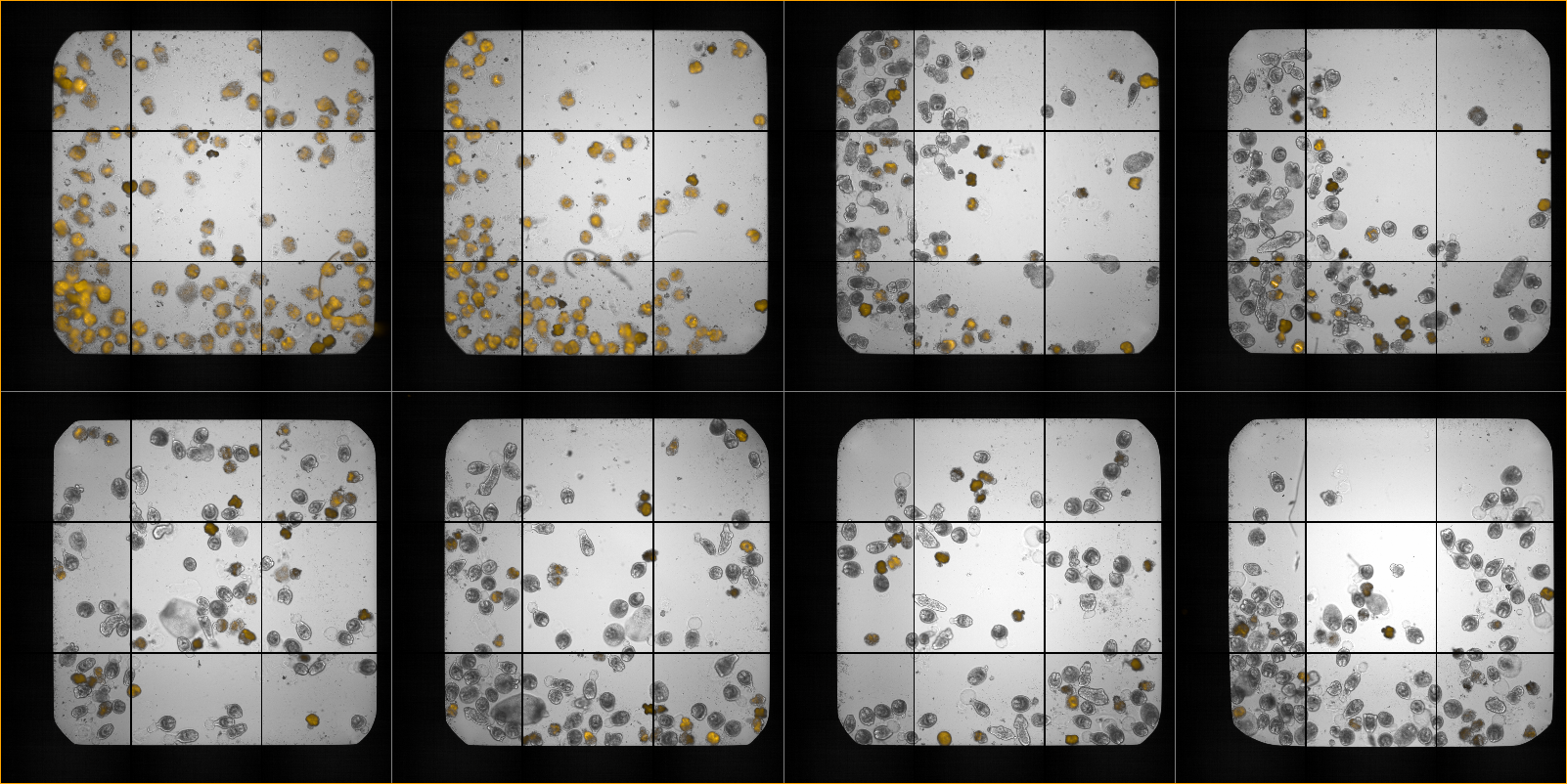

Supplement: Supplementary file 5 — Additional file 5: Dataset S4. PSC images of 16 drug treatment results in a dose–response assay. [file 13071_2024_6456_MOESM5_ESM.zip › Supplementary file 5/Terfenadine.png]

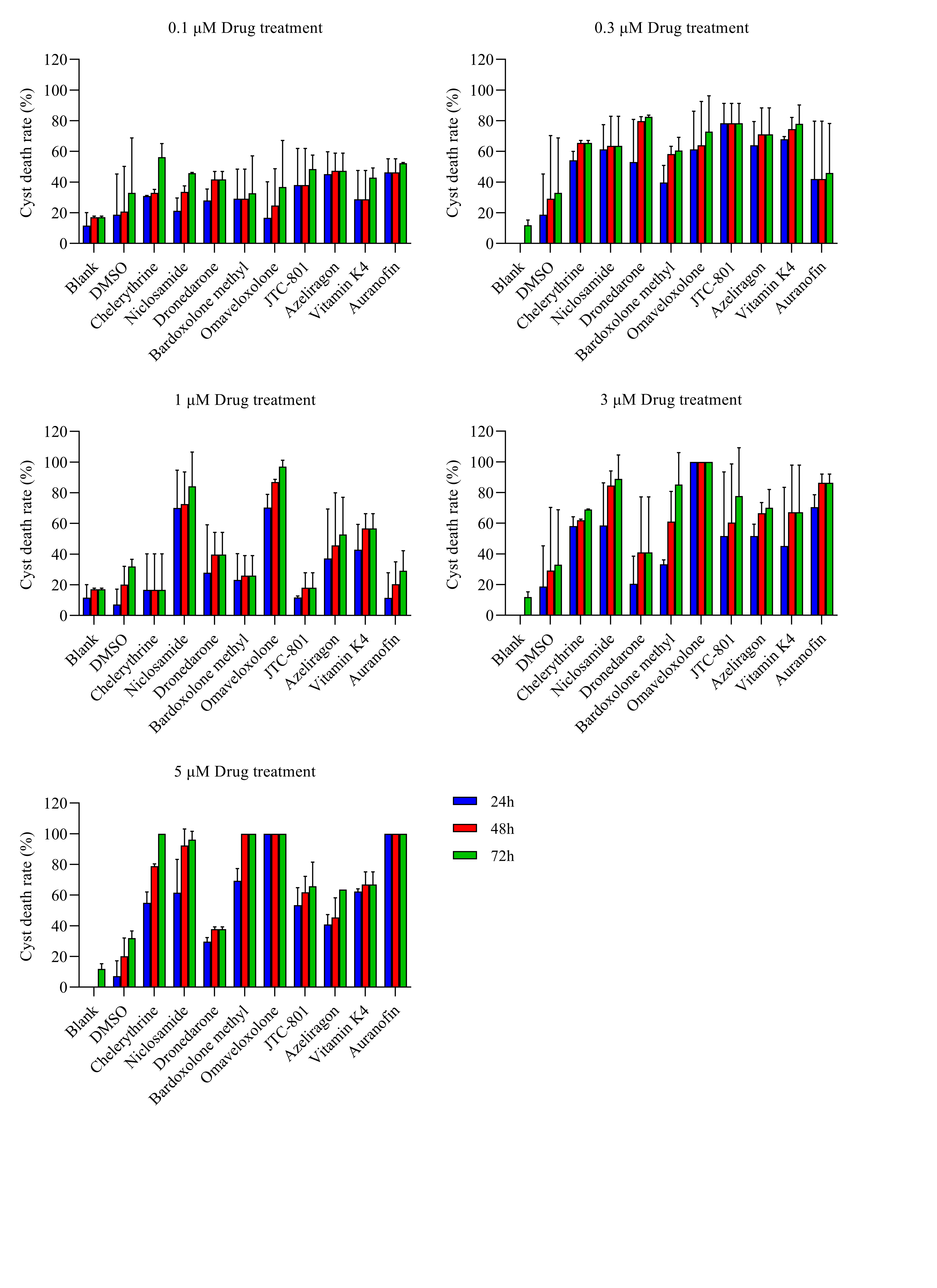

Supplement: Supplementary file 6 — Additional file 6: Figure S1. Drug efficacy in the in vitro cyst viability assay. [file 13071_2024_6456_MOESM6_ESM.tif]
