# Supplementary material for: Repurposing of a library for high-content screening of inhibitors against Echinococcus granulosus
Source: Parasit Vectors. 2024 Sep 3;17:373. doi: 10.1186/s13071-024-06456-6 (PMC11370232; doi:10.1186/s13071-024-06456-6)

ABZ-20um-D7-01

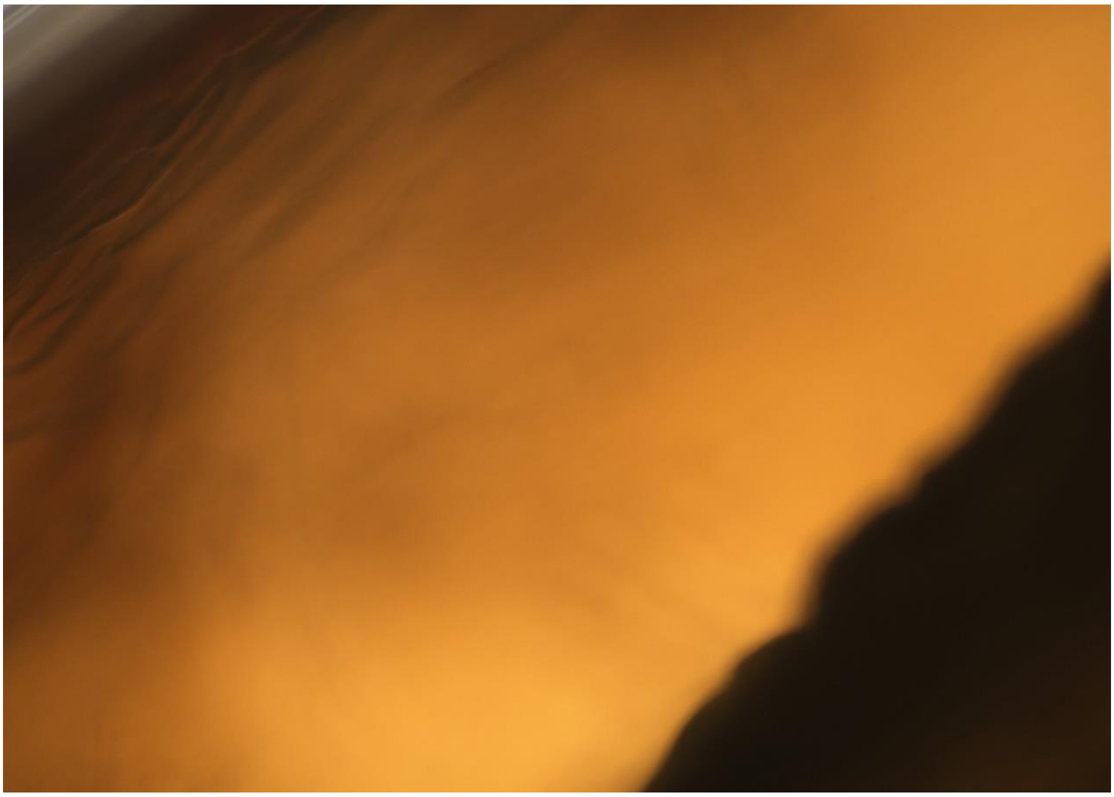

ABZ-20um-D7-02

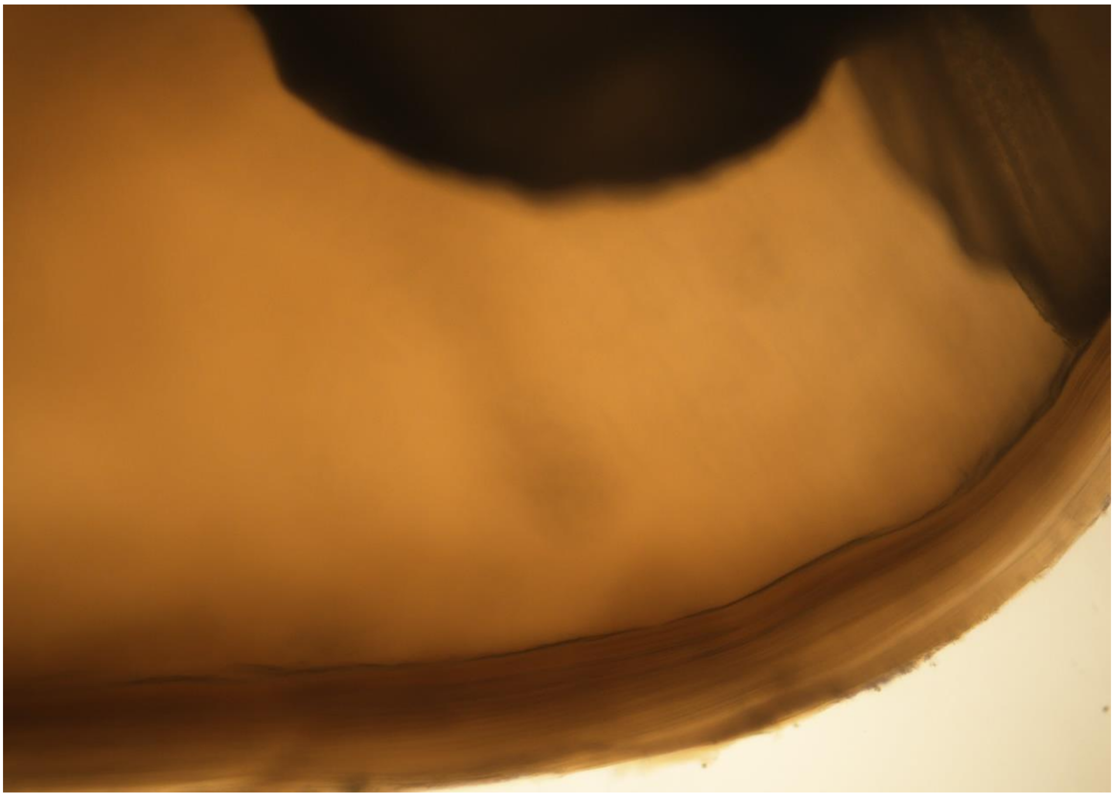

ABZ-20um-D7-03

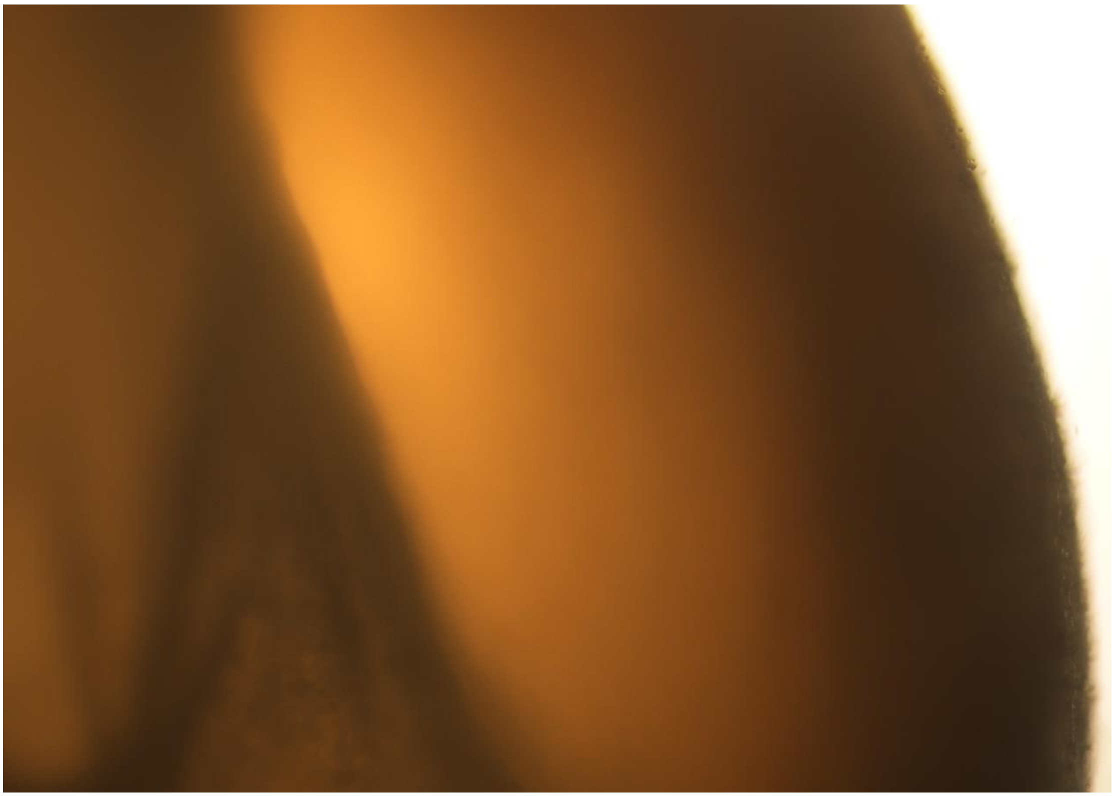

DMSO-D7-01

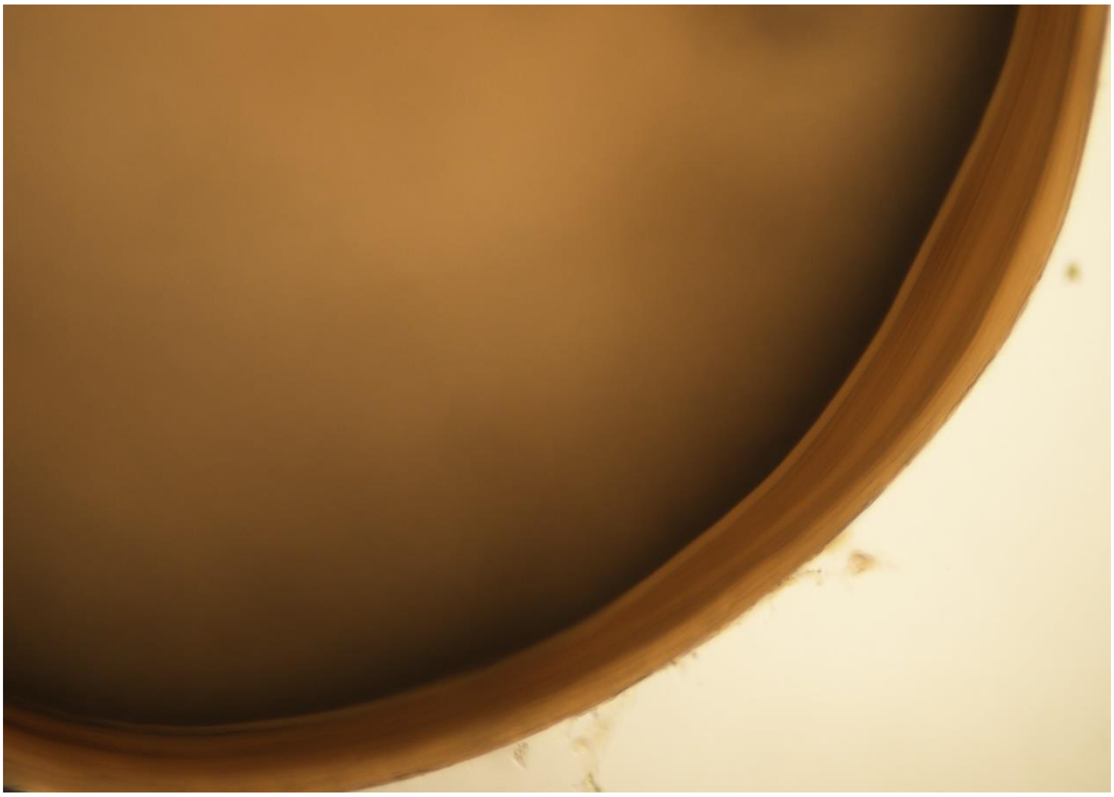

DMSO-D7-02

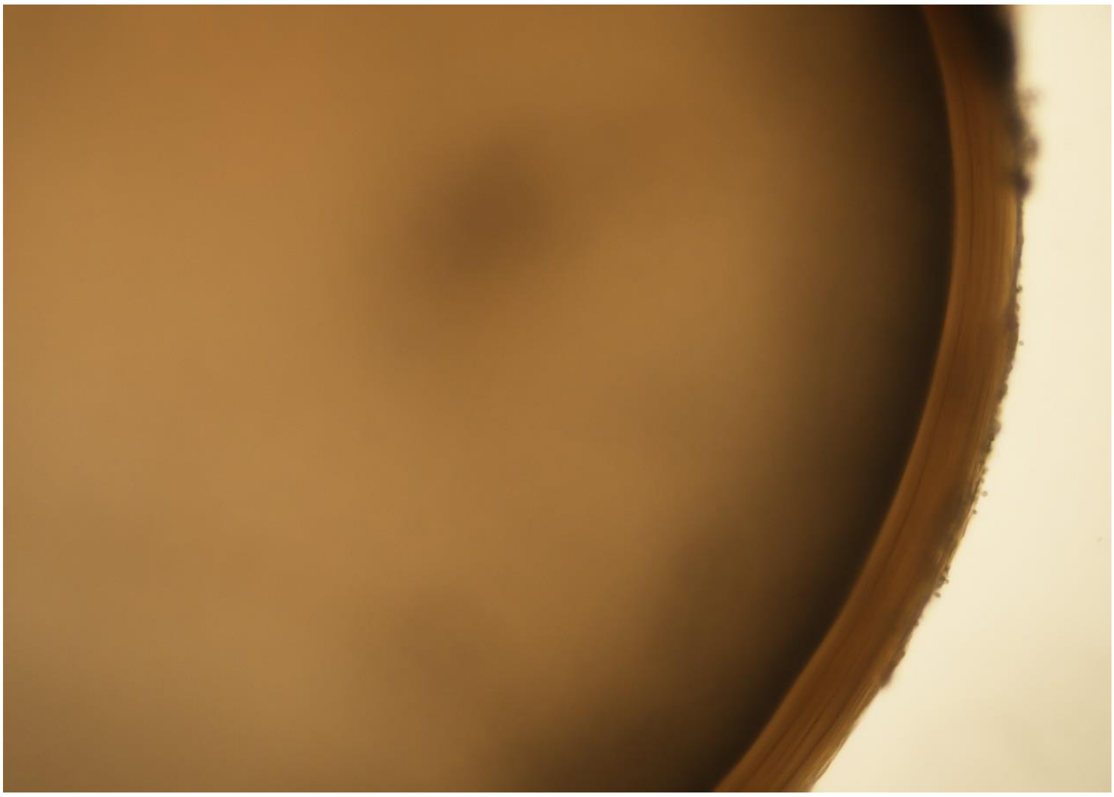

DMSO-D7-03

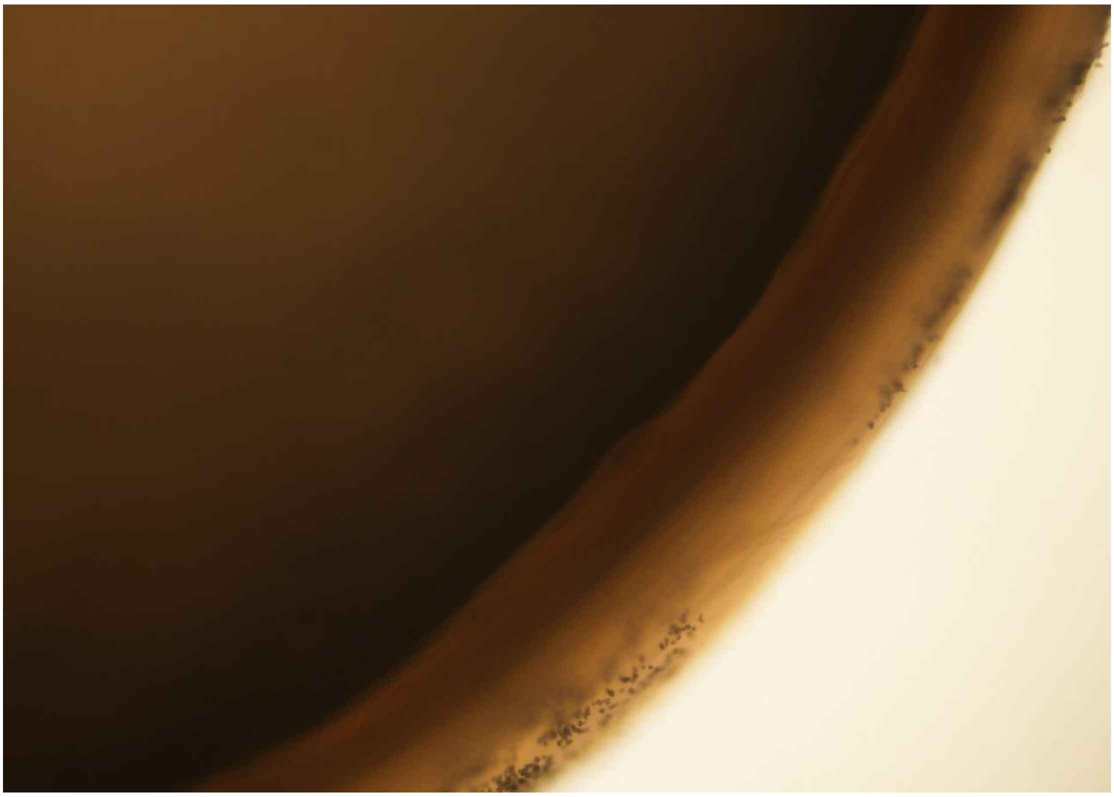

NIC-5um-D7-01

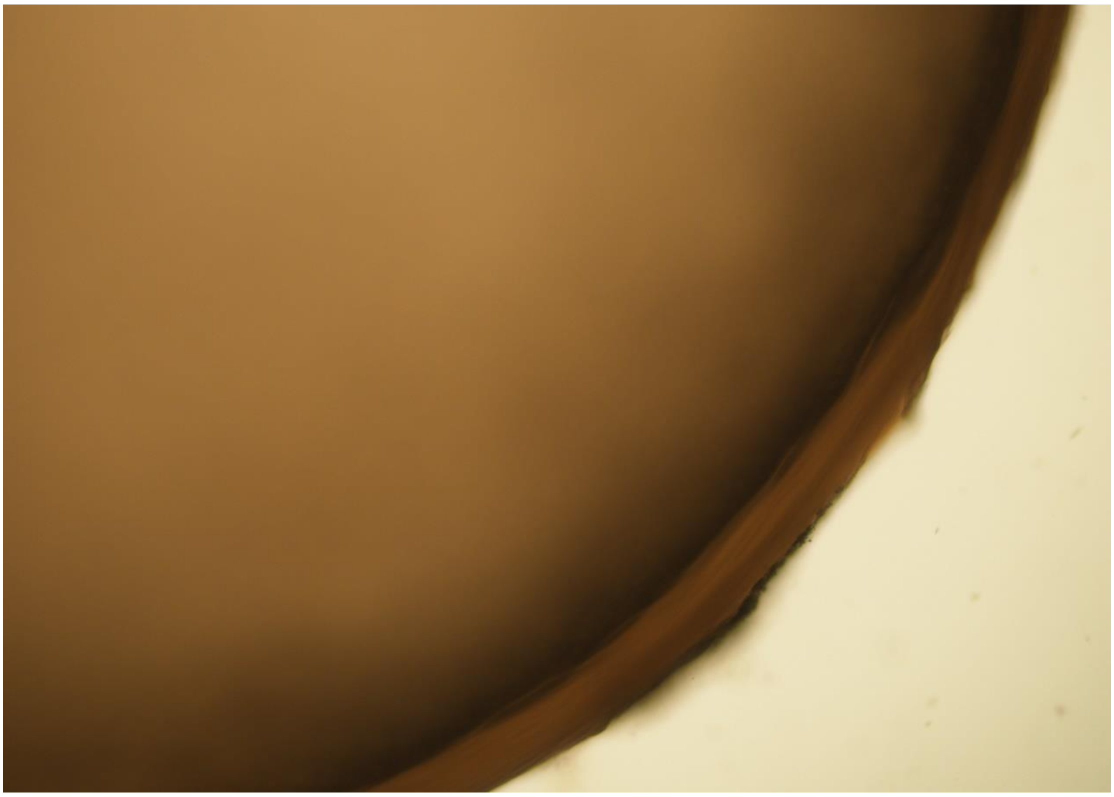

NIC-5um-D7-02

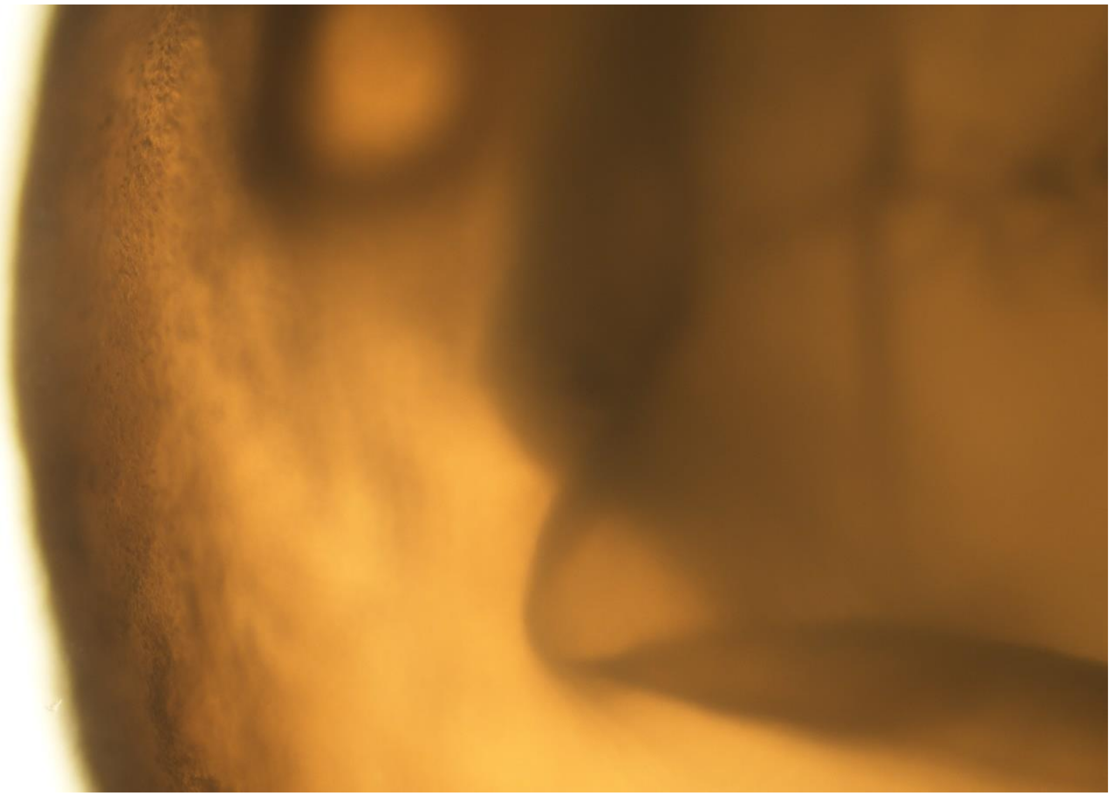

NIC-5um-D7-03

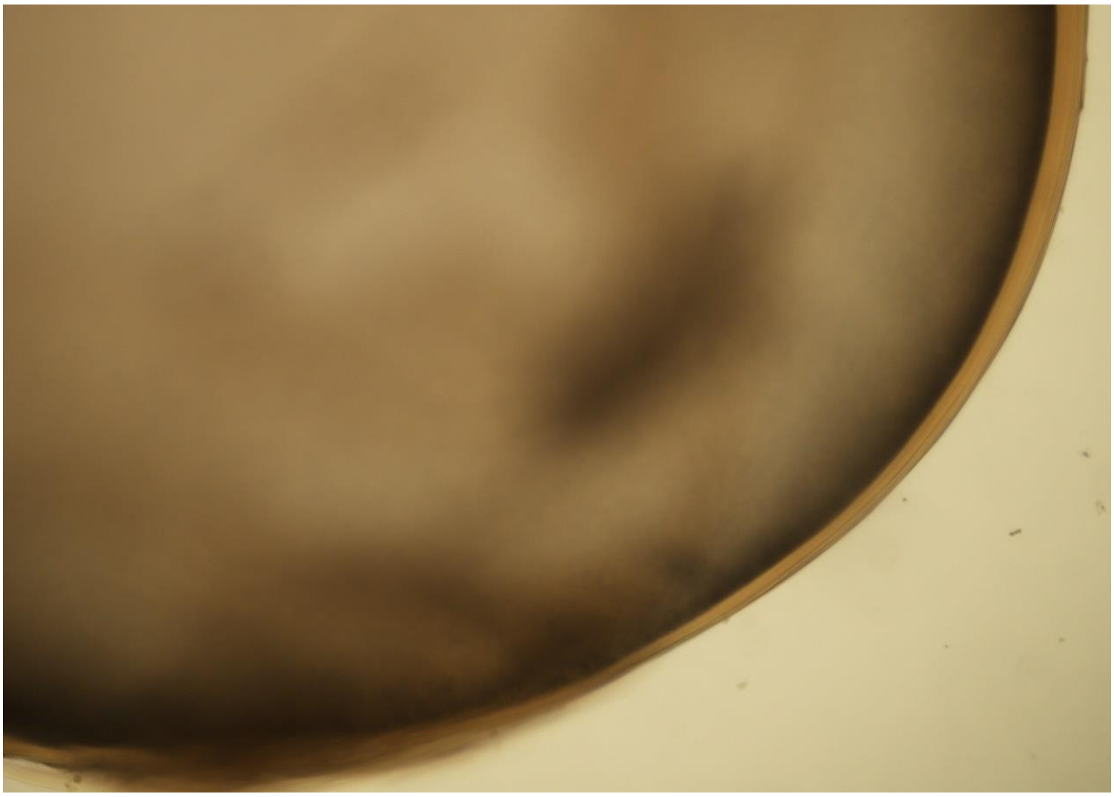

NIC-20um-D7-01

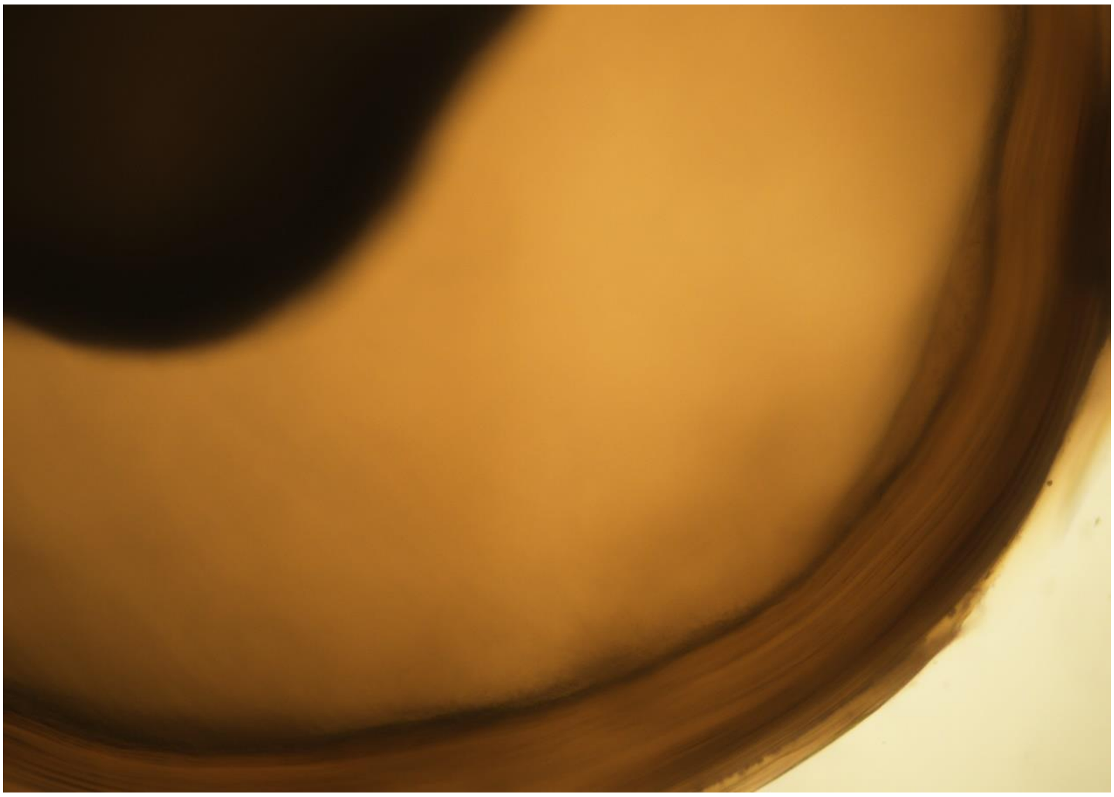

NIC-20um-D7-02

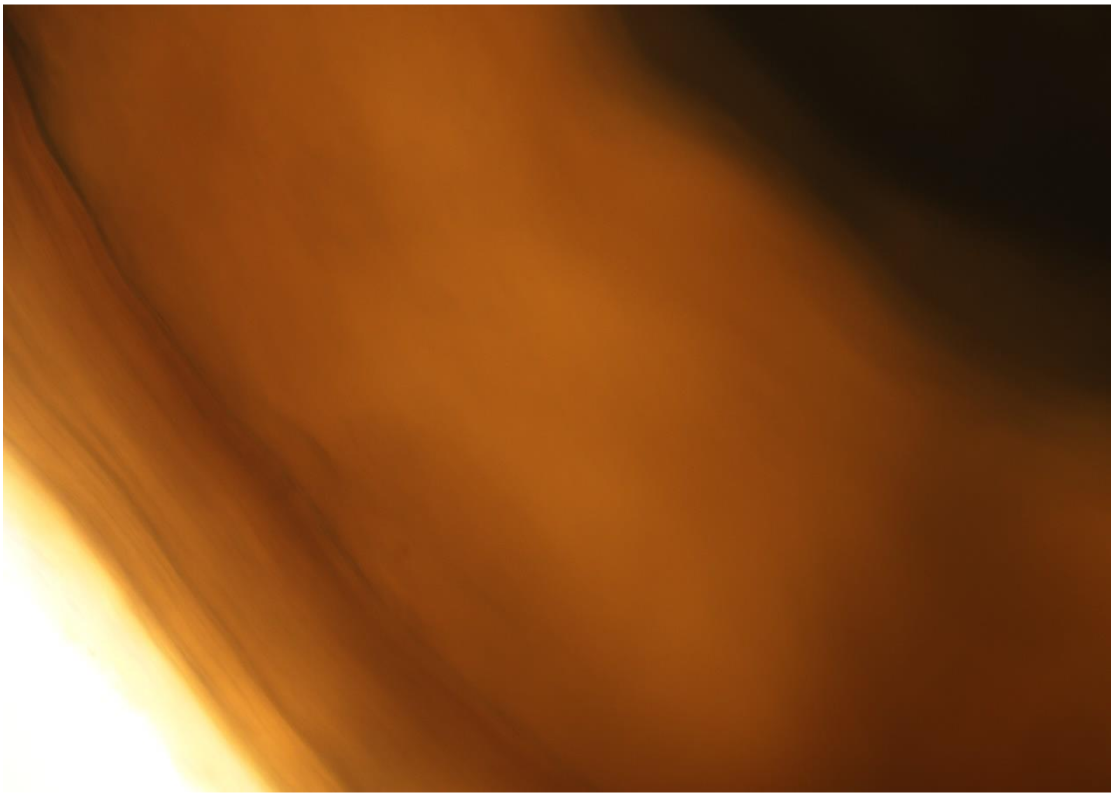

NIC-20um-D7-03

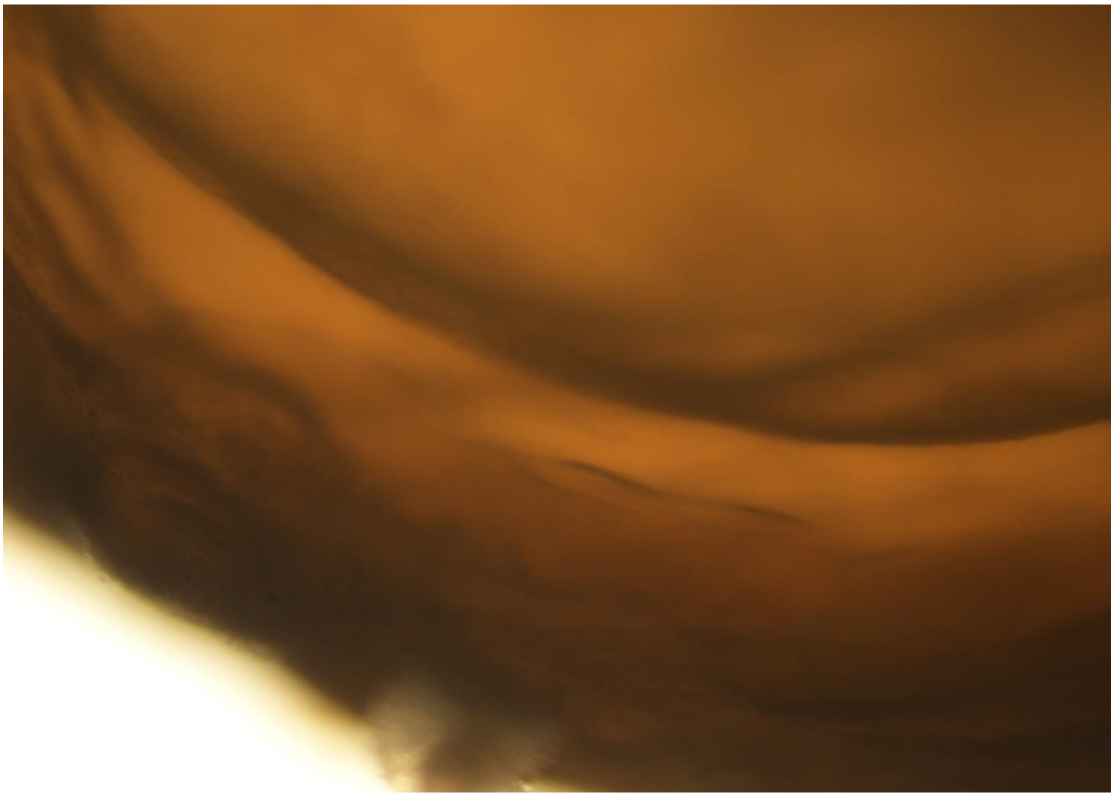

OMA-5um-D7-01

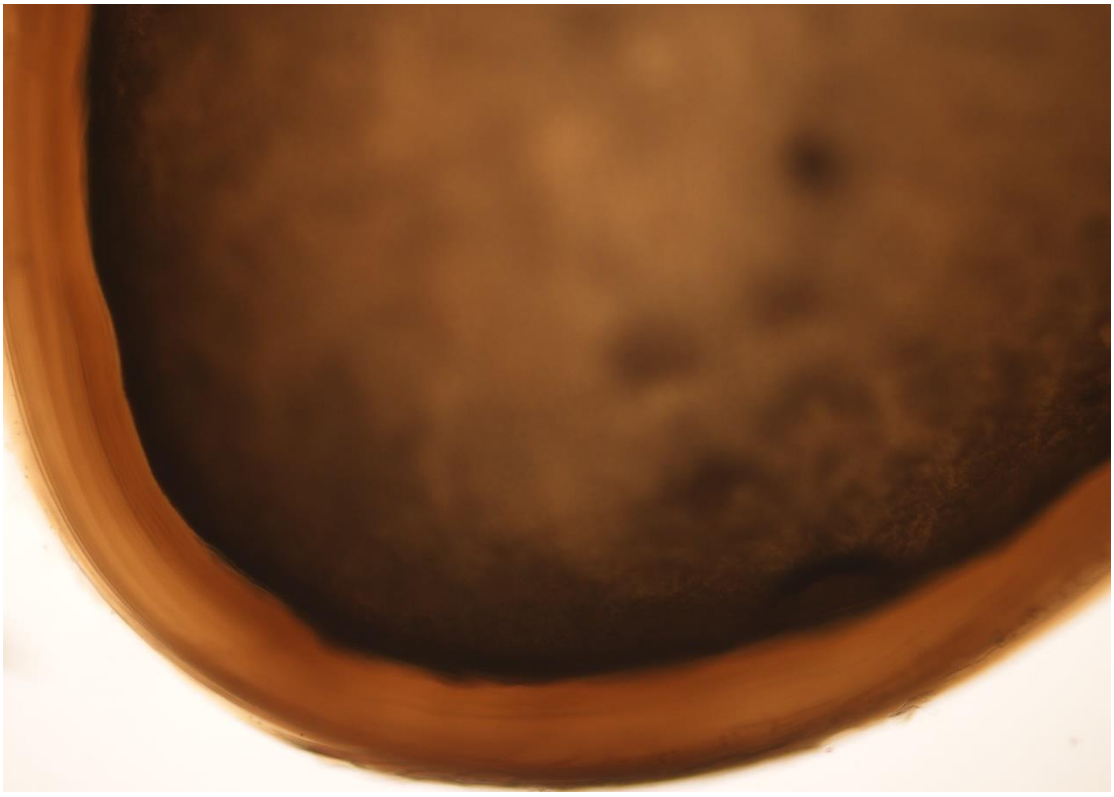

OMA-5um-D7-02

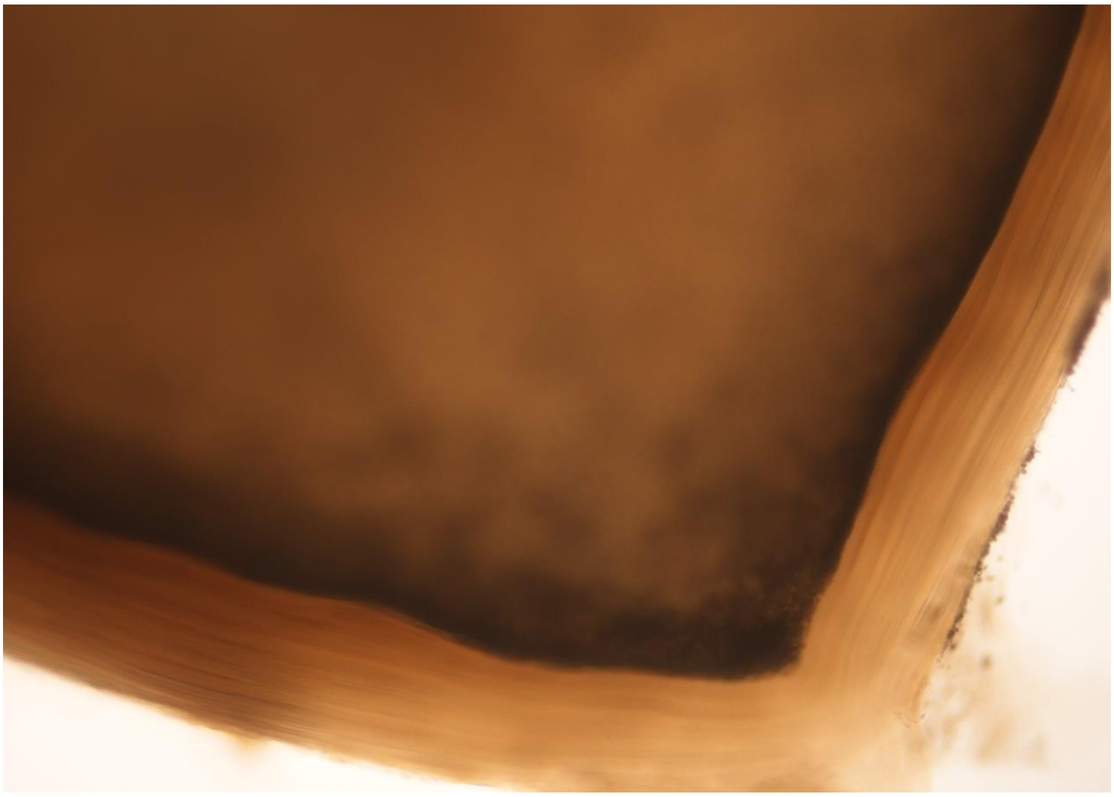

OMA-5um-D7-03

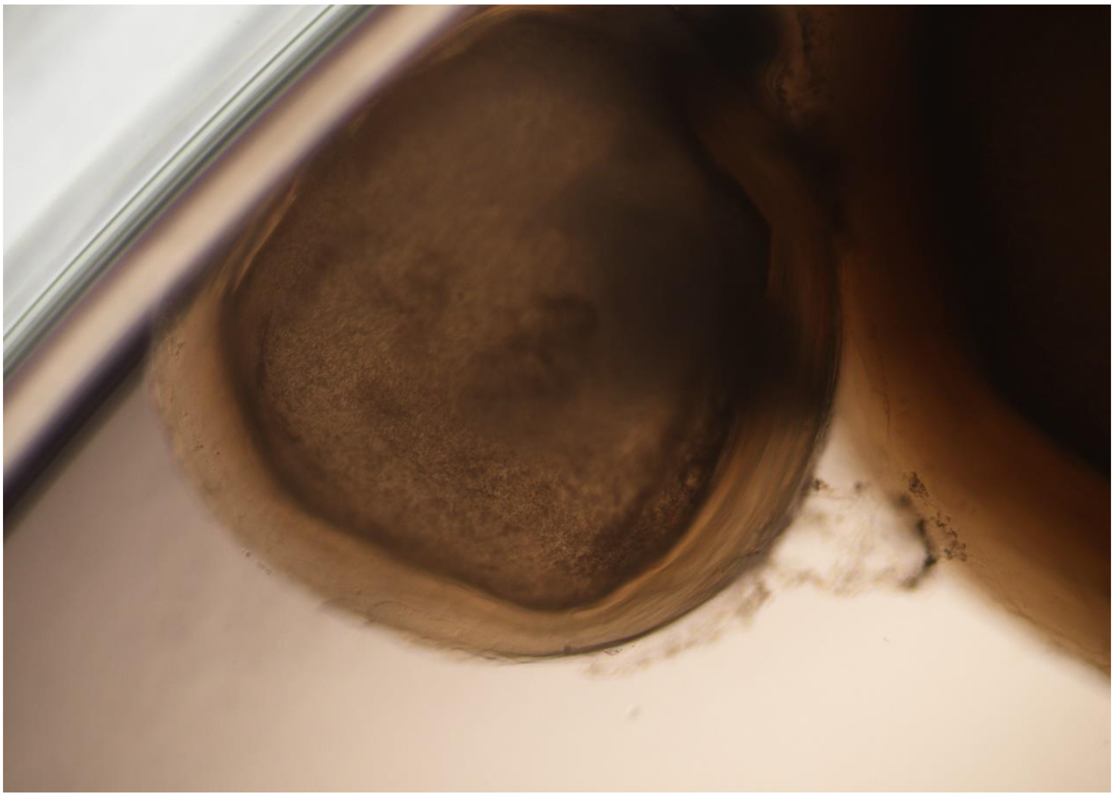

OMA-5um-D7-04

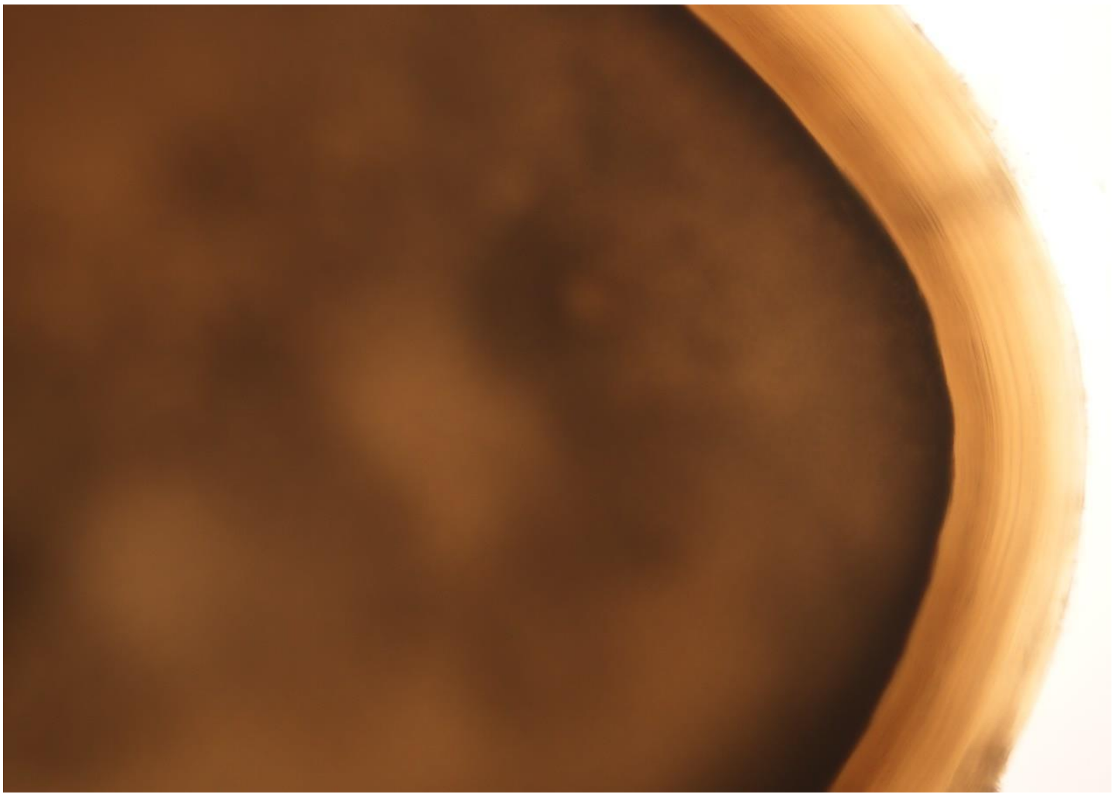

OMA-20um-D7-01

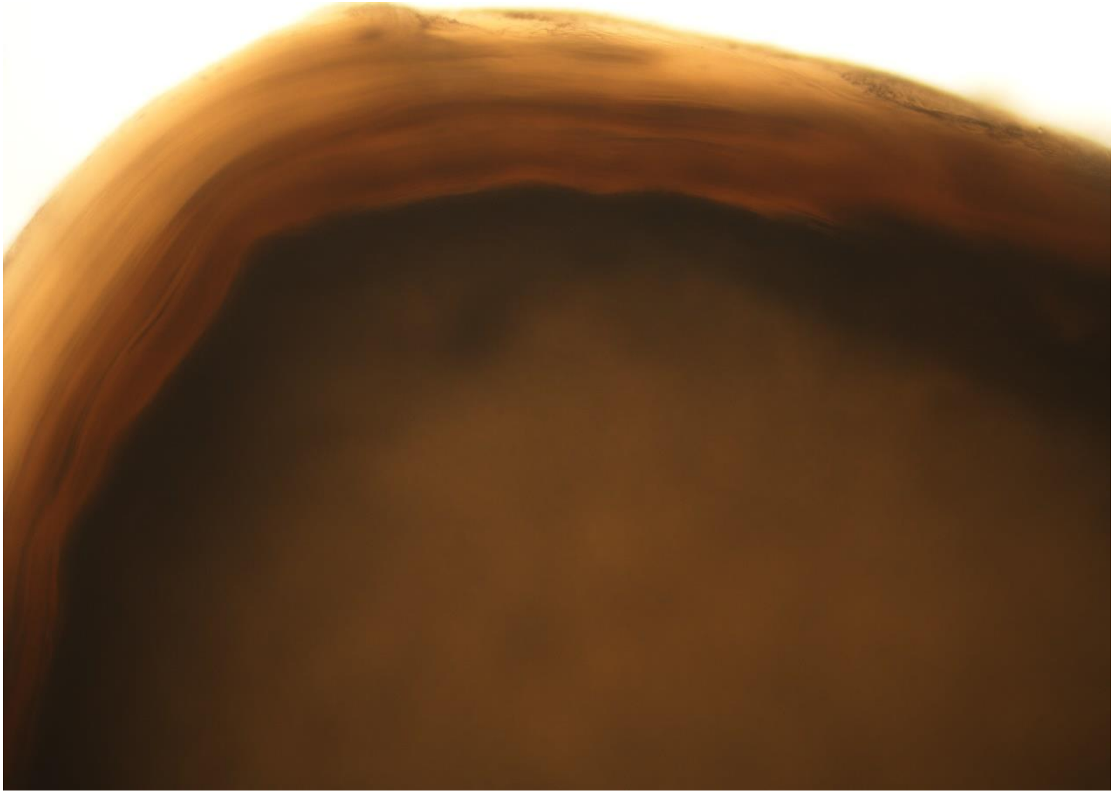

OMA-20um-D7-02

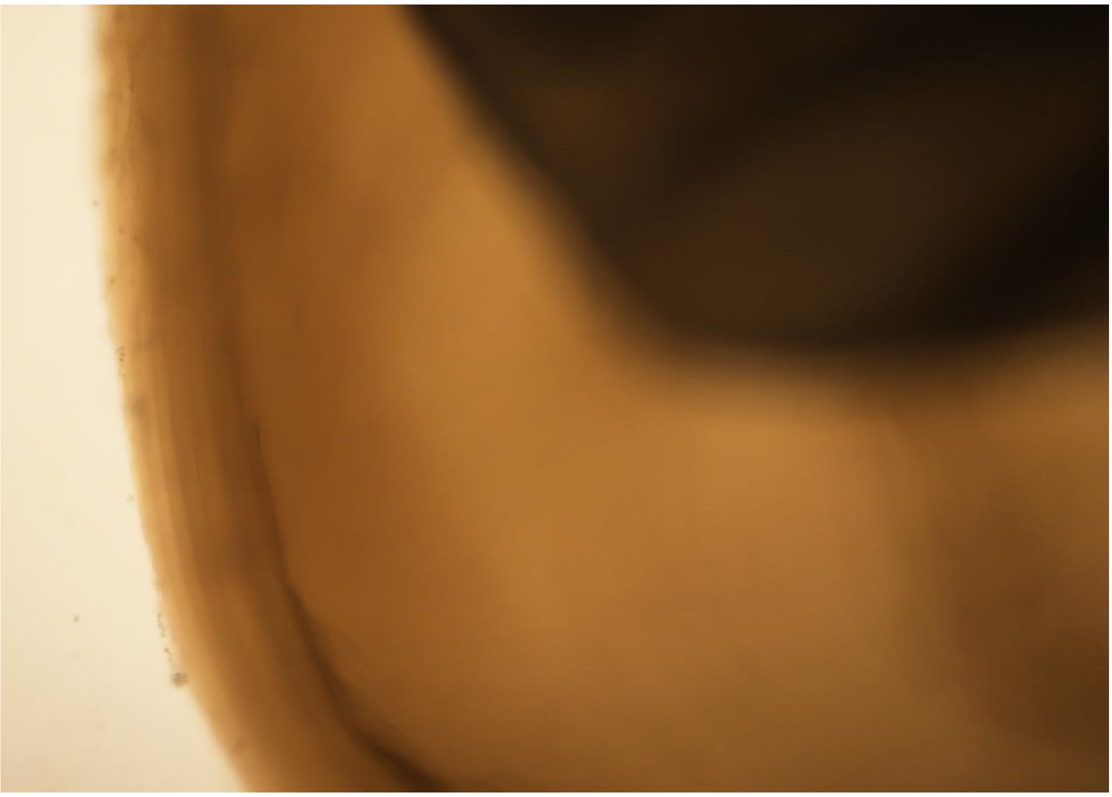

OMA-20um-D7-03

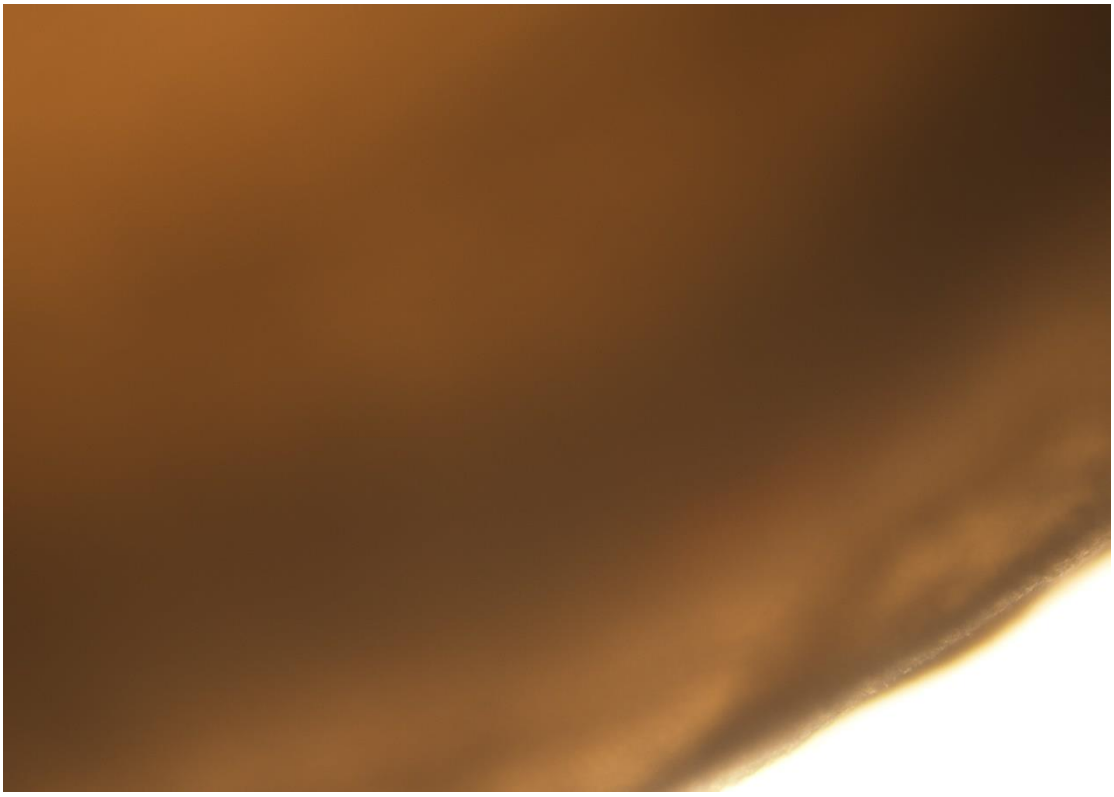

Supplement: Supplementary file 7 — Additional file 7: Figure S2. Raw pictures of cysts in ex vivo cyst viablity study. [file 13071_2024_6456_MOESM7_ESM.pdf]
